# Supplementary material for: Phase III, two arm, multi-centre, open label, parallel-group randomised designed clinical investigation of the use of a personalised early warning decision support system to predict and prevent acute exacerbations of chronic obstructive pulmonary disease: ‘Predict & Prevent AECOPD’ – study protocol
Source: BMJ Open. 2023 Mar 13;13(3):e061050. doi: 10.1136/bmjopen-2022-061050 (PMC10016266; doi:10.1136/bmjopen-2022-061050)
Supplement: Author's manuscript [file bmjopen-2022-061050.draft_revisions.pdf]

BMJ Open

BMJ Open is committed to open peer review. As part of this commitment we make the peer review history of every article we publish publicly available.

When an article is published we post the peer reviewers' comments and the authors' responses online. We also post the versions of the paper that were used during peer review. These are the versions that the peer review comments apply to.

The versions of the paper that follow are the versions that were submitted during the peer review process. They are not the versions of record or the final published versions. They should not be cited or distributed as the published version of this manuscript.

BMJ Open is an open access journal and the full, final, typeset and author-corrected version of record of the manuscript is available on our site with no access controls, subscription charges or pay-per-view fees (http://bmjopen.bmj.com).

If you have any questions on BMJ Open's open peer review process please email info.bmjopen@bmj.com

BMJ Open

A phase III, 2 arm, multi-centre, open label, parallel-group randomised designed clinical investigation of the use of a personalised early warning decision support system to predict and prevent acute exacerbations of Chronic Obstructive Pulmonary Disease - 'Predict & Prevent AECOPD'

Journal:	*BMJ Open*
Manuscript ID	bmjopen-2022-061050
Article Type:	Protocol
Date Submitted by the Author:	01-Feb-2022

Complete List of Authors:	Mehta, Rajnikant ; University of birmingham<br>Kaur, Dalbir; University of Birmingham,<br>Jarrett, Hugh; University of Birmingham<br>Jowett, Sue; University of Birmingham, Health Economics Unit<br>Gale, Nicola K.; University of Birmingham<br>Turner, Alice; University of Birmingham, Institute of Inflammation & Aging; Heart of England NHS Foundation Trust<br>Spiteri, Monica; University Hospitals of North Midlands NHS Trust<br>Patel, Neil; NEPESMO

Keywords:	RESPIRATORY MEDICINE (see Thoracic Medicine), Chronic airways disease < THORACIC MEDICINE, Thoracic medicine < INTERNAL MEDICINE

SCHOLARONE™
Manuscripts

A phase III, 2 arm, multi-centre, open label, parallel-group randomised designed clinical investigation of the use of a personalised early warning decision support system to predict and prevent acute exacerbations of Chronic Obstructive Pulmonary Disease - 'Predict & Prevent AECOPD'

Dalbir Kaur, University of Birmingham, d.kaur@bham.ac.uk

D Kaur, R Mehta, H Jarrett, S Jowett, N Gale, MA Spiteri, N Patel, AM Turner

Dalbir Kaur[1]
Email: d.kaur@bham.ac.uk

Rajnikant Mehta[2]
Email: R.L.Mehta@bham.ac.uk

Hugh Jarrett[3]
Email: h.jarrett@bham.ac.uk

Sue Jowett[4]
Email: S.JOWETT@bham.ac.uk

Nicola Gale[5]
Email: N.Gale@bham.ac.uk

Alice M Turner[6]
Email: A.M.Turner@bham.ac.uk

Monica A Spiteri[7]
Email: Monica.Spiteri@uhnm.nhs.uk

Neil Patel[8]
Email: neil.patel@nepesmo.com

[1]Senior Trials Manager, Birmingham Clinical Trials Unit (BCTU), Institute of Applied Health Research, College of Medical and Dental Sciences, University of Birmingham, Edgbaston, Birmingham, B15 2TT

[2]Senior Medical Statistician, Birmingham Clinical Trials Unit (BCTU), Institute of Applied Health Research, College of Medical and Dental Sciences, University of Birmingham, Edgbaston, Birmingham, B15 2TT

[3]Trials Management Team Leader, Birmingham Clinical Trials Unit (BCTU), Institute of Applied Health Research, College of Medical and Dental Sciences, University of Birmingham, Edgbaston, Birmingham, B15 2TT

[4]Professor of Health Economics, Health Economics Unit, Institute of Applied Health Research, College of Medical and Dental Sciences, University of Birmingham, Edgbaston, Birmingham, B15 2TT

[5]Professor of Health Policy and Sociology, Health Services Management Centre, School of Social Policy Director of Postgraduate Research, College of Social Sciences, University of Birmingham, Edgbaston, Birmingham, B15 2TT

For peer review only

[6] Professor of Respiratory Medicine and Honorary Consultant Respiratory Physician, Institute for Applied Health Research, University of Birmingham, Birmingham, UK, B15 2TT

[7]Director of Respiratory Research, Academic Research Unit, Courtyard Annexe - C Block, Royal Stoke University Hospital, University Hospitals of North Midlands NHS Trust, Newcastle Road, Staffordshire, UK, ST4 6QG

[8]Chief Medical Officer, NEPESMO Ltd, 76 King Street, Manchester, M2 4NH, United Kingdom
Word count: 3487 (excluding title page, abstract, table, legend and references)

Abstract

Introduction

With 65 million cases globally, chronic obstructive pulmonary disease (COPD) is the 4th leading cause of death and imposes a heavy burden on patients' lives and healthcare resources worldwide. Around half of all patients with COPD have frequent (≥2 per year) AECOPD[5]. Rapid readmissions are also common. Exacerbations impact significantly on COPD outcomes, causing significant lung function decline. Prompt exacerbation management optimises recovery and delays the time to the next acute episode.

Methods/analysis

The Predict & Prevent AECOPD trial is a phase III, 2 arm, multi-centre, open label, parallel-group individually randomised clinical trial investigating the use of a personalised early warning decision support system (COPDPredict™) to predict and prevent acute exacerbations of COPD. We aim to recruit 384 participants and randomise each individual in a 1:1 ratio to either SSMP with RM (control arm) or COPDPredict™ with RM (intervention arm)

The trial will inform the future standard of care regarding management of exacerbations in COPD patients. The main outcome measure is to provide further validation, as compared to usual care, for the clinical effectiveness of COPDPredict™ to help guide and support COPD patients and their respective clinical teams in identifying exacerbations early, with an aim to reduce the total number of AECOPD-induced hospital admissions in the 12 months following each patient's randomisation.

Ethics and Dissemination

This study protocol is reported in accordance with the guidance set out in the SPIRIT statement. Predict and Prevent AECOPD has obtained ethical approval in England (19/LO/1939). Upon completion of the trial and publication of results a lay findings summary will be disseminated to trial participants.

Trials Registration: Clinical trials.gov NCT04136418

Keywords: Respiratory, COPD, Exacerbations, Symptom Control Markers, COPDPredict™

For peer review only

NIHR Statement

This work is supported by the National Institute for Health Research (NIHR) under its Invention for Innovation (i4i) Programme (Grant Reference Number NIHR200002). The views expressed are those of the author(s) and not necessarily those of the NHS, the NIHR or the Department of Health.

Strengths and Limitations

- This study will be conducted as a phase 3 randomised controlled trial concerning an intervention for the early warning of COPD exacerbations, COPDPredict™, in patients with COPD. It will provide clinicians with a possible alternative for detecting COPD exacerbations early and actioning timely treatment changes to prevent unnecessary hospitalisation.
- It has the potential to better the patient's quality of life if the burden of AECOPD is reduced. The secondary outcomes will enable conclusions around cost saving for the NHS.
- The study is limited to detecting effects on hospitalisations, and it is possible important changes in other aspects of COPD could be missed, as not powered to detect them.

For peer review only

Background

With 65 million cases globally, chronic obstructive pulmonary disease (COPD) is the 4th leading cause of death and imposes a heavy burden on patients' lives and healthcare resources worldwide[1]. UK death rates are currently double the European Union (EU) average with over 30,000 people dying yearly and annual COPD-related NHS direct costs exceeding £800m. Patients can have acute exacerbations of their COPD (AECOPD), which reduce quality of life, and lead to 140,000 emergency hospital admissions a year in the UK[2]. Indeed, AECOPD remain the 2nd commonest cause of emergency hospital admissions and 1 in 3 of these patients will be readmitted within 3 months[3,4]. Hospitalisation itself carries a poor prognosis with an increased mortality risk.

Around half of all patients with COPD have frequent (≥2 per year) AECOPD[5]. Rapid readmissions are also common – the national COPD audit has shown that 43% of patients with COPD who were admitted are back in hospital within 90 days[7], and up to 71% by 12 months[8]. Exacerbations impact significantly on COPD outcomes, causing significant lung function decline[13]. Prompt exacerbation management optimises recovery and delays the time to the next acute episode[14], with NICE COPD guidelines highlighting a time window (prodrome) between an initial exacerbation's symptoms/signs and subsequent hospitalisation. Within this prodrome there is an opportunity to intervene. Current practice for COPD patients is that they are encouraged to recognise AECOPD, via standard self-management plans (SSMPs), and treat, using rescue medication (RM), but in many cases because of day-to-day variability in symptoms, the start of an exacerbation goes unrecognised and untreated. This inability/uncertainty to recognise and treat exacerbations in their early phase can lead to hospital admissions, which may drive long term decline.

Pharmacological and non-pharmacological treatments are used to reduce COPD exacerbations[15,16], and improve care, such as pulmonary rehabilitation (PR[17]), inhaled bronchodilators and inhaled corticosteroids. SSMPs help patients but have not really shown significant impact on A&E visits or hospital admissions as demonstrated by a systematic review of self-management strategies for

For peer review only

COPD[18]. Intuitively early recognition and treatment of AECOPD would reduce exacerbation severity and duration, and improve prognosis; evidence for this is limited but supportive[19].

This study looks to address the problem by personalising and thus optimising effectiveness of AECOPD management using the CE marked and MHRA registered solution, COPDPredict™[20].

Methods

Patient and public involvement

Throughout the design and delivery of the trial public and participant involvement (PPI) has been accessed. In particular independent members on the trial steering committee provide oversight and contributions to the trial, including design and standardisation of trial-specific SSMP. PPI involvement has also extended to the qualitative research where the topic guide was developed in conjunction with respiratory participant groups.

Trial Design

The Predict & Prevent AECOPD trial is a phase III, 2 arm, multi-centre, open label, parallel-group individually randomised clinical trial investigating the use of a personalised early warning decision support system (COPDPredict™) to predict and prevent acute exacerbations of COPD. We aim to recruit 384 participants from NHS hospitals in the UK and randomise each individual in a 1:1 ratio to either SSMP with RM (control arm) or COPDPredict™ with RM (intervention arm) (*Figure 1*).

The main outcome measure is to provide further validation, as compared to usual care, for the clinical effectiveness of COPDPredict™ to help guide and support COPD patients and their respective clinical teams in identifying exacerbations early, with an aim to reduce the total number of AECOPD-induced hospital admissions in the 12 months following each patient's randomisation. The trial includes a health economic analysis examining health-related quality of life (HRQoL) using the validated questionnaire EQ-5D-5L and healthcare utilisation (as determined by a questionnaire that inquires as to hospitalisations, GP attendances and medication usage). A qualitative sub-study will

For peer review only

assess both the end user experience and the healthcare provider experience, using Normalization Process Theory to understand the implementation and integration of the new technology[21].

Eligibility Criteria

Inclusion Criteria

- Clinically diagnosed COPD, confirmed by post-bronchodilator spirometry and defined as Post bronchodilator Forced expiratory volume/Forced vital capacity (FEV_1/FVC) <0.7 and <lower limit of normal, in the stable state
- ≥1 AECOPD in any 12-month period within the last 2 years or ≥1 hospital admission for AECOPD in the previous two years.
- Exacerbation free for at least 6 weeks
- An age of at least 18 years
- Willing and able to comply with data collection to 12 months from randomisation
- Ability to consent
- Ability to use intervention as judged by the investigator at screening, upon demonstration of the system to the patient

Exclusion Criteria

- Life expectancy <12 months
- Co-enrolment into any clinical trials of investigative medicinal product (CTIMPs)
- Patients with selected co-morbidities, where these could impair use of the intervention (eg dementia)

Outcomes

Primary Outcome

Number of hospital admissions at 12 months post randomisation where the primary reason for admission is AECOPD. This will be obtained from participant testimony and from centrally held (HES)

For peer review only

records. Data from the 2 sources will be cross-referenced to remove double-counting. The total number of admissions will be the sum of unique admissions from the 2 sources of data

Secondary Outcomes

Clinical Outcomes

Over a 12-month period, following randomisation;

Total in participant days, Number of visits to A&E, Number of participant defined exacerbations, Appropriate action on symptoms of AECOPD, as shown by the app, or participant testimony, Health-related quality of life (CAT) and, FEV_1 at 12 months post randomisation (from spirometry obtained during study visit)

Economic Outcomes

Health-related quality of life at baseline and 3, 6, 9 and 12-months post randomisation (EQ-5D-5L)

Healthcare utilisation at 3, 6, 9 and 12-months post randomisation, as determined by a questionnaire that inquires on hospitalisations, GP attendances and medication usage

Qualitative sub-study

End user experience and provider experience

Control Arm

A standard self-management plan (SSMP) alongside use of rescue medication (RM) containing 5 days of antibiotic and steroid treatment.

Intervention

COPDPredict™, consists of a patient-facing App (iOS/Android) and clinician-facing dashboard[22]. The Early Warning Decision Support System uses longitudinal remote monitoring of relevant subjective and objective data (symptoms, spirometry, biomarkers) in real-time via the App which is connected to CE-marked Bluetooth-enabled sensor peripherals. These data are used to construct COPD-relevant individual profiles such that artificial intelligence artificial intelligence (AI)-driven algorithms

For peer review only

can then identify changes in health status to provide timely individualised alerts to patients and clinicians and sign-posting to action plans for patients.

COPDPredict™ also provides information around COPD self-management, pulmonary rehabilitation, inhaler technique and utilises gamification to help with adherence. Patients have full access to their results and can also directly message their clinical team. COPDPredict™ may signpost a participant to a self-management action plan for exacerbation management but clinical team supervision and oversight will remain throughout, as per usual care.

The clinician facing dashboard allows for “real-time” case management with the ability to remotely monitor patients and facilitate interaction. Clinicians can choose to escalate treatments based on the results being transmitted by the patients. A crucial dimension to this advanced system is that blood biomarker measurements which inform an algorithm are incorporated to enhance accuracy. Blood-based point of care (POC) analysers are used with COPDPredict™ and these POC analysers have previously been validated against laboratory CRP measures.

Recruitment and Consent

All patients must provide fully informed consent. A Participant Information Sheet (PIS) which explains the different ways in which the participant may enter the trial is provided to participants to facilitate this process. Investigators or delegate(s) adequately explain the aim, trial intervention, anticipated benefits and potential hazards of taking part in the trial to the participant. They also stress that participation is voluntary and that the participant is free to refuse to take part and may withdraw from the trial at any time. Any trial specific procedures are completed after the participant has given informed consent. At each visit the participant’s willingness to continue in the trial is ascertained and documented in the medical notes. Where new information becomes available which may affect the participants’ decision to continue, participants will be given time to consider and if happy to continue will be re-consented. Re-consent will be documented in the medical notes.

For peer review only

Randomisation

After informed consent has been obtained and full participant eligibility confirmed (including exacerbation free for 6 weeks), the participant can be randomised into the trial. Randomisation CRFs are provided to investigators and may be used to collate the necessary information prior to randomisation. Participants are randomised by computer/telephone at the level of the individual in a 1:1 ratio to either SSMP with RM (control arm) or COPDPredict™ with RM (intervention arm).

A minimisation algorithm will be used within the online randomisation system to ensure balance in the treatment allocation over centre, age (<60, ≥60 years) and severity of disease as per Global Initiative for Obstructive Lung Disease (GOLD)[1].

The participants are randomised to one of the following:

Control Arm

A standard self-management plan (SSMP) alongside use of rescue medication (RM) containing 5 days of antibiotic and steroid treatment.

Intervention arm

Supported self-management using the COPDPredict™ App, involving personalised alerts to both patients and the clinical care team.

A ‘random element’ will be included in the minimisation algorithm, so that each participant has a probability, of being randomised to the opposite treatment that they would have otherwise received. Once randomised patients will also be asked to complete baseline quality of life booklet which contains both the EQ-5D-5L and CAT questionnaire.

For peer review only

Assessment schedule

Visit	Hospital Discharge/ Initial appointment	Personal Baseline Day 1 after 6 weeks exacerbation free	Personal Baseline Day 7 (±2 days)	End of Personal Baseline Period	Approximately 4 times during any exacerbation (home visit or clinic)	Weekly until Week 52	Week 13 Telephone follow Up +/-1 week	Week 26 Hospital/ Telephone follow Up +/-1 week	Week 39 Telephone follow Up +/-1 week	Week 52 Telephone /Hospital follow Up +/- 2 weeks	Patients daily wellbeing completion post two-week bassline period
Eligibility check	X										
Valid informed consent	X										
Randomisation		X									
Height and Weight	X									X	
Concomitant medication	X	X		X				X	X	X	
Baseline medical history taken[α]	X										
Request to GP for rescue medication	X						(X)	(X)	(X)		
Assessment of (S)AEs	X				(X)		X	X	X	X	

er review only

Questionnaires (QoL and health utilisation)	X						X	X	X	X	
Spirometry	X							X		X	
Intervention only below this line					Intervention only below this line						
Provision of equipment & participant training		X									
Spirometry	X	X	X		(X)	X					
Symptom control markers	X	X	X	X	(X)	X	X	X	X	X	
Point of care blood test		X		X	(X)						
Clinical histories		X		X			X	X	X	X	
Wellbeing (App)											X

(X) denotes only when required. If rescue medication has not been used, fresh prescription requests will not be necessary

During the baseline period and any subsequent exacerbations patients will be entering well-being data on the App which will be related to lifestyle choices and the end-user experience. This data will not form part of the formal analysis of quality of life.

Table 1: Schedule of assessments

For peer review only

Statistical Analysis

Sample size

The justification of the sample size is based on previous evidence[23] that had shown a mean estimate of 2.5 COPD admissions in the previous year in the control group. To detect a difference of 1 admission in the mean number of admissions between groups using the standard methods of difference between means and assuming standard deviation of 2.6[23] with 90% power and a type I error rate of 5% (two-sided), 144 participants per group will need to be randomised, 288 in total. Assuming and adjusting for a 25% loss to follow-up/ drop-out rate, 384 participants will need to be recruited.

Analysis of Outcome Measures

The primary comparison groups will be composed of those randomised to the use of COPDPredict™ system and those randomised to the standard self-management plan (SSMP). All analyses will be based on the intention to treat principle[24], with all patients analysed in the treatment groups to which they were allocated irrespective of compliance with the randomised allocated treatment, and all patients will be included in the analyses. For all outcomes, summary statistics (e.g. mean differences, relative risks) will be reported and 95% confidence intervals will be constructed where appropriate. A two-sided p-value of <0.05 will be considered statistically significant.

Primary Outcome Measure

The primary analysis for this study will be to compare rates of hospital admission between the treatment groups (COPDPredict™ versus Usual care) over 12 months, following randomisation, where the primary reason for admission is an AECOPD event. These hospital admissions per person will be analysed using Poisson regression models adjusting for treatment group and minimisation variables. If there is over dispersion a negative binomial regression model adjusting for treatment group and the same minimisation variables will be taken into consideration. Point estimates

For peer review only - http://bmjopen.bmj.com/site/about/guidelines.xhtml

(incidence rates) will be provided and accompanied with 95% confidence intervals and respective p-values.

Secondary Outcome Measures

Recorded observations taken over a 12-month period, following randomisation:

Total in-hospital days and FEV_1 – These variables will be summarised using basic descriptive statistics (Mean, SD)). We may also consider a mixed linear regression model, to estimate differences between the intervention group supported with 95% CI adjusting for baseline variables and minimisation variables (centre being a random effects variable).

Health-related quality of life questionnaires - EQ5D-5L and CAT will be converted into scores and analysed using a mixed linear regression model, adjusting for the intervention group, baseline and minimisation variables as before.

Number of A&E visits, participant defined exacerbations and healthcare utilisation - Where the participant experienced an episode, the data during the 12 months from randomisation will be analysed using mixed effects log-binomial regression techniques with presentation of relative risk and 95% confidence intervals. Furthermore, sensitivity analysis based on patients' experience of multiple visits to A&E and participant defined exacerbation will be calculated using Poisson regression techniques and relative risk supported with 95% CI.

Symptom control marker association to clinical decision - The diagnostic accuracy of COPDPredict™ and Usual care will be evaluated by calculating sensitivity, specificity, positive and negative predictive values, and area under curve (AUC)/receiver operating characteristics (ROC) together with 95% CI.

Subgroup analyses

Subgroup analyses will be limited to the same variables used in the minimisation algorithm, apart from centre. Tests for statistical heterogeneity (e.g. by including the treatment group by subgroup

For peer review only

interaction parameter in the statistical model) will be performed prior to any examination of effect estimate within subgroups. The results of subgroup analyses will be treated with caution and will be used for the purposes of hypothesis generation only.

Missing data and sensitivity analyses

Participants with missing primary outcome data will not be included in the primary analysis in the first instance. This presents a risk of bias, and sensitivity analyses will be undertaken to assess the possible impact. This will consist of simulating the missing response using a multiple imputation approach[25]. Parameters used to simulate the missing response will include the minimisation variables, intervention group and previous response. Full details are included in the statistical analysis plan (SAP).

Adverse events and serious adverse events

Patients with COPD can have high disease burden, such that the trial population will be older, with co-morbidities such as osteoporosis[26], cardiovascular disease or raised cardiovascular risk[27,28], along with associated complications and symptoms including abnormal lab results. A relatively high number of adverse events are anticipated as a result of the patients' existing past medical history. However, this study is examining the role of increased participant surveillance and therefore few, if any, foreseeable risks of direct harm associated with the study intervention are anticipated. AE reporting will therefore be limited to those events which are required for trial monitoring or outcome assessment.

All events which meet the definition of serious must be recorded in the participant notes, but for trial purposes these following events do not require reporting on the SAE Form. Such events are "safety reporting exempt".

• Planned hospitalisation for a pre-existing condition, or a procedure required by the trial protocol without serious deterioration in health, is not considered a serious adverse event.

For peer review only - http://bmjopen.bmj.com/site/about/guidelines.xhtml

• An overnight stay in hospital that is due to transportation, organisational or accommodation problems, and without medical background SAES requiring expedited reporting

Any other events that fulfil the usual definition of an SAE will be reported immediately.

Any death occurring during the Trial Protocol defined follow up period (12 months) must be reported as an SAE within 24 hours of the local investigator becoming aware of the event.

Health economics

Within -trial analysis

The economic evaluation alongside the trial will take the form of an incremental cost-effectiveness analysis to estimate cost per hospital admission avoided and incremental cost-utility analysis to estimate cost per quality adjusted life year (QALY) gained. Base-case analyses will be from a healthcare perspective over 12 months follow-up using individual participant level data on costs and outcomes from the clinical trial. Additional analyses will be undertaken from a broader societal perspective. Resource use data will be collected by questionnaire, capturing information on hospitalisations for exacerbations, COPD-related primary and secondary care attendances and medication usage. Data will also be collected during the trial on resource use implications of the intervention. Information on participant incurred costs, time off work and impact on activities is collected within the economic questionnaire. QALYs will be calculated from EQ-5D-5L responses. Sensitivity analysis will be undertaken and cost-effectiveness planes and cost-effectiveness acceptability curves presented.

Model-based analysis

A decision model will be constructed to extrapolate beyond trial results over five years, with costs and benefits discounted at 3.5%. The model will be the first decision analysis to use GOLD stages ABCD[1] and will consider the ability of the COPDPredict™ supported self-management to impact movements between GOLD ABCD stages, but also the number of exacerbations within those stages.

For peer review only - http://bmjopen.bmj.com/site/about/guidelines.xhtml

Costs associated with delivering COPDPredict™, exacerbations, and routine care will be obtained from the trial. Quality of life scores on each intervention will also be obtained from trial data using EQ-5D-5L. Longer-term costs, quality of life scores, and transitions between stages, and number of exacerbations will be obtained from analysis of ABCD cohorts from within the BLISS[2] study, a prospective cohort study with 3-year follow-up period. The model will estimate cost per QALY gained of COPDPredict™ supported self-management versus standard self-management from a healthcare perspective. Probabilistic sensitivity analysis will be undertaken to simultaneously incorporate all parameter uncertainty. Cost-effectiveness planes and cost-effectiveness acceptability curves will be presented to show the probability the intervention is cost-effective at different cost/QALY thresholds. A value of information analysis will assess the value of further trials to reduce parameter uncertainty and identify parameters which would be most valuable in reducing that uncertainty[29,30].

Qualitative sub-study

In order to frame this study, we draw on Normalisation Process Theory[31], which proposes four constructs essential to effect implementation of new technologies: *coherence* (sense making work to understand the possibilities of a new technology), *cognitive participation* (relational work as new technology begins to be used), *collective action* (operational work enacted to make a technology function in context) and *reflexive monitoring* (the appraisal of work that people do to assess how the new technology affects them and others). Participants will include health care professionals, patients and wider policy stakeholders and will be purposively sampled to maximise diversity. A topic guide developed by drawing on existing literature and theory will focus on attitudes to and practices around self-management of COPD and the use and implementation of digital technologies. Interviews will be audio recorded and transcribed verbatim, before subjecting the data to a two-stage approach to analysis[32]: first, a descriptive framework analysis[33] followed by mapping onto the NPT constructs. We anticipate conducting approximately30 interviews but this will be informed by the data and analysis[34].

For peer review only

Ethics and dissemination:

This study protocol is reported in accordance with the guidance set out in the SPIRIT statement. Predict and Prevent AECOPD has obtained ethical approval in England (19/LO/1939). Upon completion of the trial and publication of results a lay findings summary will be disseminated to trial participants.

Trial Oversight

Trial oversight will be provided by the TMG, an independent DMC and independent TSC. Interim analyses of major outcome measures and safety data will be conducted and provided in strict confidence to the DMC.

Potential Impact

Predict & Prevent AECOPD is a pragmatic, national, multicentre, RCT which aims to provide robust evidence around the clinical and cost effectiveness of COPDPredict™ by analysing the number of patients admitted to hospital in a 12month period post randomisation. It is hoped the outcome of this trial could be used to provide robust evidence to the management of patients with COPD and their exacerbations in the future.

Contributorship statement

All authors have made substantial contributions to the conception or design of the work, or the acquisition, analysis or interpretation of data. DK, AT, HJ, RM, SJ, NG, NP and MS drafted the manuscript and revised it critically for important intellectual content. All authors approved final version and agreed to be accountable for all aspects of the work in ensuring that questions related to the accuracy or integrity of any part of the work are appropriately investigated and resolved.

Competing Interest Statement

The CI of the trial (AT) does not have any relevant direct financial disclosures, nor do members of the trial management group (TMG) who are authors of this paper, with the exception of NP who is a

For peer review only

founder, director and share-holder of NEPeSMO, who developed the intervention. In addition, MS is CI of the overall NIHR-funded project and is a founder, director and share-holder of NEPeSMO. NEPeSMO is a start-up company from the University Hospitals of North Midlands NHS Trust, owns all intellectual property rights of COPDPredict™ and is a project collaborator on the grant.

AT has grants from pharmaceutical companies working in the area of COPD (Chiesi, AstraZeneca) and has conducted advisory work for such (Boehringer, CSL Behring) but not in the area of medical devices or admission prevention. Neither has she worked for, or received monies from, any company working on admission prevention in the last 3 years.

Funding Statement & Disclaimer

This report is independent research funded by the National Institute for Health Research (Invention for Innovation, A Personalised Early Warning Decision Support System with novel Saliva Bio-Profiling to Predict and Prevent Acute Exacerbations of Chronic Obstructive Pulmonary Disease – 'Predict&prevent AECOPD, NIHR200002).

For peer review only

References

1. Global Initiative for Obstructive Lung Disease, [Accessed 13 June 2018]. [Available from: www.goldcopd.com].

2. The battle for breath - the impact of lung disease in the UK. London: British Lung Foundation, 2016.

3. Roberts CM, Stone RA, Lowe D, Pursey NA, Buckingham RJ. Co-morbidities and 90-day outcomes in hospitalized COPD exacerbations. Copd. 2011;8(5):354-61.

4. Stone RA HB, Lowe D, Searle L, Skipper E, Welham S, et al. . National COPD audit programme. COPD: Who cares matters. London, UK: Royal College of Physicians, 2015. Contract No.: eISBN 978-1-86016-559-7.

5. Hurst JR, Vestbo J, Anzueto A, Locantore N, Mullerova H, Tal-Singer R, et al. Susceptibility to exacerbation in chronic obstructive pulmonary disease. N Engl J Med. 2010;363(12):1128-38.

6. Merinopoulou E, Raluy-Callado M, Ramagopalan S, MacLachlan S, Khalid JM. COPD exacerbations by disease severity in England. Int J Chron Obstruct Pulmon Dis. 2016;11:697-709.

7. Stone RA HB, McMillan V, Saleem Khan M, Searle L, Skipper E, et al. COPD: Who cares when it matters most? London: Royal College of Physicians, 2017.

8. Steer J, Gibson GJ, Bourke SC. Longitudinal change in quality of life following hospitalisation for acute exacerbations of COPD. BMJ Open Respir Res. 2015;2(1):e000069.

9. Celli BR, Locantore N, Yates J, Tal-Singer R, Miller BE, Bakke P, et al. Inflammatory biomarkers improve clinical prediction of mortality in chronic obstructive pulmonary disease. Am J Respir Crit Care Med. 2012;185(10):1065-72.

10. Estimating the economic burden of respiratory illness in the UK. London: British Lung Foundation 2016.

For peer review only

11. Rai KK JR, Siebert WS, Sadhra SS, Fitzmaurice DA, Sitch AJ, et al. Birmingham COPD Cohort: a cross-sectional analysis of the factors associated with the likelihood of being in paid employment among people with COPD. Int J Chron Obstruct Pulmon D. Birmingham COPD Cohort: a cross-sectional analysis of the factors associated with the likelihood of being in paid employment among people with COPD. Int J Chron Obstruct Pulmon Dis. 2017;12:233-42. Epub 2017/02/01.

12. McGrath R ASS, Markides K, Hall O, Peterson M. The burden of health conditions for middle-aged and older adults in the United States: disability-adjusted life years. BMC geriatrics. 2019;19(1):100. Epub 2019/04/10.

13. Jo YS, Kim SK, Park SJ, Um S-J, Park Y-B, Jung KS, et al. Longitudinal change of FEV(1) and inspiratory capacity: clinical implication and relevance to exacerbation risk in patients with COPD. Int J Chron Obstruct Pulmon Dis. 2019;14:361-9.

14. Qureshi H SA, Hanania NA. Chronic obstructive pulmonary disease exacerbations: latest evidence and clinical implications. Therapeutic advances in chronic disease. 2014;5(5):212-27. Epub 2014/09/02.

15. Oba, Y., Keeney, E., Ghatehorde, N. and Dias, S., 2018. Dual combination therapy versus long-acting bronchodilators alone for chronic obstructive pulmonary disease (COPD): a systematic review and network meta-analysis. Cochrane Database of Systematic Reviews, [online] 2018(12). Available at: <https://www.cochranelibrary.com/cdsr/doi/10.1002/14651858.CD012620.pub2/full> [Accessed 5 October 2021].

16. Yang, I., Clarke, M., Sim, E. and Fong, K., 2012. Inhaled corticosteroids for stable chronic obstructive pulmonary disease. Cochrane Database of Systematic Reviews, [online] Available at: <http://Inhaled corticosteroids for stable chronic obstructive pulmonary disease> [Accessed 1 September 2021].

17. Ryrso CK, Godtfredsen NS, Kofod LM, Lavesen M, Mogensen L, Tobberup R, et al. Lower mortality after early supervised pulmonary rehabilitation following COPD-exacerbations: a systematic review and meta-analysis. BMC Pulm Med. 2018;18(1):154.

18. Jolly K, Majothi S, Sitch AJ, Heneghan NR, Riley RD, Moore DJ, et al. Self-management of health care behaviors for COPD: a systematic review and meta-analysis. Int J Chron Obstruct Pulmon Dis. 2016;11:305-26.

19. Vijayasaratha K, Stockley RA. Relationship between frequency, length, and treatment outcome of exacerbations to baseline lung function and lung density in alpha-1 antitrypsin-deficient COPD. Int J Chron Obstruct Pulmon Dis. 2012;7:789-96.

20. Patel N, Kinmond K, Jones P, Birks P, Spiteri MA. Validation of COPDPredict™: Unique Combination of Remote Monitoring and Exacerbation Prediction to Support Preventative Management of COPD Exacerbations. International Journal of Chronic Obstructive Pulmonary Disease 2021, 16:1887-1899

For peer review only

21. May CR, Mair F, Finch T, MacFarlane A, Dowrick C, Treweek S, Rapley T, Ballini L, Ong BN, Rogers A, Murray E. Development of a theory of implementation and integration: Normalization Process Theory. Implementation Science. 2009 Dec;4(1):1-9.

22. Nepesmo.com. 2017. NEPeSMO – Predict to Prevent. [online] Available at: <https://nepesmo.com/#:~:text=NEPeSMO%20offers%20a%20smart%20CE,to%20patients%20living%20with%20COPD> [Accessed 16 June 2021].

23. Pinnock H, Hanley J, McCloughan L, Todd A, Krishan A, Lewis S, et al. Effectiveness of telemonitoring integrated into existing clinical services on hospital admission for exacerbation of chronic obstructive pulmonary disease: researcher blind, multicentre, randomised controlled trial. Bmj. 2013;347:f6070

24. Sandeep K Gupta. Intention-to-treat concept: A Review. Perspect Clin Res 2011:2(3);109-112

25. White IR, Royston P, Wood AM. Multiple imputation using chained equations. Stats in Medicine 2011, (30) 377-399.

26. Vrieze A, de Greef MH, Wijkstra PJ, Wempe JB. Low bone mineral density in COPD patients related to worse lung function, low weight and decreased fat-free mass. Osteoporos Int. 2007;18(9):1197-202.

27. Patel AR, Kowlessar BS, Donaldson GC, Mackay AJ, Singh R, George SN, et al. Cardiovascular risk, myocardial injury, and exacerbations of chronic obstructive pulmonary disease. Am J Respir Crit Care Med. 2013;188(9):1091-9.

28. Miller J, Edwards LD, Agusti A, Bakke P, Calverley PM, Celli B, et al. Comorbidity, systemic inflammation and outcomes in the ECLIPSE cohort. Respir Med. 2013;107(9):1376-84.

29. Global Initiative for Obstructive Lung Disease COPD Diagnosis and Management At-A-Glance Desk Reference 2017 [online] https://goldcopd.org/archived-reports/ Accessed 03/09/21

30. Adab P, Fitzmaurice D, Dickens, A et al. The Birmingham Chronic Obstructive Pulmonary Disease (COPD) Cohort Study. Int. J. Epidemiology 2017;46(1); 23. https://doi.org/10.1093/ije/dyv350

31. May C, Finch T. Implementing, Embedding, and Integrating Practices: An Outline of Normalization Process Theory. Sociology. 2009;43(3):535-54.

32. Macfarlane A, O'Reilly-de Brún M. Using a theory-driven conceptual framework in qualitative health research. Qual Health Res. 2012;22(5):607-18.

33. Ritchie J, Spencer L. Qualitative data analysis for applied policy research. In: Bryman A, Burgess RG, editors. London: Routledge; 1994.

For peer review only

34. O'Cathain A, Hoddinott P, Lewin S, Thomas KJ, Young B, Adamson J, et al. Maximising the impact of qualitative research in feasibility studies for randomised controlled trials: guidance for researchers. Pilot and Feasibility Studies. 2015;1(1):32.

35. Gale, Nicola K., Gemma Heath, Elaine Cameron, Sabina Rashid, and Sabi Redwood. "Using the framework method for the analysis of qualitative data in multi-disciplinary health research." BMC medical research methodology 13, no. 1 (2013): 1-8.

36. Agusti A, Edwards LD, Rennard SI, MacNee W, Tal-Singer R, Miller BE, et al. Persistent systemic inflammation is associated with poor clinical outcomes in COPD: a novel phenotype. PLoS One. 2012;7(5):e37483.

37. Labat C, Temmar M, Nagy E, Bean K, Brink C, Benetos A, et al. Inflammatory mediators in saliva associated with arterial stiffness and subclinical atherosclerosis. J Hypertens. 2013;31(11):2251-8; discussion 8.

For peer review only

Figure Legends

Figure 1: Flow diagram of the Predict & Prevent trial

Table Legends

Table 1: Schedule of assessments

For peer review only - http://bmjopen.bmj.com/site/about/guidelines.xhtml

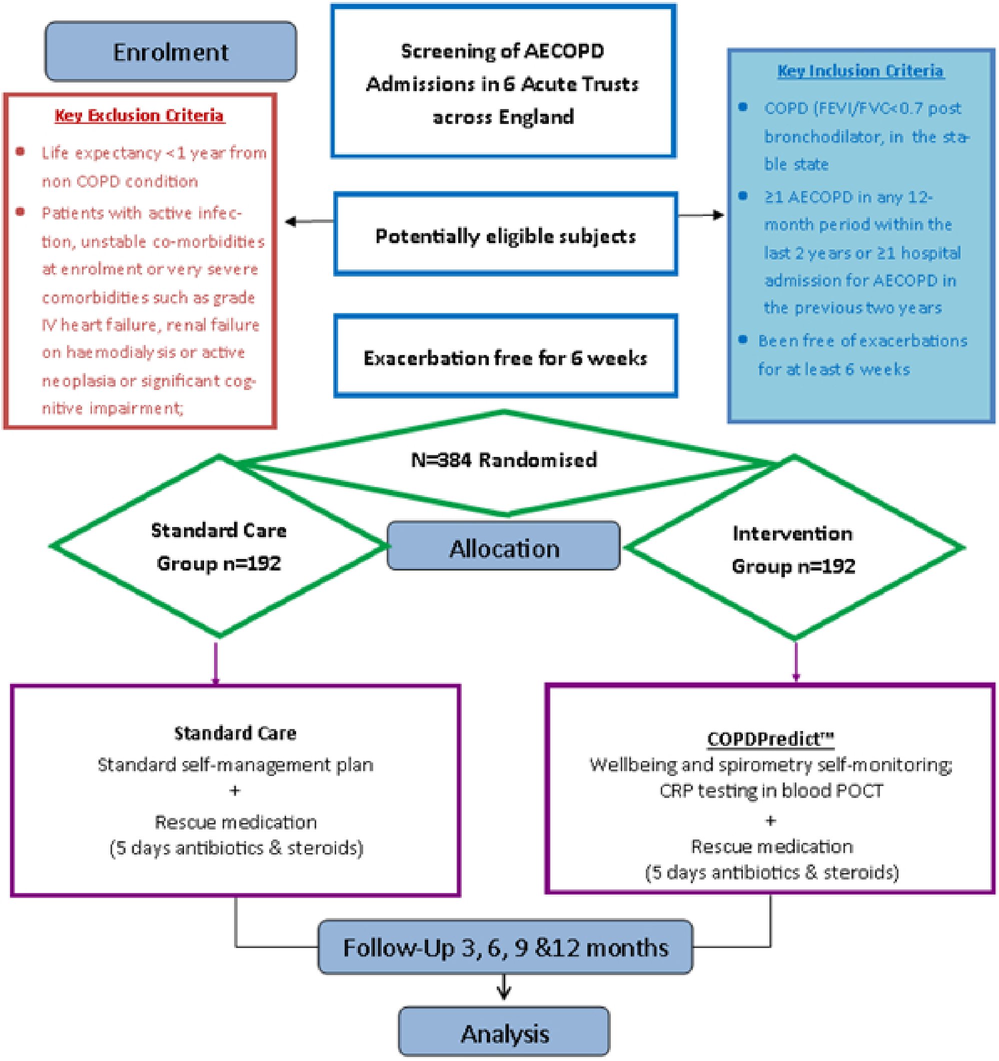

Figure 1: Flow diagram of the Predict & Prevent trial

266x297mm (57 x 57 DPI)

BMJ Open

A phase III, 2 arm, multi-centre, open label, parallel-group randomised designed clinical investigation of the use of a personalised early warning decision support system to predict and prevent acute exacerbations of Chronic Obstructive Pulmonary Disease - 'Predict & Prevent AECOPD' -Study Protocol

Journal:	*BMJ Open*
Manuscript ID	bmjopen-2022-061050.R1
Article Type:	Protocol
Date Submitted by the Author:	28-Jul-2022
Complete List of Authors:	Kaur, Dalbir; University of Birmingham,<br>Mehta, Rajnikant ; University of birmingham<br>Jarrett, Hugh; University of Birmingham<br>Jowett, Sue; University of Birmingham, Health Economics Unit<br>Gale, Nicola K.; University of Birmingham<br>Turner, Alice; University of Birmingham, Institute of Inflammation & Aging; Heart of England NHS Foundation Trust<br>Spiteri, Monica; University Hospitals of North Midlands NHS Trust<br>Patel, Neil; NEPESMO
**Primary Subject Heading**:	Respiratory medicine
Secondary Subject Heading:	Smoking and tobacco
Keywords:	RESPIRATORY MEDICINE (see Thoracic Medicine), Chronic airways disease < THORACIC MEDICINE, Thoracic medicine < INTERNAL MEDICINE

SCHOLARONE™
Manuscripts

A phase III, 2 arm, multi-centre, open label, parallel-group randomised designed clinical investigation of the use of a personalised early warning decision support system to predict and prevent acute exacerbations of Chronic Obstructive Pulmonary Disease - 'Predict & Prevent AECOPD' -Study Protocol

D Kaur, R Mehta, H Jarrett, S Jowett, N Gale, AM Turner, MA Spiteri, N Patel

Dalbir Kaur[1]
Email: dalbir.kaur@warwick.ac.uk

Rajnikant Mehta[2]
Email: R.L.Mehta@bham.ac.uk

Hugh Jarrett[3]
Email: h.jarrett@bham.ac.uk

Sue Jowett[4]
Email: S.JOWETT@bham.ac.uk

Nicola Gale[5]
Email: N.Gale@bham.ac.uk

Alice M Turner[6]
Email: A.M.Turner@bham.ac.uk

Monica A Spiteri[7]
Email: Monica.Spiteri@uhnm.nhs.uk

Neil Patel[8]
Email: neil.patel@nepesmo.com

[1]Senior Trials Manager, Birmingham Clinical Trials Unit (BCTU), Institute of Applied Health Research, College of Medical and Dental Sciences, University of Birmingham, Edgbaston, Birmingham, B15 2TT

[2]Senior Medical Statistician, Birmingham Clinical Trials Unit (BCTU), Institute of Applied Health Research, College of Medical and Dental Sciences, University of Birmingham, Edgbaston, Birmingham, B15 2TT

[3]Trials Management Team Leader, Birmingham Clinical Trials Unit (BCTU), Institute of Applied Health Research, College of Medical and Dental Sciences, University of Birmingham, Edgbaston, Birmingham, B15 2TT

[4]Professor of Health Economics, Health Economics Unit, Institute of Applied Health Research, College of Medical and Dental Sciences, University of Birmingham, Edgbaston, Birmingham, B15 2TT

[5]Professor of Health Policy and Sociology, Health Services Management Centre, School of Social Policy Director of Postgraduate Research, College of Social Sciences, University of Birmingham, Edgbaston, Birmingham, B15 2TT

[6] Professor of Respiratory Medicine and Honorary Consultant Respiratory Physician, Institute for Applied Health Research, University of Birmingham, Birmingham, UK, B15 2TT

For peer review only

[7]Director of Respiratory Research, Academic Research Unit, Courtyard Annexe - C Block, Royal Stoke University Hospital, University Hospitals of North Midlands NHS Trust, Newcastle Road, Staffordshire, UK, ST4 6QG

[8]Chief Medical Officer, NEPESMO Ltd, 76 King Street, Manchester, M2 4NH, United Kingdom
Word count: 3487 (excluding title page, abstract, table, legend and references)

Abstract

Introduction

With 65 million cases globally, chronic obstructive pulmonary disease (COPD) is the 4th leading cause of death and imposes a heavy burden on patients' lives and healthcare resources worldwide. Around half of all patients with COPD have frequent (≥2 per year) AECOPD[5]. Rapid readmissions are also common. Exacerbations impact significantly on COPD outcomes, causing significant lung function decline. Prompt exacerbation management optimises recovery and delays the time to the next acute episode.

Methods/analysis

The Predict & Prevent AECOPD trial is a phase III, 2 arm, multi-centre, open label, parallel-group individually randomised clinical trial investigating the use of a personalised early warning decision support system (COPDPredict™) to predict and prevent acute exacerbations of COPD. We aim to recruit 384 participants and randomise each individual in a 1:1 ratio to either standard self-management plans (SSMP) with Rescue Medication (RM) (control arm) or COPDPredict™ with RM (intervention arm)

The trial will inform the future standard of care regarding management of exacerbations in COPD patients. The main outcome measure is to provide further validation, as compared to usual care, for the clinical effectiveness of COPDPredict™ to help guide and support COPD patients and their respective clinical teams in identifying exacerbations early, with an aim to reduce the total number of AECOPD-induced hospital admissions in the 12 months following each patient's randomisation.

Ethics and Dissemination

This study protocol is reported in accordance with the guidance set out in the SPIRIT statement. Predict and Prevent AECOPD has obtained ethical approval in England (19/LO/1939). Upon completion of the trial and publication of results a lay findings summary will be disseminated to trial participants.

Trials Registration: Clinical trials.gov NCT04136418

Keywords: Respiratory, COPD, Exacerbations, Symptom Control Markers, COPDPredict™

NIHR Statement

This work is supported by the National Institute for Health Research (NIHR) under its Invention for Innovation (i4i) Programme (Grant Reference Number NIHR200002). The views expressed are those of the author(s) and not necessarily those of the NHS, the NIHR or the Department of Health.

Strengths and Limitations

- This study will be conducted as a phase 3 randomised controlled trial concerning an intervention for the early warning of COPD exacerbations, COPDPredict™, in patients with COPD. It will provide clinicians with a possible alternative for detecting COPD exacerbations early and actioning timely treatment changes to prevent unnecessary hospitalisation.
- It has the potential to better the patient's quality of life if the burden of AECOPD is reduced. The secondary outcomes will enable conclusions around cost saving for the NHS.
- The study is limited to detecting effects on hospitalisations, and it is possible important changes in other aspects of COPD could be missed, as not powered to detect them.

For peer review only

Background

With 65 million cases globally, chronic obstructive pulmonary disease (COPD) is the 4th leading cause of death and imposes a heavy burden on patients' lives and healthcare resources worldwide[1]. UK death rates are currently double the European Union (EU) average with over 30,000 people dying yearly and annual COPD-related NHS direct costs exceeding £800m[2]. Patients can have acute exacerbations of their COPD (AECOPD), which reduce quality of life, and lead to 140,000 emergency hospital admissions a year in the UK[3]. Indeed, AECOPD remain the 2nd commonest cause of emergency hospital admissions and 1 in 3 of these patients (sometimes more) will be readmitted within 3 months[4,5]. Hospitalisation itself carries a poor prognosis with an increased mortality risk.

For peer review only

Around half of all patients with COPD have frequent (≥2 per year) AECOPD[6,7]. Rapid readmissions are also common – the national COPD audit has shown that 43% of patients with COPD who were admitted are back in hospital within 90 days[8], and up to 71% by 12 months[9]. Exacerbations impact significantly on COPD outcomes, causing significant lung function decline[10]. Prompt exacerbation management optimises recovery and delays the time to the next acute episode[11], with NICE COPD guidelines highlighting a time window (prodrome) between an initial exacerbation's symptoms/signs and subsequent hospitalisation. Within this prodrome there is an opportunity to intervene. Current practice for COPD patients is that they are encouraged to recognise AECOPD, via standard self-management plans (SSMPs), and treat, using rescue medication (RM), but in many cases because of day-to-day variability in symptoms, the start of an exacerbation goes unrecognised and untreated. This inability/uncertainty to recognise and treat exacerbations in their early phase can lead to hospital admissions, which may drive long term decline.

Pharmacological and non-pharmacological treatments are used to reduce COPD exacerbations[12,13], and improve care, such as pulmonary rehabilitation (PR[14]), azithromycin[15], inhaled bronchodilators and inhaled corticosteroids. Infection control measures during the pandemic reduced spread of

other respiratory viruses, which in turn was associated with a 43% fall in admissions, with benefits maintained post-lockdown[16] though this is not currently part of international COPD guidelines[17, 18]. SSMPs help patients but have not really shown significant impact on A&E visits or hospital admissions as demonstrated by a systematic review of self-management strategies for COPD[19]. Intuitively early recognition and treatment of AECOPD would reduce exacerbation severity and duration, and improve prognosis; evidence for this is limited but supportive[20,21].

This study looks to address the problem by personalising and thus optimising effectiveness of AECOPD management using the CE marked and MHRA registered solution, COPDPredict™[22].

For peer review only

Methods

Patient and public involvement

Throughout the design and delivery of the trial public and participant involvement (PPI) has been accessed. In particular independent members on the trial steering committee provide oversight and contributions to the trial, including design and standardisation of trial-specific SSMP. PPI involvement has also extended to the qualitative research where the topic guide was developed in conjunction with respiratory participant groups.

Trial Design

The Predict & Prevent AECOPD trial is a phase III, 2 arm, multi-centre, open label, parallel-group individually randomised clinical trial investigating the use of a personalised early warning decision support system (COPDPredict™) to predict and prevent acute exacerbations of COPD. We aim to recruit 384 participants from NHS hospitals in the UK and randomise each individual in a 1:1 ratio to either SSMP with RM (control arm) or COPDPredict™ with RM (intervention arm) (*Figure 1*). The start date for the trial was 30th January 2019 and the end date for the trial is 30th March 2023.

The main outcome measure is to provide further validation, as compared to usual care, for the clinical effectiveness of COPDPredict™ to help guide and support COPD patients and their respective clinical teams in identifying exacerbations early, with an aim to reduce the total number of AECOPD-

induced hospital admissions in the 12 months following each patient's randomisation. The trial includes a health economic analysis examining health-related quality of life (HRQoL) using the validated questionnaire EQ-5D-5L and healthcare utilisation (as determined by a questionnaire that inquires as to hospitalisations, GP attendances and medication usage). A qualitative sub-study will assess both the end user experience and the healthcare provider experience, using Normalization Process Theory to understand the implementation and integration of the new technology[23].

Eligibility Criteria

Inclusion Criteria

- Clinically diagnosed COPD, confirmed by post-bronchodilator spirometry and defined as Post bronchodilator Forced expiratory volume/Forced vital capacity (FEV_1/FVC) <0.7 and <lower limit of normal, in the stable state
- ≥1 AECOPD in any 12-month period within the last 2 years or ≥1 hospital admission for AECOPD in the previous two years.
- Exacerbation free for at least 6 weeks
- An age of at least 18 years
- Willing and able to comply with data collection to 12 months from randomisation
- Ability to consent
- Ability to use intervention as judged by the investigator at screening, upon demonstration of the system to the patient

Exclusion Criteria

- Life expectancy <12 months -Assessment the individual investigator
- Co-enrolment into any clinical trials of investigative medicinal product (CTIMPs)
- Patients with selected co-morbidities, where these could impair use of the intervention (eg dementia)

For peer review only

Outcomes

Primary Outcome

Number of hospital admissions at 12 months post randomisation where the primary reason for admission is AECOPD. This will be obtained from participant testimony and from centrally held (HES) records. Data from the 2 sources will be cross-referenced to remove double-counting. The total number of admissions will be the sum of unique admissions from the 2 sources of data

Secondary Outcomes

Clinical Outcomes

Over a 12-month period, following randomisation;

Total in participant days, Number of visits to A&E, Number of participant defined exacerbations, Appropriate action on symptoms of AECOPD, as shown by the app, or participant testimony, Health-related quality of life (CAT) and, FEV_1 at 12 months post randomisation (from spirometry obtained during study visit)

Economic Outcomes

Health-related quality of life at baseline and 3, 6, 9 and 12-months post randomisation (EQ-5D-5L)

Healthcare utilisation at 3, 6, 9 and 12-months post randomisation, as determined by a questionnaire that inquires on hospitalisations, GP attendances and medication usage

Qualitative sub-study

End user experience and provider experience

Control Arm

A standard self-management plan (SSMP) alongside use of rescue medication (RM) containing 5 days of antibiotic and steroid treatment.

For peer review only

Intervention

COPDPredict™, consists of a patient-facing App (iOS/Android) and clinician-facing dashboard[24]. The Early Warning Decision Support System uses longitudinal remote monitoring of relevant subjective and objective data (symptoms, spirometry, biomarkers) in real-time via the App which is connected to CE-marked Bluetooth-enabled sensor peripherals. These data are used to construct COPD-relevant individual profiles such that artificial intelligence artificial intelligence (AI)-driven algorithms can then identify changes in health status to provide timely individualised alerts to patients and clinicians and sign-posting to action plans for patients.

COPDPredict™ also provides information around COPD self-management, pulmonary rehabilitation, inhaler technique and utilises gamification to help with adherence. Patients have full access to their results and can also directly message their clinical team. COPDPredict™ may signpost a participant to a self-management action plan for exacerbation management but clinical team supervision and oversight will remain throughout, as per usual care.

The clinician facing dashboard allows for "real-time" case management with the ability to remotely monitor patients and facilitate interaction. Clinicians can choose to escalate treatments based on the results being transmitted by the patients. A crucial dimension to this advanced system is that blood biomarker measurements which inform an algorithm are incorporated to enhance accuracy. Blood-based point of care (POC) analysers are used with COPDPredict™ and these POC analysers have previously been validated against laboratory CRP measures.

Recruitment and Consent

All patients must provide fully informed consent. A Participant Information Sheet (PIS) which explains the different ways in which the participant may enter the trial is provided to participants to facilitate this process. Investigators or delegate(s) adequately explain the aim, trial intervention, anticipated benefits and potential hazards of taking part in the trial to the participant. They also stress that participation is voluntary and that the participant is free to refuse to take part and may

For peer review only

withdraw from the trial at any time. Any trial specific procedures are completed after the participant has given informed consent. At each visit the participant's willingness to continue in the trial is ascertained and documented in the medical notes. Where new information becomes available which may affect the participants' decision to continue, participants will be given time to consider and if happy to continue will be re-consented. Re-consent will be documented in the medical notes.

Randomisation

After informed consent has been obtained and full participant eligibility confirmed (including exacerbation free for 6 weeks), the participant can be randomised into the trial. Randomisation CRFs are provided to investigators and may be used to collate the necessary information prior to randomisation. Participants are randomised by computer/telephone at the level of the individual in a 1:1 ratio to either SSMP with RM (control arm) or COPDPredict™ with RM (intervention arm).

A minimisation algorithm will be used within the online randomisation system to ensure balance in the treatment allocation over centre, age (<60, ≥60 years) and severity of disease as per Global Initiative for Obstructive Lung Disease (GOLD)[1].

The participants are randomised to one of the following:

Control Arm

A standard self-management plan (SSMP) alongside use of rescue medication (RM) containing 5 days of antibiotic and steroid treatment.

Intervention arm

Supported self-management using the COPDPredict™ App, involving personalised alerts to both patients and the clinical care team.

A 'random element' will be included in the minimisation algorithm, so that each participant has a probability, of being randomised to the opposite treatment that they would have otherwise

For peer review only

received. Once randomised patients will also be asked to complete baseline quality of life booklet which contains both the EQ-5D-5L and CAT questionnaire.

For peer review only

Assessment schedule

Visit	Hospital Discharge/ Initial appointment	Personal Baseline Day 1 after 6 weeks exacerbation free	Personal Baseline Day 7 (±2 days)	End of Personal Baseline Period	Approximately 4 times during any exacerbation (home visit or clinic)	Weekly until Week 52	Week 13 Telephone follow Up +/-1 week	Week 26 Hospital/ Telephone follow Up +/-1 week	Week 39 Telephone follow Up +/-1 week	Week 52 Telephone /Hospital follow Up +/- 2 weeks	Patients daily wellbeing completion post two-week bassline period
Eligibility check	X										
Valid informed consent	X										
Randomisation		X									
Height and Weight	X									X	
Concomitant medication	X	X		X				X	X	X	
Baseline medical history taken$^{\alpha}$	X										
Request to GP for rescue medication	X						(X)	(X)	(X)		
Assessment of (S)AEs	X				(X)		X	X	X	X	

For peer review only - http://bmjopen.bmj.com/site/about/guidelines.xhtml

Questionnaires (QoL and health utilisation)	X						X	X	X	X	
Spirometry	X							X		X	
Intervention only below this line					Intervention only below this line						
Provision of equipment & participant training		X									
Spirometry	X	X	X		(X)	X					
Symptom control markers	X	X	X	X	(X)	X	X	X	X	X	
Point of care blood test		X		X	(X)						
Clinical histories		X		X			X	X	X	X	
Wellbeing (App)											X

(X) denotes only when required. If rescue medication has not been used, fresh prescription requests will not be necessary

During the baseline period and any subsequent exacerbations patients will be entering well-being data on the App which will be related to lifestyle choices and the end-user experience. This data will not form part of the formal analysis of quality of life.

Table 1: Schedule of assessments

For peer review only

Statistical Analysis

Sample size

The justification of the sample size is based on previous evidence[25] that had shown a mean estimate of 2.5 COPD admissions in the previous year in the control group. To detect a difference of 1 admission in the mean number of admissions between groups using the standard methods of difference between means and assuming standard deviation of 2.6 with 90% power and a type I error rate of 5% (two-sided), 144 participants per group will need to be randomised, 288 in total. Assuming and adjusting for a 25% loss to follow-up/ drop-out rate, 384 participants will need to be recruited.

Analysis of Outcome Measures

The primary comparison groups will be composed of those randomised to the use of COPDPredict™ system and those randomised to the standard self-management plan (SSMP). All analyses will be based on the intention to treat principle[26], with all patients analysed in the treatment groups to which they were allocated irrespective of compliance with the randomised allocated treatment, and all patients will be included in the analyses. For all outcomes, summary statistics (e.g. mean differences, relative risks) will be reported and 95% confidence intervals will be constructed where appropriate. A two-sided p-value of <0.05 will be considered statistically significant.

Primary Outcome Measure

The primary analysis for this study will be to compare rates of hospital admission between the treatment groups (COPDPredict™ versus Usual care) over 12 months, following randomisation, where the primary reason for admission is an AECOPD event. These hospital admissions per person will be analysed using Poisson regression models adjusting for treatment group and minimisation variables. If there is over dispersion a negative binomial regression model adjusting for treatment group and the same minimisation variables will be taken into consideration. Point estimates

For peer review only

(incidence rates) will be provided and accompanied with 95% confidence intervals and respective p-values.

Secondary Outcome Measures

Recorded observations taken over a 12-month period, following randomisation:

Total in-hospital days and FEV_1 – These variables will be summarised using basic descriptive statistics (Mean, SD)). We may also consider a mixed linear regression model, to estimate differences between the intervention group supported with 95% CI adjusting for baseline variables and minimisation variables (centre being a random effects variable).

Health-related quality of life questionnaires - EQ5D-5L and CAT will be converted into scores and analysed using a mixed linear regression model, adjusting for the intervention group, baseline and minimisation variables as before.

Number of A&E visits, participant defined exacerbations and healthcare utilisation - Where the participant experienced an episode, the data during the 12 months from randomisation will be analysed using mixed effects log-binomial regression techniques with presentation of relative risk and 95% confidence intervals. Furthermore, sensitivity analysis based on patients' experience of multiple visits to A&E and participant defined exacerbation will be calculated using Poisson regression techniques and relative risk supported with 95% CI.

Symptom control marker association to clinical decision - The diagnostic accuracy of COPDPredict™ and Usual care will be evaluated by calculating sensitivity, specificity, positive and negative predictive values, and area under curve (AUC)/receiver operating characteristics (ROC) together with 95% CI.

Subgroup analyses

Subgroup analyses will be limited to the same variables used in the minimisation algorithm, apart from centre. Tests for statistical heterogeneity (e.g. by including the treatment group by subgroup

For peer review only

interaction parameter in the statistical model) will be performed prior to any examination of effect estimate within subgroups. The results of subgroup analyses will be treated with caution and will be used for the purposes of hypothesis generation only.

Missing data and sensitivity analyses

Participants with missing primary outcome data will not be included in the primary analysis in the first instance. This presents a risk of bias, and sensitivity analyses will be undertaken to assess the possible impact. This will consist of simulating the missing response using a multiple imputation approach[27]. Parameters used to simulate the missing response will include the minimisation variables, intervention group and previous response. Full details are included in the statistical analysis plan (SAP).

Adverse events and serious adverse events

Patients with COPD can have high disease burden, such that the trial population will be older, with co-morbidities such as osteoporosis[28], cardiovascular disease or raised cardiovascular risk[29], along with associated complications and symptoms including abnormal lab results. A relatively high number of adverse events are anticipated as a result of the patients' existing past medical history[30]. However, this study is examining the role of increased participant surveillance and therefore few, if any, foreseeable risks of direct harm associated with the study intervention are anticipated. AE reporting will therefore be limited to those events which are required for trial monitoring or outcome assessment.

All events which meet the definition of serious must be recorded in the participant notes, but for trial purposes these following events do not require reporting on the SAE Form. Such events are "safety reporting exempt".

• Planned hospitalisation for a pre-existing condition, or a procedure required by the trial protocol without serious deterioration in health, is not considered a serious adverse event.

For peer review only - http://bmjopen.bmj.com/site/about/guidelines.xhtml

• An overnight stay in hospital that is due to transportation, organisational or accommodation problems, and without medical background SAES requiring expedited reporting

Any other events that fulfil the usual definition of an SAE will be reported immediately.

Any death occurring during the Trial Protocol defined follow up period (12 months) must be reported as an SAE within 24 hours of the local investigator becoming aware of the event.

Health economics

Within -trial analysis

The economic evaluation alongside the trial will take the form of an incremental cost-effectiveness analysis to estimate cost per hospital admission avoided and incremental cost-utility analysis to estimate cost per quality adjusted life year (QALY) gained. Base-case analyses will be from a healthcare perspective over 12 months follow-up using individual participant level data on costs and outcomes from the clinical trial. Additional analyses will be undertaken from a broader societal perspective. Resource use data will be collected by questionnaire, capturing information on hospitalisations for exacerbations, COPD-related primary and secondary care attendances and medication usage. Data will also be collected during the trial on resource use implications of the intervention. Information on participant incurred costs, time off work and impact on activities is collected within the economic questionnaire. QALYs will be calculated from EQ-5D-5L responses. Sensitivity analysis will be undertaken and cost-effectiveness planes and cost-effectiveness acceptability curves presented.

Model-based analysis

A decision model will be constructed to extrapolate beyond trial results over five years, with costs and benefits discounted at 3.5%. The model will be the first decision analysis to use GOLD stages ABCD[1] and will consider the ability of the COPDPredict™ supported self-management to impact movements between GOLD ABCD stages, but also the number of exacerbations within those stages.

For peer review only

Costs associated with delivering COPDPredict™, exacerbations, and routine care will be obtained from the trial. Quality of life scores on each intervention will also be obtained from trial data using EQ-5D-5L. Longer-term costs, quality of life scores, and transitions between stages, and number of exacerbations will be obtained from analysis of ABCD cohorts from within the BLISS[2] study, a prospective cohort study with 3-year follow-up period. The model will estimate cost per QALY gained of COPDPredict™ supported self-management versus standard self-management from a healthcare perspective. Probabilistic sensitivity analysis will be undertaken to simultaneously incorporate all parameter uncertainty. Cost-effectiveness planes and cost-effectiveness acceptability curves will be presented to show the probability the intervention is cost-effective at different cost/QALY thresholds. A value of information analysis will assess the value of further trials to reduce parameter uncertainty and identify parameters which would be most valuable in reducing that uncertainty[31].

Qualitative sub-study

In order to frame this study, we draw on Normalisation Process Theory[32], which proposes four constructs essential to effect implementation of new technologies: *coherence* (sense making work to understand the possibilities of a new technology), *cognitive participation* (relational work as new technology begins to be used), *collective action* (operational work enacted to make a technology function in context) and *reflexive monitoring* (the appraisal of work that people do to assess how the new technology affects them and others). Participants will include health care professionals, patients and wider policy stakeholders and will be purposively sampled to maximise diversity. A topic guide developed by drawing on existing literature and theory will focus on attitudes to and practices around self-management of COPD and the use and implementation of digital technologies. Interviews will be audio recorded and transcribed verbatim, before subjecting the data to a two-stage approach to analysis[336]: first, a descriptive framework analysis[34] followed by mapping onto the NPT constructs. We anticipate conducting approximately30 interviews but this will be informed by the data and analysis[35].

For peer review only - http://bmjopen.bmj.com/site/about/guidelines.xhtml

Ethics and dissemination:

This study protocol is reported in accordance with the guidance set out in the SPIRIT statement. Predict and Prevent AECOPD has obtained ethical approval in England (19/LO/1939). Upon completion of the trial and publication of results a lay findings summary will be disseminated to trial participants.

Trial Oversight

Trial oversight will be provided by the TMG, an independent DMC and independent TSC. Interim analyses of major outcome measures and safety data will be conducted and provided in strict confidence to the DMC.

Potential Impact

Predict & Prevent AECOPD is a pragmatic, national, multicentre, RCT which aims to provide robust evidence around the clinical and cost effectiveness of COPDPredict™ by analysing the number of patients admitted to hospital in a 12month period post randomisation. It is hoped the outcome of this trial could be used to provide robust evidence to the management of patients with COPD and their exacerbations in the future.

Contributorship statement

All authors have made substantial contributions to the conception or design of the work, or the acquisition, analysis or interpretation of data. DK, AT, HJ, RM, SJ, NG, NP and MS drafted the manuscript and revised it critically for important intellectual content. All authors approved final version and agreed to be accountable for all aspects of the work in ensuring that questions related to the accuracy or integrity of any part of the work are appropriately investigated and resolved.

Competing Interest Statement

The CI of the trial (AT) does not have any relevant direct financial disclosures, nor do members of the trial management group (TMG) who are authors of this paper, with the exception of NP who is a

For peer review only

founder, director and share-holder of NEPeSMO, who developed the intervention. In addition, MS is CI of the overall NIHR-funded project and is a founder, director and share-holder of NEPeSMO. NEPeSMO is a start-up company from the University Hospitals of North Midlands NHS Trust, owns all intellectual property rights of COPDPredict™ and is a project collaborator on the grant.

AT has grants from pharmaceutical companies working in the area of COPD (Chiesi, AstraZeneca) and has conducted advisory work for such (Boehringer, CSL Behring) but not in the area of medical devices or admission prevention. Neither has she worked for, or received monies from, any company working on admission prevention in the last 3 years.

Funding Statement & Disclaimer

This report is independent research funded by the National Institute for Health Research (Invention for Innovation, A Personalised Early Warning Decision Support System with novel Saliva Bio-Profiling to Predict and Prevent Acute Exacerbations of Chronic Obstructive Pulmonary Disease – 'Predict&prevent AECOPD, NIHR200002).

For peer review only

References

1. Global Initiative for Obstructive Lung Disease, [Accessed 13 June 2018]. [Available from: www.goldcopd.com].

2. Estimating the economic burden of respiratory illness in the UK. London: British Lung Foundation 2016.

3. The battle for breath - the impact of lung disease in the UK. London: British Lung Foundation, 2016.

4. Roberts CM, Stone RA, Lowe D, Pursey NA, Buckingham RJ. Co-morbidities and 90-day outcomes in hospitalized COPD exacerbations. Copd. 2011;8(5):354-61.

5. Stone RA HB, Lowe D, Searle L, Skipper E, Welham S, et al. . National COPD audit programme. COPD: Who cares matters. London, UK: Royal College of Physicians, 2015. Contract No.: eISBN 978-1-86016-559-7.

6. Hurst JR, Vestbo J, Anzueto A, Locantore N, Mullerova H, Tal-Singer R, et al. Susceptibility to exacerbation in chronic obstructive pulmonary disease. N Engl J Med. 2010;363(12):1128-38.

7. Merinopoulou E, Raluy-Callado M, Ramagopalan S, MacLachlan S, Khalid JM. COPD exacerbations by disease severity in England. Int J Chron Obstruct Pulmon Dis. 2016;11:697-709.

8. Stone RA HB, McMillan V, Saleem Khan M, Searle L, Skipper E, et al. COPD: Who cares when it matters most? London: Royal College of Physicians, 2017.

9. Steer J, Gibson GJ, Bourke SC. Longitudinal change in quality of life following hospitalisation for acute exacerbations of COPD. BMJ Open Respir Res. 2015;2(1):e000069.

10. Jo YS, Kim SK, Park SJ, Um S-J, Park Y-B, Jung KS, et al. Longitudinal change of FEV(1) and inspiratory capacity: clinical implication and relevance to exacerbation risk in patients with COPD. Int J Chron Obstruct Pulmon Dis. 2019;14:361-9.

For peer review only

11. Qureshi H SA, Hanania NA. Chronic obstructive pulmonary disease exacerbations: latest evidence and clinical implications. Therapeutic advances in chronic disease. 2014;5(5):212-27. Epub 2014/09/02.

12. Oba, Y., Keeney, E., Ghatehorde, N. and Dias, S., 2018. Dual combination therapy versus long-acting bronchodilators alone for chronic obstructive pulmonary disease (COPD): a systematic review and network meta-analysis. Cochrane Database of Systematic Reviews, [online] 2018(12). Available at: <https://www.cochranelibrary.com/cdsr/doi/10.1002/14651858.CD012620.pub2/full> [Accessed 5 October 2021].

13. Yang, I., Clarke, M., Sim, E. and Fong, K., 2012. Inhaled corticosteroids for stable chronic obstructive pulmonary disease. Cochrane Database of Systematic Reviews, [online] Available at: <http://Inhaled corticosteroids for stable chronic obstructive pulmonary disease> [Accessed 1 September 2021].

14. Ryrso CK, Godtfredsen NS, Kofod LM, Lavesen M, Mogensen L, Tobberup R, et al. Lower mortality after early supervised pulmonary rehabilitation following COPD-exacerbations: a systematic review and meta-analysis. BMC Pulm Med. 2018;18(1):154.

15. Ni, W., Shao, X., Cai, X., Wei, C., Cui, J., Wang, R. and Liu, Y., 2015. Prophylactic Use of Macrolide Antibiotics for the Prevention of Chronic Obstructive Pulmonary Disease Exacerbation: A Meta-Analysis. PLOS ONE, 10(3), p.e0121257.

16. Lawless, M., Burgess, M. and Bourke, S., 2022. Impact of COVID-19 on Hospital Admissions for COPD Exacerbation: Lessons for Future Care. Medicina, 58(1), p.66.

17. Nice.org.uk. 2018. Overview | Chronic obstructive pulmonary disease in over 16s: diagnosis and management | Guidance | NICE. [online] Available at: <https://www.nice.org.uk/guidance/ng115> [Accessed 23 July 2022].

18. Global Initiative for Chronic Obstructive Lung Disease - GOLD. 2022. 2022 GOLD Reports - Global Initiative for Chronic Obstructive Lung Disease - GOLD. [online] Available at: <https://goldcopd.org/2022-gold-reports-2/> [Accessed 23 July 2022].

19. Jolly K, Majothi S, Sitch AJ, Heneghan NR, Riley RD, Moore DJ, et al. Self-management of health care behaviors for COPD: a systematic review and meta-analysis. Int J Chron Obstruct Pulmon Dis. 2016;11:305-26.

20. Vijayasaratha K, Stockley RA. Relationship between frequency, length, and treatment outcome of exacerbations to baseline lung function and lung density in alpha-1 antitrypsin-deficient COPD. Int J Chron Obstruct Pulmon Dis. 2012;7:789-96.

21. Celli BR, Locantore N, Yates J, Tal-Singer R, Miller BE, Bakke P, et al. Inflammatory biomarkers improve clinical prediction of mortality in chronic obstructive pulmonary disease. Am J Respir Crit Care Med. 2012;185(10):1065-72.

For peer review only

For peer review only - http://bmjopen.bmj.com/site/about/guidelines.xhtml

22. Patel N, Kinmond K, Jones P, Birks P, Spiteri MA. Validation of COPDPredict™: Unique Combination of Remote Monitoring and Exacerbation Prediction to Support Preventative Management of COPD Exacerbations. International Journal of Chronic Obstructive Pulmonary Disease 2021, 16:1887-1899

23. May CR, Mair F, Finch T, MacFarlane A, Dowrick C, Treweek S, Rapley T, Ballini L, Ong BN, Rogers A, Murray E. Development of a theory of implementation and integration: Normalization Process Theory. Implementation Science. 2009 Dec;4(1):1-9.

24. Nepesmo.com. 2017. NEPeSMO – Predict to Prevent. [online] Available at: <https://nepesmo.com/#:~:text=NEPeSMO%20offers%20a%20smart%20CE,to%20patients%20living%20with%20COPD> [Accessed 16 June 2021].

25. Pinnock H, Hanley J, McCloughan L, Todd A, Krishan A, Lewis S, et al. Effectiveness of telemonitoring integrated into existing clinical services on hospital admission for exacerbation of chronic obstructive pulmonary disease: researcher blind, multicentre, randomised controlled trial. Bmj. 2013;347:f6070

26. Sandeep K Gupta. Intention-to-treat concept: A Review. Perspect Clin Res 2011:2(3);109-112

27 . White IR, Royston P, Wood AM. Multiple imputation using chained equations. Stats in Medicine 2011, (30) 377-399.

28. Vrieze A, de Greef MH, Wijkstra PJ, Wempe JB. Low bone mineral density in COPD patients related to worse lung function, low weight and decreased fat-free mass. Osteoporos Int. 2007;18(9):1197-202.

29. Patel AR, Kowlessar BS, Donaldson GC, Mackay AJ, Singh R, George SN, et al. Cardiovascular risk, myocardial injury, and exacerbations of chronic obstructive pulmonary disease. Am J Respir Crit Care Med. 2013;188(9):1091-9.

30. Miller J, Edwards LD, Agusti A, Bakke P, Calverley PM, Celli B, et al. Comorbidity, systemic inflammation and outcomes in the ECLIPSE cohort. Respir Med. 2013;107(9):1376-84.

31. Global Initiative for Obstructive Lung Disease COPD Diagnosis and Management At-A-Glance Desk Reference 2017 [online] https://goldcopd.org/archived-reports/ Accessed 03/09/21

32. May C, Finch T. Implementing, Embedding, and Integrating Practices: An Outline of Normalization Process Theory. Sociology. 2009;43(3):535-54.

33. Ritchie J, Spencer L. Qualitative data analysis for applied policy research. In: Bryman A, Burgess RG, editors. London: Routledge; 1994.

34. Macfarlane A, O'Reilly-de Brún M. Using a theory-driven conceptual framework in qualitative health research. Qual Health Res. 2012;22(5):607-18.

For peer review only

35. O'Cathain A, Hoddinott P, Lewin S, Thomas KJ, Young B, Adamson J, et al. Maximising the impact of qualitative research in feasibility studies for randomised controlled trials: guidance for researchers. Pilot and Feasibility Studies. 2015;1(1):32.

Figure Legends

Figure 1: Flow diagram of the Predict & Prevent trial

Table Legends

Table 1: Schedule of assessments

For peer review only

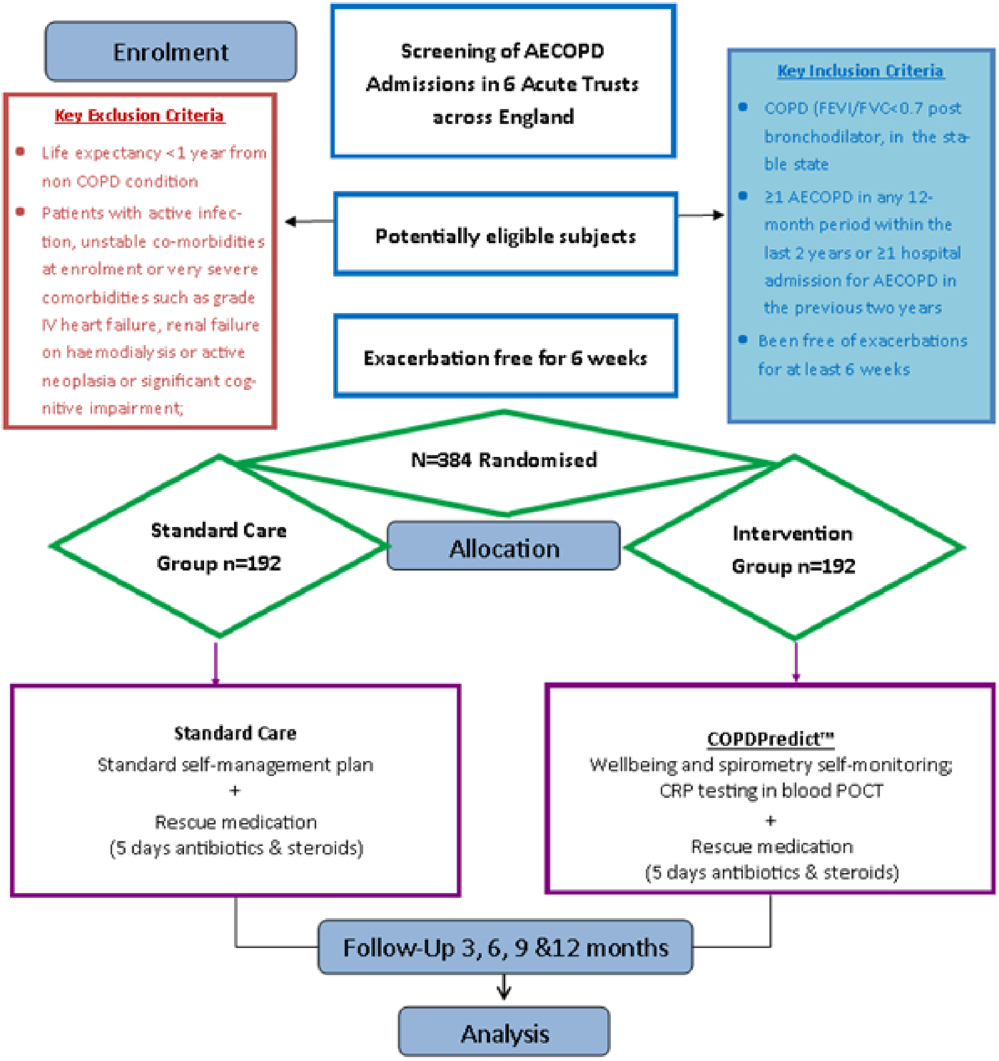

Figure 1: Flow diagram of the Predict & Prevent trial

266x297mm (57 x 57 DPI)

SPIRIT

STANDARD PROTOCOL ITEMS: RECOMMENDATIONS FOR INTERVENTIONAL TRIALS

SPIRIT 2013 Checklist: Recommended items to address in a clinical trial protocol and related documents*

Section/item	ItemNo	Description	Section of manuscript
**Administrative information**			
Title	1	Descriptive title identifying the study design, population, interventions, and, if applicable, trial acronym	Page 5 methods
Trial registration	2a	Trial identifier and registry name. If not yet registered, name of intended registry	Page 2 abstract
	2b	All items from the World Health Organization Trial Registration Data Set	
Protocol version	3	Date and version identifier	V9.0 vd03-Mar-22
Funding	4	Sources and types of financial, material, and other support	Page 2 NIHR Statement
Roles and responsibilities	5a	Names, affiliations, and roles of protocol contributors	Page 1
	5b	Name and contact information for the trial sponsor	Page 1 and 2
	5c	Role of study sponsor and funders, if any, in study design; collection, management, analysis, and interpretation of data; writing of the report; and the decision to submit the report for publication, including whether they will have ultimate authority over any of these activities	

For peer review only

	5d	Composition, roles, and responsibilities of the coordinating centre, steering committee, endpoint adjudication committee, data management team, and other individuals or groups overseeing the trial, if applicable (see Item 21a for data monitoring committee)	
**Introduction**			
Background and rationale	6a	Description of research question and justification for undertaking the trial, including summary of relevant studies (published and unpublished) examining benefits and harms for each intervention	Page 4-5
	6b	Explanation for choice of comparators	
Objectives	7	Specific objectives or hypotheses	Page 5
Trial design	8	Description of trial design including type of trial (eg, parallel group, crossover, factorial, single group), allocation ratio, and framework (eg, superiority, equivalence, noninferiority, exploratory)	Page 5-6
**Methods: Participants, interventions, and outcomes**			
Study setting	9	Description of study settings (eg, community clinic, academic hospital) and list of countries where data will be collected. Reference to where list of study sites can be obtained	Page 5

For peer review only

Eligibility criteria	10	Inclusion and exclusion criteria for participants. If applicable, eligibility criteria for study centres and individuals who will perform the interventions (eg, surgeons, psychotherapists)	Page 6
Interventions	11a	Interventions for each group with sufficient detail to allow replication, including how and when they will be administered	Page 7
	11b	Criteria for discontinuing or modifying allocated interventions for a given trial participant (eg, drug dose change in response to harms, participant request, or improving/worsening disease)	N/A
	11c	Strategies to improve adherence to intervention protocols, and any procedures for monitoring adherence (eg, drug tablet return, laboratory tests)	N/A
	11d	Relevant concomitant care and interventions that are permitted or prohibited during the trial	N/A
Outcomes	12	Primary, secondary, and other outcomes, including the specific measurement variable (eg, systolic blood pressure), analysis metric (eg, change from baseline, final value, time to event), method of aggregation (eg, median, proportion), and time point for each outcome. Explanation of the clinical relevance of chosen efficacy and harm outcomes is strongly recommended	Page 6/7

Participant timeline	13	Time schedule of enrolment, interventions (including any run-ins and washouts), assessments, and visits for participants. A schematic diagram is highly recommended (see Figure)	Page 10
Sample size	14	Estimated number of participants needed to achieve study objectives and how it was determined, including clinical and statistical assumptions supporting any sample size calculations	Page 5
Recruitment	15	Strategies for achieving adequate participant enrolment to reach target sample size	Page 8

Methods: Assignment of interventions (for controlled trials)

Allocation:

Sequence generation	16a	Method of generating the allocation sequence (eg, computer-generated random numbers), and list of any factors for stratification. To reduce predictability of a random sequence, details of any planned restriction (eg, blocking) should be provided in a separate document that is unavailable to those who enrol participants or assign interventions	Page 8 and 9
Allocation concealment mechanism	16b	Mechanism of implementing the allocation sequence (eg, central telephone; sequentially numbered, opaque, sealed envelopes), describing any steps to conceal the sequence until interventions are assigned	Protocol

Implementation	16c	Who will generate the allocation sequence, who will enrol participants, and who will assign participants to interventions	Protocol
Blinding (masking)	17a	Who will be blinded after assignment to interventions (eg, trial participants, care providers, outcome assessors, data analysts), and how	N/A
	17b	If blinded, circumstances under which unblinding is permissible, and procedure for revealing a participant's allocated intervention during the trial	
**Methods: Data collection, management, and analysis**			
Data collection methods	18a	Plans for assessment and collection of outcome, baseline, and other trial data, including any related processes to promote data quality (eg, duplicate measurements, training of assessors) and a description of study instruments (eg, questionnaires, laboratory tests) along with their reliability and validity, if known. Reference to where data collection forms can be found, if not in the protocol	Page 12-16 and Protocol
	18b	Plans to promote participant retention and complete follow-up, including list of any outcome data to be collected for participants who discontinue or deviate from intervention protocols	Page 12-13

For peer review only - http://bmjopen.bmj.com/site/about/guidelines.xhtml

Data management	19	Plans for data entry, coding, security, and storage, including any related processes to promote data quality (eg, double data entry; range checks for data values). Reference to where details of data management procedures can be found, if not in the protocol	Protocol
Statistical methods	20a	Statistical methods for analysing primary and secondary outcomes. Reference to where other details of the statistical analysis plan can be found, if not in the protocol	Protocol
	20b	Methods for any additional analyses (eg, subgroup and adjusted analyses)	Page 15-16
	20c	Definition of analysis population relating to protocol non-adherence (eg, as randomised analysis), and any statistical methods to handle missing data (eg, multiple imputation)	Page 14
**Methods: Monitoring**			
Data monitoring	21a	Composition of data monitoring committee (DMC); summary of its role and reporting structure; statement of whether it is independent from the sponsor and competing interests; and reference to where further details about its charter can be found, if not in the protocol. Alternatively, an explanation of why a DMC is not needed	Protocol

	21b	Description of any interim analyses and stopping guidelines, including who will have access to these interim results and make the final decision to terminate the trial	Protocol
Harms	22	Plans for collecting, assessing, reporting, and managing solicited and spontaneously reported adverse events and other unintended effects of trial interventions or trial conduct	Protocol
Auditing	23	Frequency and procedures for auditing trial conduct, if any, and whether the process will be independent from investigators and the sponsor	
**Ethics and dissemination**			
Research ethics approval	24	Plans for seeking research ethics committee/institutional review board (REC/IRB) approval	Page 16
Protocol amendments	25	Plans for communicating important protocol modifications (eg, changes to eligibility criteria, outcomes, analyses) to relevant parties (eg, investigators, REC/IRBs, trial participants, trial registries, journals, regulators)	Protocol
Consent or assent	26a	Who will obtain informed consent or assent from potential trial participants or authorised surrogates, and how (see Item 32)	Protocol
	26b	Additional consent provisions for collection and use of participant data and biological specimens in ancillary studies, if applicable	

For peer review only

Confidentiality	27	How personal information about potential and enrolled participants will be collected, shared, and maintained in order to protect confidentiality before, during, and after the trial	Protocol
Declaration of interests	28	Financial and other competing interests for principal investigators for the overall trial and each study site	Page 17
Access to data	29	Statement of who will have access to the final trial dataset, and disclosure of contractual agreements that limit such access for investigators	Protocol
Ancillary and post-trial care	30	Provisions, if any, for ancillary and post-trial care, and for compensation to those who suffer harm from trial participation	N/A
Dissemination policy	31a	Plans for investigators and sponsor to communicate trial results to participants, healthcare professionals, the public, and other relevant groups (eg, via publication, reporting in results databases, or other data sharing arrangements), including any publication restrictions	Page 16
	31b	Authorship eligibility guidelines and any intended use of professional writers	
	31c	Plans, if any, for granting public access to the full protocol, participant-level dataset, and statistical code	N/A

Appendices

review only

Informed consent materials	32	Model consent form and other related documentation given to participants and authorised surrogates	ICF
Biological specimens	33	Plans for collection, laboratory evaluation, and storage of biological specimens for genetic or molecular analysis in the current trial and for future use in ancillary studies, if applicable	N/A

*It is strongly recommended that this checklist be read in conjunction with the SPIRIT 2013 Explanation & Elaboration for important clarification on the items. Amendments to the protocol should be tracked and dated. The SPIRIT checklist is copyrighted by the SPIRIT Group under the Creative Commons "Attribution-NonCommercial-NoDerivs 3.0 Unported" license.

For peer review only

BMJ Open

A phase III, 2 arm, multi-centre, open label, parallel-group randomised designed clinical investigation of the use of a personalised early warning decision support system to predict and prevent acute exacerbations of Chronic Obstructive Pulmonary Disease - 'Predict & Prevent AECOPD' -Study Protocol

Journal:	*BMJ Open*
Manuscript ID	bmjopen-2022-061050.R2
Article Type:	Protocol
Date Submitted by the Author:	12-Oct-2022
Complete List of Authors:	Kaur, Dalbir; University of Birmingham,<br>Mehta, Rajnikant ; University of birmingham<br>Jarrett, Hugh; University of Birmingham<br>Jowett, Sue; University of Birmingham, Health Economics Unit<br>Gale, Nicola K.; University of Birmingham<br>Turner, Alice; University of Birmingham, Institute of Inflammation & Aging; Heart of England NHS Foundation Trust<br>Spiteri, Monica; University Hospitals of North Midlands NHS Trust<br>Patel, Neil; NEPESMO
**Primary Subject Heading**:	Respiratory medicine
Secondary Subject Heading:	Smoking and tobacco
Keywords:	RESPIRATORY MEDICINE (see Thoracic Medicine), Chronic airways disease < THORACIC MEDICINE, Thoracic medicine < INTERNAL MEDICINE

SCHOLARONE™ Manuscripts

A phase III, 2 arm, multi-centre, open label, parallel-group randomised designed clinical investigation of the use of a personalised early warning decision support system to predict and prevent acute exacerbations of Chronic Obstructive Pulmonary Disease - 'Predict & Prevent AECOPD' -Study Protocol

D Kaur, R Mehta, H Jarrett, S Jowett, N Gale, AM Turner, MA Spiteri, N Patel

Dalbir Kaur[1] Corresponding author
Email: dalbir.kaur@warwick.ac.uk

Rajnikant Mehta[2]
Email: R.L.Mehta@bham.ac.uk

Hugh Jarrett[3]
Email: h.jarrett@bham.ac.uk

Sue Jowett[4]
Email: S.JOWETT@bham.ac.uk

Nicola Gale[5]
Email: N.Gale@bham.ac.uk

Alice M Turner[6]
Email: A.M.Turner@bham.ac.uk

Monica A Spiteri[7]
Email: Monica.Spiteri@uhnm.nhs.uk

Neil Patel[8]
Email: neil.patel@nepesmo.com

[1]Senior Project Manager, Warwick Clinical Trials Unit (BWCTU), Warwick Medical School University of Warwick Coventry, CV4 7ALInstitute

[2]Senior Medical Statistician, Birmingham Clinical Trials Unit (BCTU), Institute of Applied Health Research, College of Medical and Dental Sciences, University of Birmingham, Edgbaston, Birmingham, B15 2TT

[3]Trials Management Team Leader, Birmingham Clinical Trials Unit (BCTU), Institute of Applied Health Research, College of Medical and Dental Sciences, University of Birmingham, Edgbaston, Birmingham, B15 2TT

[4]Professor of Health Economics, Health Economics Unit, Institute of Applied Health Research, College of Medical and Dental Sciences, University of Birmingham, Edgbaston, Birmingham, B15 2TT

[5]Professor of Health Policy and Sociology, Health Services Management Centre, School of Social Policy Director of Postgraduate Research, College of Social Sciences, University of Birmingham, Edgbaston, Birmingham, B15 2TT

[6] Professor of Respiratory Medicine and Honorary Consultant Respiratory Physician, Institute for Applied Health Research, University of Birmingham, Birmingham, UK, B15 2TT

For peer review only

[7]Director of Respiratory Research, Academic Research Unit, Courtyard Annexe - C Block, Royal Stoke University Hospital, University Hospitals of North Midlands NHS Trust, Newcastle Road, Staffordshire, UK, ST4 6QG

[8]Chief Medical Officer, NEPESMO Ltd, 76 King Street, Manchester, M2 4NH, United Kingdom
Word count: 3487 (excluding title page, abstract, table, legend and references)

Abstract

Introduction

With 65 million cases globally, chronic obstructive pulmonary disease (COPD) is the 4th leading cause of death and imposes a heavy burden on patients' lives and healthcare resources worldwide. Around half of all patients with COPD have frequent (≥2 per year) AECOPD[5]. Rapid readmissions are also common. Exacerbations impact significantly on COPD outcomes, causing significant lung function decline. Prompt exacerbation management optimises recovery and delays the time to the next acute episode.

Methods/analysis

The Predict & Prevent AECOPD trial is a phase III, 2 arm, multi-centre, open label, parallel-group individually randomised clinical trial investigating the use of a personalised early warning decision support system (COPDPredict™) to predict and prevent acute exacerbations of COPD. We aim to recruit 384 participants and randomise each individual in a 1:1 ratio to either standard self-management plans (SSMP) with Rescue Medication (RM) (control arm) or COPDPredict™ with RM (intervention arm)

The trial will inform the future standard of care regarding management of exacerbations in COPD patients. The main outcome measure is to provide further validation, as compared to usual care, for the clinical effectiveness of COPDPredict™ to help guide and support COPD patients and their respective clinical teams in identifying exacerbations early, with an aim to reduce the total number of AECOPD-induced hospital admissions in the 12 months following each patient's randomisation.

Ethics and Dissemination

This study protocol is reported in accordance with the guidance set out in the SPIRIT statement. Predict and Prevent AECOPD has obtained ethical approval in England (19/LO/1939). Upon completion of the trial and publication of results a lay findings summary will be disseminated to trial participants.

Trials Registration: Clinical trials.gov NCT04136418

Keywords: Respiratory, COPD, Exacerbations, Symptom Control Markers, COPDPredict™

NIHR Statement

This work is supported by the National Institute for Health Research (NIHR) under its Invention for Innovation (i4i) Programme (Grant Reference Number NIHR200002). The views expressed are those of the author(s) and not necessarily those of the NHS, the NIHR or the Department of Health.

For peer review only

Strengths and Limitations

- This study will be conducted as a phase 3 randomised controlled trial concerning an intervention for the early warning of COPD exacerbations, COPDPredict™, in patients with COPD. It will provide clinicians with a possible alternative for detecting COPD exacerbations early and actioning timely treatment changes to prevent unnecessary hospitalisation.
- An embedded economic and acceptability analysis will give practical information for rollout into clinical practice
- The study is limited to detecting effects on hospitalisations, and it is possible important changes in other aspects of COPD could be missed, as not powered to detect them.
- The requirement for blood CRP as an input to the prediction algorithm, and regular spirometry by patients introduces logistical challenges for staff and patients respectively

For peer review only

Background

With 65 million cases globally, chronic obstructive pulmonary disease (COPD) is the 4th leading cause of death and imposes a heavy burden on patients' lives and healthcare resources worldwide[1]. UK death rates are currently double the European Union (EU) average with over 30,000 people dying yearly and annual COPD-related NHS direct costs exceeding £800m[2]. Patients can have acute exacerbations of their COPD (AECOPD), which reduce quality of life, and lead to 140,000 emergency hospital admissions a year in the UK[3]. Indeed, AECOPD remain the 2nd commonest cause of emergency hospital admissions and 1 in 3 of these patients (sometimes more) will be readmitted within 3 months[4,5]. Hospitalisation itself carries a poor prognosis with an increased mortality risk.

Around half of all patients with COPD have frequent (≥2 per year) AECOPD[6,7]. Rapid readmissions are also common – the national COPD audit has shown that 43% of patients with COPD who were admitted are back in hospital within 90 days[8], and up to 71% by 12 months[9]. Exacerbations impact significantly on COPD outcomes, causing significant lung function decline[10]. Prompt exacerbation management optimises recovery and delays the time to the next acute episode[11], with NICE COPD guidelines highlighting a time window (prodrome) between an initial exacerbation's symptoms/signs and subsequent hospitalisation. Within this prodrome there is an opportunity to intervene. Current practice for COPD patients is that they are encouraged to recognise AECOPD, via standard self-management plans (SSMPs), and treat, using rescue medication (RM), but in many cases because of day-to-day variability in symptoms, the start of an exacerbation goes unrecognised and untreated. This inability/uncertainty to recognise and treat exacerbations in their early phase can lead to hospital admissions, which may drive long term decline.

Pharmacological and non-pharmacological treatments are used to reduce COPD exacerbations[12,13], and improve care, such as pulmonary rehabilitation (PR[14]), azithromycin[15], inhaled bronchodilators and inhaled corticosteroids. Infection control measures during the pandemic reduced spread of

For peer review only

other respiratory viruses, which in turn was associated with a 43% fall in admissions, with benefits maintained post-lockdown[16] though this is not currently part of international COPD guidelines[17, 18]. SSMPs help patients but have not really shown significant impact on A&E visits or hospital admissions as demonstrated by a systematic review of self-management strategies for COPD[19]. Intuitively early recognition and treatment of AECOPD would reduce exacerbation severity and duration, and improve prognosis; evidence for this is limited but supportive[20,21].

This study looks to address the problem by personalising and thus optimising effectiveness of AECOPD management using the CE marked and MHRA registered solution, COPDPredict™[22].

Methods

Patient and public involvement

Throughout the design and delivery of the trial public and participant involvement (PPI) has been accessed. In particular independent members on the trial steering committee provide oversight and contributions to the trial, including design and standardisation of trial-specific SSMP. PPI involvement has also extended to the qualitative research where the topic guide was developed in conjunction with respiratory participant groups.

Trial Design

The Predict & Prevent AECOPD trial is a phase III, 2 arm, multi-centre, open label, parallel-group individually randomised clinical trial investigating the use of a personalised early warning decision support system (COPDPredict™) to predict and prevent acute exacerbations of COPD. We aim to recruit 384 participants from NHS hospitals in the UK and randomise each individual in a 1:1 ratio to either SSMP with RM (control arm) or COPDPredict™ with RM (intervention arm) (*Figure 1*). The start date for the trial was 30th January 2019 and the end date for the trial is 30th March 2023.

The main outcome measure is to provide further validation, as compared to usual care, for the clinical effectiveness of COPDPredict™ to help guide and support COPD patients and their respective clinical teams in identifying exacerbations early, with an aim to reduce the total number of AECOPD-

For peer review only

induced hospital admissions in the 12 months following each patient's randomisation. The trial includes a health economic analysis examining health-related quality of life (HRQoL) using the validated questionnaire EQ-5D-5L and healthcare utilisation (as determined by a questionnaire that inquires as to hospitalisations, GP attendances and medication usage). A qualitative sub-study will assess both the end user experience and the healthcare provider experience, using Normalization Process Theory to understand the implementation and integration of the new technology[23].

Eligibility Criteria

Inclusion Criteria

- Clinically diagnosed COPD, confirmed by post-bronchodilator spirometry and defined as Post bronchodilator Forced expiratory volume/Forced vital capacity (FEV_1/FVC) <0.7 and <lower limit of normal, in the stable state
- ≥1 AECOPD in any 12-month period within the last 2 years or ≥1 hospital admission for AECOPD in the previous two years.
- Exacerbation free for at least 6 weeks
- An age of at least 18 years
- Willing and able to comply with data collection to 12 months from randomisation
- Ability to consent
- Ability to use intervention as judged by the investigator at screening, upon demonstration of the system to the patient

Exclusion Criteria

- Life expectancy <12 months -Assessment the individual investigator
- Co-enrolment into any clinical trials of investigative medicinal product (CTIMPs)
- Patients with selected co-morbidities, where these could impair use of the intervention (eg dementia)

For peer review only

Outcomes

Primary Outcome

Number of hospital admissions at 12 months post randomisation where the primary reason for admission is AECOPD. This will be obtained from participant testimony and from centrally held (HES) records. Data from the 2 sources will be cross-referenced to remove double-counting. The total number of admissions will be the sum of unique admissions from the 2 sources of data

Secondary Outcomes

Clinical Outcomes

Over a 12-month period, following randomisation;

Total in participant days, Number of visits to A&E, Number of participant defined exacerbations, Appropriate action on symptoms of AECOPD, as shown by the app, or participant testimony, Health-related quality of life (CAT) and, FEV_1 at 12 months post randomisation (from spirometry obtained during study visit)

Economic Outcomes

Health-related quality of life at baseline and 3, 6, 9 and 12-months post randomisation (EQ-5D-5L)

Healthcare utilisation at 3, 6, 9 and 12-months post randomisation, as determined by a questionnaire that inquires on hospitalisations, GP attendances and medication usage

Qualitative sub-study

End user experience and provider experience

Control Arm

A standard self-management plan (SSMP) alongside use of rescue medication (RM) containing 5 days of antibiotic and steroid treatment.

For peer review only

Intervention

COPDPredict™, consists of a patient-facing App (iOS/Android) and clinician-facing dashboard[24]. The Early Warning Decision Support System uses longitudinal remote monitoring of relevant subjective and objective data (symptoms, spirometry, biomarkers) in real-time via the App which is connected to CE-marked Bluetooth-enabled sensor peripherals. These data are used to construct COPD-relevant individual profiles such that artificial intelligence artificial intelligence (AI)-driven algorithms can then identify changes in health status to provide timely individualised alerts to patients and clinicians and sign-posting to action plans for patients.

COPDPredict™ also provides information around COPD self-management, pulmonary rehabilitation, inhaler technique and utilises gamification to help with adherence. Patients have full access to their results and can also directly message their clinical team. COPDPredict™ may signpost a participant to a self-management action plan for exacerbation management but clinical team supervision and oversight will remain throughout, as per usual care.

The clinician facing dashboard allows for "real-time" case management with the ability to remotely monitor patients and facilitate interaction. Clinicians can choose to escalate treatments based on the results being transmitted by the patients. A crucial dimension to this advanced system is that blood biomarker measurements which inform an algorithm are incorporated to enhance accuracy. Blood-based point of care (POC) analysers are used with COPDPredict™ and these POC analysers have previously been validated against laboratory CRP measures.

Recruitment and Consent

All patients must provide fully informed consent. A Participant Information Sheet (PIS) which explains the different ways in which the participant may enter the trial is provided to participants to facilitate this process. Investigators or delegate(s) adequately explain the aim, trial intervention, anticipated benefits and potential hazards of taking part in the trial to the participant. They also stress that participation is voluntary and that the participant is free to refuse to take part and may

For peer review only

withdraw from the trial at any time. Any trial specific procedures are completed after the participant has given informed consent. At each visit the participant's willingness to continue in the trial is ascertained and documented in the medical notes. Where new information becomes available which may affect the participants' decision to continue, participants will be given time to consider and if happy to continue will be re-consented. Re-consent will be documented in the medical notes.

Randomisation

After informed consent has been obtained and full participant eligibility confirmed (including exacerbation free for 6 weeks), the participant can be randomised into the trial. Randomisation CRFs are provided to investigators and may be used to collate the necessary information prior to randomisation. Participants are randomised by computer/telephone at the level of the individual in a 1:1 ratio to either SSMP with RM (control arm) or COPDPredict^{TM} with RM (intervention arm).

A minimisation algorithm will be used within the online randomisation system to ensure balance in the treatment allocation over centre, age (<60, ≥60 years) and severity of disease as per Global Initiative for Obstructive Lung Disease (GOLD)[1].

The participants are randomised to one of the following:

Control Arm

A standard self-management plan (SSMP) alongside use of rescue medication (RM) containing 5 days of antibiotic and steroid treatment.

Intervention arm

Supported self-management using the COPDPredict^{TM} App, involving personalised alerts to both patients and the clinical care team.

A 'random element' will be included in the minimisation algorithm, so that each participant has a probability, of being randomised to the opposite treatment that they would have otherwise

For peer review only

received. Once randomised patients will also be asked to complete baseline quality of life booklet which contains both the EQ-5D-5L and CAT questionnaire.

For peer review only

Assessment schedule

Visit	Hospital Discharge/ Initial appointment	Personal Baseline Day 1 after 6 weeks exacerbation free	Personal Baseline Day 7 (±2 days)	End of Personal Baseline Period	Approximately 4 times during any exacerbation (home visit or clinic)	Weekly until Week 52	Week 13 Telephone follow Up +/-1 week	Week 26 Hospital/ Telephone follow Up +/-1 week	Week 39 Telephone follow Up +/-1 week	Week 52 Telephone /Hospital follow Up +/- 2 weeks	Patients daily wellbeing completion post two-week bassline period
Eligibility check	X										
Valid informed consent	X										
Randomisation		X									
Height and Weight	X									X	
Concomitant medication	X	X		X				X	X	X	
Baseline medical history taken$^{\alpha}$	X										
Request to GP for rescue medication	X						(X)	(X)	(X)		
Assessment of (S)AEs	X				(X)		X	X	X	X	

For peer review only - http://bmjopen.bmj.com/site/about/guidelines.xhtml

Questionnaires (QoL and health utilisation)	X						X	X	X	X	
Spirometry	X							X		X	
Intervention only below this line					Intervention only below this line						
Provision of equipment & participant training		X									
Spirometry	X	X	X		(X)	X					
Symptom control markers	X	X	X	X	(X)	X	X	X	X	X	
Point of care blood test		X		X	(X)						
Clinical histories		X		X			X	X	X	X	
Wellbeing (App)											X

(X) denotes only when required. If rescue medication has not been used, fresh prescription requests will not be necessary

During the baseline period and any subsequent exacerbations patients will be entering well-being data on the App which will be related to lifestyle choices and the end-user experience. This data will not form part of the formal analysis of quality of life.

Table 1: Schedule of assessments

For peer review only

Statistical Analysis

Sample size

The justification of the sample size is based on previous evidence[25] that had shown a mean estimate of 2.5 COPD admissions in the previous year in the control group. To detect a difference of 1 admission in the mean number of admissions between groups using the standard methods of difference between means and assuming standard deviation of 2.6 with 90% power and a type I error rate of 5% (two-sided), 144 participants per group will need to be randomised, 288 in total. Assuming and adjusting for a 25% loss to follow-up/ drop-out rate, 384 participants will need to be recruited.

Analysis of Outcome Measures

The primary comparison groups will be composed of those randomised to the use of COPDPredict™ system and those randomised to the standard self-management plan (SSMP). All analyses will be based on the intention to treat principle[26], with all patients analysed in the treatment groups to which they were allocated irrespective of compliance with the randomised allocated treatment, and all patients will be included in the analyses. For all outcomes, summary statistics (e.g. mean differences, relative risks) will be reported and 95% confidence intervals will be constructed where appropriate. A two-sided p-value of <0.05 will be considered statistically significant.

Primary Outcome Measure

The primary analysis for this study will be to compare rates of hospital admission between the treatment groups (COPDPredict™ versus Usual care) over 12 months, following randomisation, where the primary reason for admission is an AECOPD event. These hospital admissions per person will be analysed using Poisson regression models adjusting for treatment group and minimisation variables. If there is over dispersion a negative binomial regression model adjusting for treatment group and the same minimisation variables will be taken into consideration. Point estimates

For peer review only - http://bmjopen.bmj.com/site/about/guidelines.xhtml

(incidence rates) will be provided and accompanied with 95% confidence intervals and respective p-values.

Secondary Outcome Measures

Recorded observations taken over a 12-month period, following randomisation:

Total in-hospital days and FEV_1 – These variables will be summarised using basic descriptive statistics (Mean, SD)). We may also consider a mixed linear regression model, to estimate differences between the intervention group supported with 95% CI adjusting for baseline variables and minimisation variables (centre being a random effects variable).

Health-related quality of life questionnaires - EQ5D-5L and CAT will be converted into scores and analysed using a mixed linear regression model, adjusting for the intervention group, baseline and minimisation variables as before.

Number of A&E visits, participant defined exacerbations and healthcare utilisation - Where the participant experienced an episode, the data during the 12 months from randomisation will be analysed using mixed effects log-binomial regression techniques with presentation of relative risk and 95% confidence intervals. Furthermore, sensitivity analysis based on patients' experience of multiple visits to A&E and participant defined exacerbation will be calculated using Poisson regression techniques and relative risk supported with 95% CI.

Symptom control marker association to clinical decision - The diagnostic accuracy of COPDPredict™ and Usual care will be evaluated by calculating sensitivity, specificity, positive and negative predictive values, and area under curve (AUC)/receiver operating characteristics (ROC) together with 95% CI.

Subgroup analyses

Subgroup analyses will be limited to the same variables used in the minimisation algorithm, apart from centre. Tests for statistical heterogeneity (e.g. by including the treatment group by subgroup

For peer review only - http://bmjopen.bmj.com/site/about/guidelines.xhtml

interaction parameter in the statistical model) will be performed prior to any examination of effect estimate within subgroups. The results of subgroup analyses will be treated with caution and will be used for the purposes of hypothesis generation only.

Missing data and sensitivity analyses

Participants with missing primary outcome data will not be included in the primary analysis in the first instance. This presents a risk of bias, and sensitivity analyses will be undertaken to assess the possible impact. This will consist of simulating the missing response using a multiple imputation approach[27]. Parameters used to simulate the missing response will include the minimisation variables, intervention group and previous response. Full details are included in the statistical analysis plan (SAP).

Adverse events and serious adverse events

Patients with COPD can have high disease burden, such that the trial population will be older, with co-morbidities such as osteoporosis[28], cardiovascular disease or raised cardiovascular risk[29], along with associated complications and symptoms including abnormal lab results. A relatively high number of adverse events are anticipated as a result of the patients' existing past medical history[30]. However, this study is examining the role of increased participant surveillance and therefore few, if any, foreseeable risks of direct harm associated with the study intervention are anticipated. AE reporting will therefore be limited to those events which are required for trial monitoring or outcome assessment.

All events which meet the definition of serious must be recorded in the participant notes, but for trial purposes these following events do not require reporting on the SAE Form. Such events are "safety reporting exempt".

• Planned hospitalisation for a pre-existing condition, or a procedure required by the trial protocol without serious deterioration in health, is not considered a serious adverse event.

For peer review only - http://bmjopen.bmj.com/site/about/guidelines.xhtml

• An overnight stay in hospital that is due to transportation, organisational or accommodation problems, and without medical background SAES requiring expedited reporting

Any other events that fulfil the usual definition of an SAE will be reported immediately.

Any death occurring during the Trial Protocol defined follow up period (12 months) must be reported as an SAE within 24 hours of the local investigator becoming aware of the event.

Health economics

Within -trial analysis

The economic evaluation alongside the trial will take the form of an incremental cost-effectiveness analysis to estimate cost per hospital admission avoided and incremental cost-utility analysis to estimate cost per quality adjusted life year (QALY) gained. Base-case analyses will be from a healthcare perspective over 12 months follow-up using individual participant level data on costs and outcomes from the clinical trial. Additional analyses will be undertaken from a broader societal perspective. Resource use data will be collected by questionnaire, capturing information on hospitalisations for exacerbations, COPD-related primary and secondary care attendances and medication usage. Data will also be collected during the trial on resource use implications of the intervention. Information on participant incurred costs, time off work and impact on activities is collected within the economic questionnaire. QALYs will be calculated from EQ-5D-5L responses. Sensitivity analysis will be undertaken and cost-effectiveness planes and cost-effectiveness acceptability curves presented.

Model-based analysis

A decision model will be constructed to extrapolate beyond trial results over five years, with costs and benefits discounted at 3.5%. The model will be the first decision analysis to use GOLD stages ABCD[1] and will consider the ability of the COPDPredict™ supported self-management to impact movements between GOLD ABCD stages, but also the number of exacerbations within those stages.

For peer review only - http://bmjopen.bmj.com/site/about/guidelines.xhtml

Costs associated with delivering COPDPredict™, exacerbations, and routine care will be obtained from the trial. Quality of life scores on each intervention will also be obtained from trial data using EQ-5D-5L. Longer-term costs, quality of life scores, and transitions between stages, and number of exacerbations will be obtained from analysis of ABCD cohorts from within the BLISS[2] study, a prospective cohort study with 3-year follow-up period. The model will estimate cost per QALY gained of COPDPredict™ supported self-management versus standard self-management from a healthcare perspective. Probabilistic sensitivity analysis will be undertaken to simultaneously incorporate all parameter uncertainty. Cost-effectiveness planes and cost-effectiveness acceptability curves will be presented to show the probability the intervention is cost-effective at different cost/QALY thresholds. A value of information analysis will assess the value of further trials to reduce parameter uncertainty and identify parameters which would be most valuable in reducing that uncertainty[31].

Qualitative sub-study

In order to frame this study, we draw on Normalisation Process Theory[32], which proposes four constructs essential to effect implementation of new technologies: *coherence* (sense making work to understand the possibilities of a new technology), *cognitive participation* (relational work as new technology begins to be used), *collective action* (operational work enacted to make a technology function in context) and *reflexive monitoring* (the appraisal of work that people do to assess how the new technology affects them and others). Participants will include health care professionals, patients and wider policy stakeholders and will be purposively sampled to maximise diversity. A topic guide developed by drawing on existing literature and theory will focus on attitudes to and practices around self-management of COPD and the use and implementation of digital technologies. Interviews will be audio recorded and transcribed verbatim, before subjecting the data to a two-stage approach to analysis[336]: first, a descriptive framework analysis[34] followed by mapping onto the NPT constructs. We anticipate conducting approximately30 interviews but this will be informed by the data and analysis35].

For peer review only - http://bmjopen.bmj.com/site/about/guidelines.xhtml

Ethics and dissemination:

This study protocol is reported in accordance with the guidance set out in the SPIRIT statement. Predict and Prevent AECOPD has obtained ethical approval in England (19/LO/1939). Upon completion of the trial and publication of results a lay findings summary will be disseminated to trial participants.

Trial Oversight

Trial oversight will be provided by the TMG, an independent DMC and independent TSC. Interim analyses of major outcome measures and safety data will be conducted and provided in strict confidence to the DMC.

Potential Impact

Predict & Prevent AECOPD is a pragmatic, national, multicentre, RCT which aims to provide robust evidence around the clinical and cost effectiveness of COPDPredict™ by analysing the number of patients admitted to hospital in a 12month period post randomisation. It is hoped the outcome of this trial could be used to provide robust evidence to the management of patients with COPD and their exacerbations in the future.

Contributorship statement

All authors have made substantial contributions to the conception or design of the work, or the acquisition, analysis or interpretation of data. DK, AT, HJ, RM, SJ, NG, NP and MS drafted the manuscript and revised it critically for important intellectual content. All authors approved final version and agreed to be accountable for all aspects of the work in ensuring that questions related to the accuracy or integrity of any part of the work are appropriately investigated and resolved.

Competing Interest Statement

The CI of the trial (AT) does not have any relevant direct financial disclosures, nor do members of the trial management group (TMG) who are authors of this paper, with the exception of NP who is a

For peer review only

founder, director and share-holder of NEPeSMO, who developed the intervention. In addition, MS is CI of the overall NIHR-funded project and is a founder, director and share-holder of NEPeSMO. NEPeSMO is a start-up company from the University Hospitals of North Midlands NHS Trust, owns all intellectual property rights of COPDPredict™ and is a project collaborator on the grant.

AT has grants from pharmaceutical companies working in the area of COPD (Chiesi, AstraZeneca) and has conducted advisory work for such (Boehringer, CSL Behring) but not in the area of medical devices or admission prevention. Neither has she worked for, or received monies from, any company working on admission prevention in the last 3 years.

Funding Statement & Disclaimer

This report is independent research funded by the National Institute for Health Research (Invention for Innovation, A Personalised Early Warning Decision Support System with novel Saliva Bio-Profiling to Predict and Prevent Acute Exacerbations of Chronic Obstructive Pulmonary Disease – 'Predict&prevent AECOPD, NIHR200002).

For peer review only

References

1. Global Initiative for Obstructive Lung Disease, [Accessed 13 June 2018]. [Available from: www.goldcopd.com].

2. Estimating the economic burden of respiratory illness in the UK. London: British Lung Foundation 2016.

3. The battle for breath - the impact of lung disease in the UK. London: British Lung Foundation, 2016.

4. Roberts CM, Stone RA, Lowe D, Pursey NA, Buckingham RJ. Co-morbidities and 90-day outcomes in hospitalized COPD exacerbations. Copd. 2011;8(5):354-61.

5. Stone RA HB, Lowe D, Searle L, Skipper E, Welham S, et al. . National COPD audit programme. COPD: Who cares matters. London, UK: Royal College of Physicians, 2015. Contract No.: eISBN 978-1-86016-559-7.

6. Hurst JR, Vestbo J, Anzueto A, Locantore N, Mullerova H, Tal-Singer R, et al. Susceptibility to exacerbation in chronic obstructive pulmonary disease. N Engl J Med. 2010;363(12):1128-38.

7. Merinopoulou E, Raluy-Callado M, Ramagopalan S, MacLachlan S, Khalid JM. COPD exacerbations by disease severity in England. Int J Chron Obstruct Pulmon Dis. 2016;11:697-709.

8. Stone RA HB, McMillan V, Saleem Khan M, Searle L, Skipper E, et al. COPD: Who cares when it matters most? London: Royal College of Physicians, 2017.

9. Steer J, Gibson GJ, Bourke SC. Longitudinal change in quality of life following hospitalisation for acute exacerbations of COPD. BMJ Open Respir Res. 2015;2(1):e000069.

10. Jo YS, Kim SK, Park SJ, Um S-J, Park Y-B, Jung KS, et al. Longitudinal change of FEV(1) and inspiratory capacity: clinical implication and relevance to exacerbation risk in patients with COPD. Int J Chron Obstruct Pulmon Dis. 2019;14:361-9.

For peer review only

11. Qureshi H SA, Hanania NA. Chronic obstructive pulmonary disease exacerbations: latest evidence and clinical implications. Therapeutic advances in chronic disease. 2014;5(5):212-27. Epub 2014/09/02.

12. Oba, Y., Keeney, E., Ghatehorde, N. and Dias, S., 2018. Dual combination therapy versus long-acting bronchodilators alone for chronic obstructive pulmonary disease (COPD): a systematic review and network meta-analysis. Cochrane Database of Systematic Reviews, [online] 2018(12). Available at: <https://www.cochranelibrary.com/cdsr/doi/10.1002/14651858.CD012620.pub2/full> [Accessed 5 October 2021].

13. Yang, I., Clarke, M., Sim, E. and Fong, K., 2012. Inhaled corticosteroids for stable chronic obstructive pulmonary disease. Cochrane Database of Systematic Reviews, [online] Available at: <http://Inhaled corticosteroids for stable chronic obstructive pulmonary disease> [Accessed 1 September 2021].

14. Ryrso CK, Godtfredsen NS, Kofod LM, Lavesen M, Mogensen L, Tobberup R, et al. Lower mortality after early supervised pulmonary rehabilitation following COPD-exacerbations: a systematic review and meta-analysis. BMC Pulm Med. 2018;18(1):154.

15. Ni, W., Shao, X., Cai, X., Wei, C., Cui, J., Wang, R. and Liu, Y., 2015. Prophylactic Use of Macrolide Antibiotics for the Prevention of Chronic Obstructive Pulmonary Disease Exacerbation: A Meta-Analysis. PLOS ONE, 10(3), p.e0121257.

16. Lawless, M., Burgess, M. and Bourke, S., 2022. Impact of COVID-19 on Hospital Admissions for COPD Exacerbation: Lessons for Future Care. Medicina, 58(1), p.66.

17. Nice.org.uk. 2018. Overview | Chronic obstructive pulmonary disease in over 16s: diagnosis and management | Guidance | NICE. [online] Available at: <https://www.nice.org.uk/guidance/ng115> [Accessed 23 July 2022].

18. Global Initiative for Chronic Obstructive Lung Disease - GOLD. 2022. 2022 GOLD Reports - Global Initiative for Chronic Obstructive Lung Disease - GOLD. [online] Available at: <https://goldcopd.org/2022-gold-reports-2/> [Accessed 23 July 2022].

19. Jolly K, Majothi S, Sitch AJ, Heneghan NR, Riley RD, Moore DJ, et al. Self-management of health care behaviors for COPD: a systematic review and meta-analysis. Int J Chron Obstruct Pulmon Dis. 2016;11:305-26.

20. Vijayasaratha K, Stockley RA. Relationship between frequency, length, and treatment outcome of exacerbations to baseline lung function and lung density in alpha-1 antitrypsin-deficient COPD. Int J Chron Obstruct Pulmon Dis. 2012;7:789-96.

21. Celli BR, Locantore N, Yates J, Tal-Singer R, Miller BE, Bakke P, et al. Inflammatory biomarkers improve clinical prediction of mortality in chronic obstructive pulmonary disease. Am J Respir Crit Care Med. 2012;185(10):1065-72.

For peer review only

22. Patel N, Kinmond K, Jones P, Birks P, Spiteri MA. Validation of COPDPredict™: Unique Combination of Remote Monitoring and Exacerbation Prediction to Support Preventative Management of COPD Exacerbations. International Journal of Chronic Obstructive Pulmonary Disease 2021, 16:1887-1899

23. May CR, Mair F, Finch T, MacFarlane A, Dowrick C, Treweek S, Rapley T, Ballini L, Ong BN, Rogers A, Murray E. Development of a theory of implementation and integration: Normalization Process Theory. Implementation Science. 2009 Dec;4(1):1-9.

24. Nepesmo.com. 2017. NEPeSMO – Predict to Prevent. [online] Available at: <https://nepesmo.com/#:~:text=NEPeSMO%20offers%20a%20smart%20CE,to%20patients%20living%20with%20COPD> [Accessed 16 June 2021].

25. Pinnock H, Hanley J, McCloughan L, Todd A, Krishan A, Lewis S, et al. Effectiveness of telemonitoring integrated into existing clinical services on hospital admission for exacerbation of chronic obstructive pulmonary disease: researcher blind, multicentre, randomised controlled trial. Bmj. 2013;347:f6070

26. Sandeep K Gupta. Intention-to-treat concept: A Review. Perspect Clin Res 2011:2(3);109-112

27 . White IR, Royston P, Wood AM. Multiple imputation using chained equations. Stats in Medicine 2011, (30) 377-399.

28. Vrieze A, de Greef MH, Wijkstra PJ, Wempe JB. Low bone mineral density in COPD patients related to worse lung function, low weight and decreased fat-free mass. Osteoporos Int. 2007;18(9):1197-202.

29. Patel AR, Kowlessar BS, Donaldson GC, Mackay AJ, Singh R, George SN, et al. Cardiovascular risk, myocardial injury, and exacerbations of chronic obstructive pulmonary disease. Am J Respir Crit Care Med. 2013;188(9):1091-9.

30. Miller J, Edwards LD, Agusti A, Bakke P, Calverley PM, Celli B, et al. Comorbidity, systemic inflammation and outcomes in the ECLIPSE cohort. Respir Med. 2013;107(9):1376-84.

31. Global Initiative for Obstructive Lung Disease COPD Diagnosis and Management At-A-Glance Desk Reference 2017 [online] https://goldcopd.org/archived-reports/ Accessed 03/09/21

32. May C, Finch T. Implementing, Embedding, and Integrating Practices: An Outline of Normalization Process Theory. Sociology. 2009;43(3):535-54.

33. Ritchie J, Spencer L. Qualitative data analysis for applied policy research. In: Bryman A, Burgess RG, editors. London: Routledge; 1994.

34. Macfarlane A, O'Reilly-de Brún M. Using a theory-driven conceptual framework in qualitative health research. Qual Health Res. 2012;22(5):607-18.

For peer review only

35. O'Cathain A, Hoddinott P, Lewin S, Thomas KJ, Young B, Adamson J, et al. Maximising the impact of qualitative research in feasibility studies for randomised controlled trials: guidance for researchers. Pilot and Feasibility Studies. 2015;1(1):32.

Figure Legends

Figure 1: Flow diagram of the Predict & Prevent trial

Table Legends

Table 1: Schedule of assessments

For peer review only

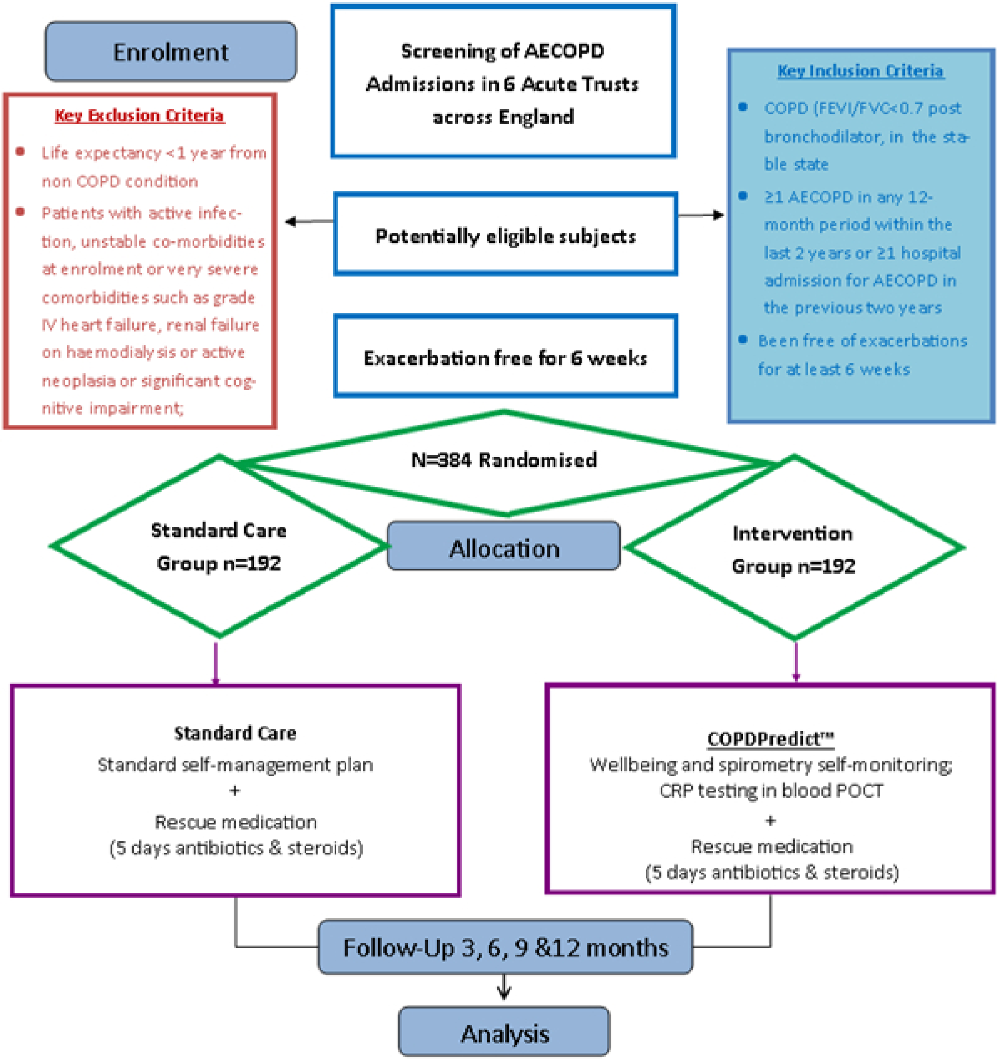

Figure 1: Flow diagram of the Predict & Prevent trial

266x297mm (57 x 57 DPI)

SPIRIT
STANDARD PROTOCOL ITEMS: RECOMMENDATIONS FOR INTERVENTIONAL TRIALS

SPIRIT 2013 Checklist: Recommended items to address in a clinical trial protocol and related documents*

Section/item	ItemNo	Description	Section of manuscript
**Administrative information**			
Title	1	Descriptive title identifying the study design, population, interventions, and, if applicable, trial acronym	Page 5 methods
Trial registration	2a	Trial identifier and registry name. If not yet registered, name of intended registry	Page 2 abstract
	2b	All items from the World Health Organization Trial Registration Data Set	
Protocol version	3	Date and version identifier	V9.0 vd03-Mar-22
Funding	4	Sources and types of financial, material, and other support	Page 2 NIHR Statement
Roles and responsibilities	5a	Names, affiliations, and roles of protocol contributors	Page 1
	5b	Name and contact information for the trial sponsor	Page 1 and 2
	5c	Role of study sponsor and funders, if any, in study design; collection, management, analysis, and interpretation of data; writing of the report; and the decision to submit the report for publication, including whether they will have ultimate authority over any of these activities	

For peer review only

	5d	Composition, roles, and responsibilities of the coordinating centre, steering committee, endpoint adjudication committee, data management team, and other individuals or groups overseeing the trial, if applicable (see Item 21a for data monitoring committee)	
**Introduction**			
Background and rationale	6a	Description of research question and justification for undertaking the trial, including summary of relevant studies (published and unpublished) examining benefits and harms for each intervention	Page 4-5
	6b	Explanation for choice of comparators	
Objectives	7	Specific objectives or hypotheses	Page 5
Trial design	8	Description of trial design including type of trial (eg, parallel group, crossover, factorial, single group), allocation ratio, and framework (eg, superiority, equivalence, noninferiority, exploratory)	Page 5-6
**Methods: Participants, interventions, and outcomes**			
Study setting	9	Description of study settings (eg, community clinic, academic hospital) and list of countries where data will be collected. Reference to where list of study sites can be obtained	Page 5

For peer review only

Eligibility criteria	10	Inclusion and exclusion criteria for participants. If applicable, eligibility criteria for study centres and individuals who will perform the interventions (eg, surgeons, psychotherapists)	Page 6
Interventions	11a	Interventions for each group with sufficient detail to allow replication, including how and when they will be administered	Page 7
	11b	Criteria for discontinuing or modifying allocated interventions for a given trial participant (eg, drug dose change in response to harms, participant request, or improving/worsening disease)	N/A
	11c	Strategies to improve adherence to intervention protocols, and any procedures for monitoring adherence (eg, drug tablet return, laboratory tests)	N/A
	11d	Relevant concomitant care and interventions that are permitted or prohibited during the trial	N/A
Outcomes	12	Primary, secondary, and other outcomes, including the specific measurement variable (eg, systolic blood pressure), analysis metric (eg, change from baseline, final value, time to event), method of aggregation (eg, median, proportion), and time point for each outcome. Explanation of the clinical relevance of chosen efficacy and harm outcomes is strongly recommended	Page 6/7

Participant timeline	13	Time schedule of enrolment, interventions (including any run-ins and washouts), assessments, and visits for participants. A schematic diagram is highly recommended (see Figure)	Page 10
Sample size	14	Estimated number of participants needed to achieve study objectives and how it was determined, including clinical and statistical assumptions supporting any sample size calculations	Page 5
Recruitment	15	Strategies for achieving adequate participant enrolment to reach target sample size	Page 8

Methods: Assignment of interventions (for controlled trials)

Allocation:

Sequence generation	16a	Method of generating the allocation sequence (eg, computer-generated random numbers), and list of any factors for stratification. To reduce predictability of a random sequence, details of any planned restriction (eg, blocking) should be provided in a separate document that is unavailable to those who enrol participants or assign interventions	Page 8 and 9
Allocation concealment mechanism	16b	Mechanism of implementing the allocation sequence (eg, central telephone; sequentially numbered, opaque, sealed envelopes), describing any steps to conceal the sequence until interventions are assigned	Protocol

Implementation	16c	Who will generate the allocation sequence, who will enrol participants, and who will assign participants to interventions	Protocol
Blinding (masking)	17a	Who will be blinded after assignment to interventions (eg, trial participants, care providers, outcome assessors, data analysts), and how	N/A
	17b	If blinded, circumstances under which unblinding is permissible, and procedure for revealing a participant's allocated intervention during the trial	
**Methods: Data collection, management, and analysis**			
Data collection methods	18a	Plans for assessment and collection of outcome, baseline, and other trial data, including any related processes to promote data quality (eg, duplicate measurements, training of assessors) and a description of study instruments (eg, questionnaires, laboratory tests) along with their reliability and validity, if known. Reference to where data collection forms can be found, if not in the protocol	Page 12-16 and Protocol
	18b	Plans to promote participant retention and complete follow-up, including list of any outcome data to be collected for participants who discontinue or deviate from intervention protocols	Page 12-13

Data management	19	Plans for data entry, coding, security, and storage, including any related processes to promote data quality (eg, double data entry; range checks for data values). Reference to where details of data management procedures can be found, if not in the protocol	Protocol
Statistical methods	20a	Statistical methods for analysing primary and secondary outcomes. Reference to where other details of the statistical analysis plan can be found, if not in the protocol	Protocol
	20b	Methods for any additional analyses (eg, subgroup and adjusted analyses)	Page 15-16
	20c	Definition of analysis population relating to protocol non-adherence (eg, as randomised analysis), and any statistical methods to handle missing data (eg, multiple imputation)	Page 14
**Methods: Monitoring**			
Data monitoring	21a	Composition of data monitoring committee (DMC); summary of its role and reporting structure; statement of whether it is independent from the sponsor and competing interests; and reference to where further details about its charter can be found, if not in the protocol. Alternatively, an explanation of why a DMC is not needed	Protocol

	21b	Description of any interim analyses and stopping guidelines, including who will have access to these interim results and make the final decision to terminate the trial	Protocol
Harms	22	Plans for collecting, assessing, reporting, and managing solicited and spontaneously reported adverse events and other unintended effects of trial interventions or trial conduct	Protocol
Auditing	23	Frequency and procedures for auditing trial conduct, if any, and whether the process will be independent from investigators and the sponsor	
**Ethics and dissemination**			
Research ethics approval	24	Plans for seeking research ethics committee/institutional review board (REC/IRB) approval	Page 16
Protocol amendments	25	Plans for communicating important protocol modifications (eg, changes to eligibility criteria, outcomes, analyses) to relevant parties (eg, investigators, REC/IRBs, trial participants, trial registries, journals, regulators)	Protocol
Consent or assent	26a	Who will obtain informed consent or assent from potential trial participants or authorised surrogates, and how (see Item 32)	Protocol
	26b	Additional consent provisions for collection and use of participant data and biological specimens in ancillary studies, if applicable	

For peer review only

Confidentiality	27	How personal information about potential and enrolled participants will be collected, shared, and maintained in order to protect confidentiality before, during, and after the trial	Protocol
Declaration of interests	28	Financial and other competing interests for principal investigators for the overall trial and each study site	Page 17
Access to data	29	Statement of who will have access to the final trial dataset, and disclosure of contractual agreements that limit such access for investigators	Protocol
Ancillary and post-trial care	30	Provisions, if any, for ancillary and post-trial care, and for compensation to those who suffer harm from trial participation	N/A
Dissemination policy	31a	Plans for investigators and sponsor to communicate trial results to participants, healthcare professionals, the public, and other relevant groups (eg, via publication, reporting in results databases, or other data sharing arrangements), including any publication restrictions	Page 16
	31b	Authorship eligibility guidelines and any intended use of professional writers	
	31c	Plans, if any, for granting public access to the full protocol, participant-level dataset, and statistical code	N/A

Appendices

review only

Informed consent materials	32	Model consent form and other related documentation given to participants and authorised surrogates	ICF
Biological specimens	33	Plans for collection, laboratory evaluation, and storage of biological specimens for genetic or molecular analysis in the current trial and for future use in ancillary studies, if applicable	N/A

*It is strongly recommended that this checklist be read in conjunction with the SPIRIT 2013 Explanation & Elaboration for important clarification on the items. Amendments to the protocol should be tracked and dated. The SPIRIT checklist is copyrighted by the SPIRIT Group under the Creative Commons "Attribution-NonCommercial-NoDerivs 3.0 Unported" license.

For peer review only

BMJ Open

A phase III, 2 arm, multi-centre, open label, parallel-group randomised designed clinical investigation of the use of a personalised early warning decision support system to predict and prevent acute exacerbations of Chronic Obstructive Pulmonary Disease - 'Predict & Prevent AECOPD' -Study Protocol

Journal:	*BMJ Open*
Manuscript ID	bmjopen-2022-061050.R3
Article Type:	Protocol
Date Submitted by the Author:	27-Oct-2022

Complete List of Authors:	Kaur, Dalbir; University of Warwick<br>Mehta, Rajnikant ; University of birmingham<br>Jarrett, Hugh; University of Birmingham<br>Jowett, Sue; University of Birmingham, Health Economics Unit<br>Gale, Nicola K.; University of Birmingham<br>Turner, Alice; University of Birmingham, Institute of Inflammation & Aging; Heart of England NHS Foundation Trust<br>Spiteri, Monica; University Hospitals of North Midlands NHS Trust<br>Patel, Neil; NEPESMO

**Primary Subject Heading**:	Respiratory medicine
Secondary Subject Heading:	Smoking and tobacco
Keywords:	RESPIRATORY MEDICINE (see Thoracic Medicine), Chronic airways disease < THORACIC MEDICINE, Thoracic medicine < INTERNAL MEDICINE

SCHOLARONE™
Manuscripts

A phase III, 2 arm, multi-centre, open label, parallel-group randomised designed clinical investigation of the use of a personalised early warning decision support system to predict and prevent acute exacerbations of Chronic Obstructive Pulmonary Disease - 'Predict & Prevent AECOPD' -Study Protocol

D Kaur, R Mehta, H Jarrett, S Jowett, N Gale, AM Turner, MA Spiteri, N Patel

Dalbir Kaur[1] Corresponding author
Email: dalbir.kaur@warwick.ac.uk

Rajnikant Mehta[2]
Email: R.L.Mehta@bham.ac.uk

Hugh Jarrett[3]
Email: h.jarrett@bham.ac.uk

Sue Jowett[4]
Email: S.JOWETT@bham.ac.uk

Nicola Gale[5]
Email: N.Gale@bham.ac.uk

Alice M Turner[6]
Email: A.M.Turner@bham.ac.uk

Monica A Spiteri[7]
Email: Monica.Spiteri@uhnm.nhs.uk

Neil Patel[8]
Email: neil.patel@nepesmo.com

[1]Senior Project Manager, Warwick Clinical Trials Unit (BWCTU), Warwick Medical School University of Warwick Coventry, CV4 7ALInstitute

[2]Senior Medical Statistician, Birmingham Clinical Trials Unit (BCTU), Institute of Applied Health Research, College of Medical and Dental Sciences, University of Birmingham, Edgbaston, Birmingham, B15 2TT

[3]Trials Management Team Leader, Birmingham Clinical Trials Unit (BCTU), Institute of Applied Health Research, College of Medical and Dental Sciences, University of Birmingham, Edgbaston, Birmingham, B15 2TT

[4]Professor of Health Economics, Health Economics Unit, Institute of Applied Health Research, College of Medical and Dental Sciences, University of Birmingham, Edgbaston, Birmingham, B15 2TT

[5]Professor of Health Policy and Sociology, Health Services Management Centre, School of Social Policy Director of Postgraduate Research, College of Social Sciences, University of Birmingham, Edgbaston, Birmingham, B15 2TT

[6] Professor of Respiratory Medicine and Honorary Consultant Respiratory Physician, Institute for Applied Health Research, University of Birmingham, Birmingham, UK, B15 2TT

For peer review only

[7]Director of Respiratory Research, Academic Research Unit, Courtyard Annexe - C Block, Royal Stoke University Hospital, University Hospitals of North Midlands NHS Trust, Newcastle Road, Staffordshire, UK, ST4 6QG

[8]Chief Medical Officer, NEPESMO Ltd, 76 King Street, Manchester, M2 4NH, United Kingdom
Word count: 3487 (excluding title page, abstract, table, legend and references)

Abstract

Introduction

With 65 million cases globally, chronic obstructive pulmonary disease (COPD) is the 4th leading cause of death and imposes a heavy burden on patients' lives and healthcare resources worldwide. Around half of all patients with COPD have frequent (≥2 per year) AECOPD[5]. Rapid readmissions are also common. Exacerbations impact significantly on COPD outcomes, causing significant lung function decline. Prompt exacerbation management optimises recovery and delays the time to the next acute episode.

Methods/analysis

The Predict & Prevent AECOPD trial is a phase III, 2 arm, multi-centre, open label, parallel-group individually randomised clinical trial investigating the use of a personalised early warning decision support system (COPDPredict™) to predict and prevent acute exacerbations of COPD. We aim to recruit 384 participants and randomise each individual in a 1:1 ratio to either standard self-management plans (SSMP) with Rescue Medication (RM) (control arm) or COPDPredict™ with RM (intervention arm)

The trial will inform the future standard of care regarding management of exacerbations in COPD patients. The main outcome measure is to provide further validation, as compared to usual care, for the clinical effectiveness of COPDPredict™ to help guide and support COPD patients and their respective clinical teams in identifying exacerbations early, with an aim to reduce the total number of AECOPD-induced hospital admissions in the 12 months following each patient's randomisation.

Ethics and Dissemination

This study protocol is reported in accordance with the guidance set out in the SPIRIT statement. Predict and Prevent AECOPD has obtained ethical approval in England (19/LO/1939). Upon completion of the trial and publication of results a lay findings summary will be disseminated to trial participants.

Trials Registration: Clinical trials.gov NCT04136418

Keywords: Respiratory, COPD, Exacerbations, Symptom Control Markers, COPDPredict™

NIHR Statement

This work is supported by the National Institute for Health Research (NIHR) under its Invention for Innovation (i4i) Programme (Grant Reference Number NIHR200002). The views expressed are those of the author(s) and not necessarily those of the NHS, the NIHR or the Department of Health.

For peer review only

Strengths and Limitations

- This study will be conducted as a phase 3 randomised controlled trial concerning an intervention for the early warning of COPD exacerbations, COPDPredict™, in patients with COPD. It will provide clinicians with a possible alternative for detecting COPD exacerbations early and actioning timely treatment changes to prevent unnecessary hospitalisation.
- An embedded economic and acceptability analysis will give practical information for rollout into clinical practice
- The study is limited to detecting effects on hospitalisations, and it is possible important changes in other aspects of COPD could be missed, as not powered to detect them.
- The requirement for blood CRP as an input to the prediction algorithm, and regular spirometry by patients introduces logistical challenges for staff and patients respectively

For peer review only

Background

With 65 million cases globally, chronic obstructive pulmonary disease (COPD) is the 4th leading cause of death and imposes a heavy burden on patients' lives and healthcare resources worldwide[1]. UK death rates are currently double the European Union (EU) average with over 30,000 people dying yearly and annual COPD-related NHS direct costs exceeding £800m[2]. Patients can have acute exacerbations of their COPD (AECOPD), which reduce quality of life, and lead to 140,000 emergency hospital admissions a year in the UK[3]. Indeed, AECOPD remain the 2nd commonest cause of emergency hospital admissions and 1 in 3 of these patients (sometimes more) will be readmitted within 3 months[4,5]. Hospitalisation itself carries a poor prognosis with an increased mortality risk.

Around half of all patients with COPD have frequent (≥2 per year) AECOPD[6,7]. Rapid readmissions are also common – the national COPD audit has shown that 43% of patients with COPD who were admitted are back in hospital within 90 days[8], and up to 71% by 12 months[9]. Exacerbations impact significantly on COPD outcomes, causing significant lung function decline[10]. Prompt exacerbation management optimises recovery and delays the time to the next acute episode[11], with NICE COPD guidelines highlighting a time window (prodrome) between an initial exacerbation's symptoms/signs and subsequent hospitalisation. Within this prodrome there is an opportunity to intervene. Current practice for COPD patients is that they are encouraged to recognise AECOPD, via standard self-management plans (SSMPs), and treat, using rescue medication (RM), but in many cases because of day-to-day variability in symptoms, the start of an exacerbation goes unrecognised and untreated. This inability/uncertainty to recognise and treat exacerbations in their early phase can lead to hospital admissions, which may drive long term decline.

Pharmacological and non-pharmacological treatments are used to reduce COPD exacerbations[12,13], and improve care, such as pulmonary rehabilitation (PR[14]), azithromycin[15], inhaled bronchodilators and inhaled corticosteroids. Infection control measures during the pandemic reduced spread of

For peer review only

other respiratory viruses, which in turn was associated with a 43% fall in admissions, with benefits maintained post-lockdown[16] though this is not currently part of international COPD guidelines[17, 18]. SSMPs help patients but have not really shown significant impact on A&E visits or hospital admissions as demonstrated by a systematic review of self-management strategies for COPD[19]. Intuitively early recognition and treatment of AECOPD would reduce exacerbation severity and duration, and improve prognosis; evidence for this is limited but supportive[20,21].

This study looks to address the problem by personalising and thus optimising effectiveness of AECOPD management using the CE marked and MHRA registered solution, COPDPredict™[22].

Methods

Patient and public involvement

Throughout the design and delivery of the trial public and participant involvement (PPI) has been accessed. In particular independent members on the trial steering committee provide oversight and contributions to the trial, including design and standardisation of trial-specific SSMP. PPI involvement has also extended to the qualitative research where the topic guide was developed in conjunction with respiratory participant groups.

Trial Design

The Predict & Prevent AECOPD trial is a phase III, 2 arm, multi-centre, open label, parallel-group individually randomised clinical trial investigating the use of a personalised early warning decision support system (COPDPredict™) to predict and prevent acute exacerbations of COPD. We aim to recruit 384 participants from NHS hospitals in the UK and randomise each individual in a 1:1 ratio to either SSMP with RM (control arm) or COPDPredict™ with RM (intervention arm) (*Figure 1*). The start date for the trial was 30th January 2019 and the end date for the trial is 30th March 2023.

The main outcome measure is to provide further validation, as compared to usual care, for the clinical effectiveness of COPDPredict™ to help guide and support COPD patients and their respective clinical teams in identifying exacerbations early, with an aim to reduce the total number of AECOPD-

For peer review only

induced hospital admissions in the 12 months following each patient's randomisation. The trial includes a health economic analysis examining health-related quality of life (HRQoL) using the validated questionnaire EQ-5D-5L and healthcare utilisation (as determined by a questionnaire that inquires as to hospitalisations, GP attendances and medication usage). A qualitative sub-study will assess both the end user experience and the healthcare provider experience, using Normalization Process Theory to understand the implementation and integration of the new technology[23].

Eligibility Criteria

Inclusion Criteria

- Clinically diagnosed COPD, confirmed by post-bronchodilator spirometry and defined as Post bronchodilator Forced expiratory volume/Forced vital capacity (FEV_1/FVC) <0.7 and <lower limit of normal, in the stable state
- ≥1 AECOPD in any 12-month period within the last 2 years or ≥1 hospital admission for AECOPD in the previous two years.
- Exacerbation free for at least 6 weeks
- An age of at least 18 years
- Willing and able to comply with data collection to 12 months from randomisation
- Ability to consent
- Ability to use intervention as judged by the investigator at screening, upon demonstration of the system to the patient

Exclusion Criteria

- Life expectancy <12 months -Assessment the individual investigator
- Co-enrolment into any clinical trials of investigative medicinal product (CTIMPs)
- Patients with selected co-morbidities, where these could impair use of the intervention (eg dementia)

For peer review only

Outcomes

Primary Outcome

Number of hospital admissions at 12 months post randomisation where the primary reason for admission is AECOPD. This will be obtained from participant testimony and from centrally held (HES) records. Data from the 2 sources will be cross-referenced to remove double-counting. The total number of admissions will be the sum of unique admissions from the 2 sources of data

Secondary Outcomes

Clinical Outcomes

Over a 12-month period, following randomisation;

Total in participant days, Number of visits to A&E, Number of participant defined exacerbations, Appropriate action on symptoms of AECOPD, as shown by the app, or participant testimony, Health-related quality of life (CAT) and, FEV_1 at 12 months post randomisation (from spirometry obtained during study visit)

Economic Outcomes

Health-related quality of life at baseline and 3, 6, 9 and 12-months post randomisation (EQ-5D-5L)

Healthcare utilisation at 3, 6, 9 and 12-months post randomisation, as determined by a questionnaire that inquires on hospitalisations, GP attendances and medication usage

Qualitative sub-study

End user experience and provider experience

Control Arm

A standard self-management plan (SSMP) alongside use of rescue medication (RM) containing 5 days of antibiotic and steroid treatment.

For peer review only

Intervention

COPDPredict™, consists of a patient-facing App (iOS/Android) and clinician-facing dashboard[24]. The Early Warning Decision Support System uses longitudinal remote monitoring of relevant subjective and objective data (symptoms, spirometry, biomarkers) in real-time via the App which is connected to CE-marked Bluetooth-enabled sensor peripherals. These data are used to construct COPD-relevant individual profiles such that artificial intelligence artificial intelligence (AI)-driven algorithms can then identify changes in health status to provide timely individualised alerts to patients and clinicians and sign-posting to action plans for patients.

COPDPredict™ also provides information around COPD self-management, pulmonary rehabilitation, inhaler technique and utilises gamification to help with adherence. Patients have full access to their results and can also directly message their clinical team. COPDPredict™ may signpost a participant to a self-management action plan for exacerbation management but clinical team supervision and oversight will remain throughout, as per usual care.

The clinician facing dashboard allows for "real-time" case management with the ability to remotely monitor patients and facilitate interaction. Clinicians can choose to escalate treatments based on the results being transmitted by the patients. A crucial dimension to this advanced system is that blood biomarker measurements which inform an algorithm are incorporated to enhance accuracy. Blood-based point of care (POC) analysers are used with COPDPredict™ and these POC analysers have previously been validated against laboratory CRP measures.

Recruitment and Consent

All patients must provide fully informed consent. A Participant Information Sheet (PIS) which explains the different ways in which the participant may enter the trial is provided to participants to facilitate this process. Investigators or delegate(s) adequately explain the aim, trial intervention, anticipated benefits and potential hazards of taking part in the trial to the participant. They also stress that participation is voluntary and that the participant is free to refuse to take part and may

For peer review only

withdraw from the trial at any time. Any trial specific procedures are completed after the participant has given informed consent. At each visit the participant's willingness to continue in the trial is ascertained and documented in the medical notes. Where new information becomes available which may affect the participants' decision to continue, participants will be given time to consider and if happy to continue will be re-consented. Re-consent will be documented in the medical notes.

Randomisation

After informed consent has been obtained and full participant eligibility confirmed (including exacerbation free for 6 weeks), the participant can be randomised into the trial. Randomisation CRFs are provided to investigators and may be used to collate the necessary information prior to randomisation. Participants are randomised by computer/telephone at the level of the individual in a 1:1 ratio to either SSMP with RM (control arm) or COPDPredict™ with RM (intervention arm).

A minimisation algorithm will be used within the online randomisation system to ensure balance in the treatment allocation over centre, age (<60, ≥60 years) and severity of disease as per Global Initiative for Obstructive Lung Disease (GOLD)[1].

The participants are randomised to one of the following:

<u>Control Arm</u>

A standard self-management plan (SSMP) alongside use of rescue medication (RM) containing 5 days of antibiotic and steroid treatment.

<u>Intervention arm</u>

Supported self-management using the COPDPredict™ App, involving personalised alerts to both patients and the clinical care team.

A 'random element' will be included in the minimisation algorithm, so that each participant has a probability, of being randomised to the opposite treatment that they would have otherwise

For peer review only

received. Once randomised patients will also be asked to complete baseline quality of life booklet which contains both the EQ-5D-5L and CAT questionnaire.

For peer review only

Assessment schedule

Visit	Hospital Discharge/ Initial appointment	Personal Baseline Day 1 after 6 weeks exacerbation free	Personal Baseline Day 7 (±2 days)	End of Personal Baseline Period	Approximately 4 times during any exacerbation (home visit or clinic)	Weekly until Week 52	Week 13 Telephone follow Up +/-1 week	Week 26 Hospital/ Telephone follow Up +/-1 week	Week 39 Telephone follow Up +/-1 week	Week 52 Telephone /Hospital follow Up +/- 2 weeks	Patients daily wellbeing completion post two-week bassline period
Eligibility check	X										
Valid informed consent	X										
Randomisation		X									
Height and Weight	X									X	
Concomitant medication	X	X		X				X	X	X	
Baseline medical history taken^α	X										
Request to GP for rescue medication	X						(X)	(X)	(X)		
Assessment of (S)AEs	X				(X)		X	X	X	X	

Questionnaires (QoL and health utilisation)	X						X	X	X	X	
Spirometry	X							X		X	
Intervention only below this line					Intervention only below this line						
Provision of equipment & participant training		X									
Spirometry	X	X	X		(X)	X					
Symptom control markers	X	X	X	X	(X)	X	X	X	X	X	
Point of care blood test		X		X	(X)						
Clinical histories		X		X			X	X	X	X	
Wellbeing (App)											X

(X) denotes only when required. If rescue medication has not been used, fresh prescription requests will not be necessary

During the baseline period and any subsequent exacerbations patients will be entering well-being data on the App which will be related to lifestyle choices and the end-user experience. This data will not form part of the formal analysis of quality of life.

Table 1: Schedule of assessments

For peer review only

For peer review only - http://bmjopen.bmj.com/site/about/guidelines.xhtml

Statistical Analysis

Sample size

The justification of the sample size is based on previous evidence[25] that had shown a mean estimate of 2.5 COPD admissions in the previous year in the control group. To detect a difference of 1 admission in the mean number of admissions between groups using the standard methods of difference between means and assuming standard deviation of 2.6 with 90% power and a type I error rate of 5% (two-sided), 144 participants per group will need to be randomised, 288 in total. Assuming and adjusting for a 25% loss to follow-up/ drop-out rate, 384 participants will need to be recruited.

Analysis of Outcome Measures

The primary comparison groups will be composed of those randomised to the use of COPDPredict™ system and those randomised to the standard self-management plan (SSMP). All analyses will be based on the intention to treat principle[26], with all patients analysed in the treatment groups to which they were allocated irrespective of compliance with the randomised allocated treatment, and all patients will be included in the analyses. For all outcomes, summary statistics (e.g. mean differences, relative risks) will be reported and 95% confidence intervals will be constructed where appropriate. A two-sided p-value of <0.05 will be considered statistically significant.

Primary Outcome Measure

The primary analysis for this study will be to compare rates of hospital admission between the treatment groups (COPDPredict™ versus Usual care) over 12 months, following randomisation, where the primary reason for admission is an AECOPD event. These hospital admissions per person will be analysed using Poisson regression models adjusting for treatment group and minimisation variables. If there is over dispersion a negative binomial regression model adjusting for treatment group and the same minimisation variables will be taken into consideration. Point estimates

For peer review only

(incidence rates) will be provided and accompanied with 95% confidence intervals and respective p-values.

Secondary Outcome Measures

Recorded observations taken over a 12-month period, following randomisation:

Total in-hospital days and FEV_1 – These variables will be summarised using basic descriptive statistics (Mean, SD)). We may also consider a mixed linear regression model, to estimate differences between the intervention group supported with 95% CI adjusting for baseline variables and minimisation variables (centre being a random effects variable).

Health-related quality of life questionnaires - EQ5D-5L and CAT will be converted into scores and analysed using a mixed linear regression model, adjusting for the intervention group, baseline and minimisation variables as before.

Number of A&E visits, participant defined exacerbations and healthcare utilisation - Where the participant experienced an episode, the data during the 12 months from randomisation will be analysed using mixed effects log-binomial regression techniques with presentation of relative risk and 95% confidence intervals. Furthermore, sensitivity analysis based on patients' experience of multiple visits to A&E and participant defined exacerbation will be calculated using Poisson regression techniques and relative risk supported with 95% CI.

Symptom control marker association to clinical decision - The diagnostic accuracy of COPDPredict™ and Usual care will be evaluated by calculating sensitivity, specificity, positive and negative predictive values, and area under curve (AUC)/receiver operating characteristics (ROC) together with 95% CI.

Subgroup analyses

Subgroup analyses will be limited to the same variables used in the minimisation algorithm, apart from centre. Tests for statistical heterogeneity (e.g. by including the treatment group by subgroup

For peer review only - http://bmjopen.bmj.com/site/about/guidelines.xhtml

interaction parameter in the statistical model) will be performed prior to any examination of effect estimate within subgroups. The results of subgroup analyses will be treated with caution and will be used for the purposes of hypothesis generation only.

Missing data and sensitivity analyses

Participants with missing primary outcome data will not be included in the primary analysis in the first instance. This presents a risk of bias, and sensitivity analyses will be undertaken to assess the possible impact. This will consist of simulating the missing response using a multiple imputation approach[27]. Parameters used to simulate the missing response will include the minimisation variables, intervention group and previous response. Full details are included in the statistical analysis plan (SAP).

Adverse events and serious adverse events

Patients with COPD can have high disease burden, such that the trial population will be older, with co-morbidities such as osteoporosis[28], cardiovascular disease or raised cardiovascular risk[29], along with associated complications and symptoms including abnormal lab results. A relatively high number of adverse events are anticipated as a result of the patients' existing past medical history[30]. However, this study is examining the role of increased participant surveillance and therefore few, if any, foreseeable risks of direct harm associated with the study intervention are anticipated. AE reporting will therefore be limited to those events which are required for trial monitoring or outcome assessment.

All events which meet the definition of serious must be recorded in the participant notes, but for trial purposes these following events do not require reporting on the SAE Form. Such events are "safety reporting exempt".

• Planned hospitalisation for a pre-existing condition, or a procedure required by the trial protocol without serious deterioration in health, is not considered a serious adverse event.

For peer review only - http://bmjopen.bmj.com/site/about/guidelines.xhtml

• An overnight stay in hospital that is due to transportation, organisational or accommodation problems, and without medical background SAES requiring expedited reporting

Any other events that fulfil the usual definition of an SAE will be reported immediately.

Any death occurring during the Trial Protocol defined follow up period (12 months) must be reported as an SAE within 24 hours of the local investigator becoming aware of the event.

Health economics

Within -trial analysis

The economic evaluation alongside the trial will take the form of an incremental cost-effectiveness analysis to estimate cost per hospital admission avoided and incremental cost-utility analysis to estimate cost per quality adjusted life year (QALY) gained. Base-case analyses will be from a healthcare perspective over 12 months follow-up using individual participant level data on costs and outcomes from the clinical trial. Additional analyses will be undertaken from a broader societal perspective. Resource use data will be collected by questionnaire, capturing information on hospitalisations for exacerbations, COPD-related primary and secondary care attendances and medication usage. Data will also be collected during the trial on resource use implications of the intervention. Information on participant incurred costs, time off work and impact on activities is collected within the economic questionnaire. QALYs will be calculated from EQ-5D-5L responses. Sensitivity analysis will be undertaken and cost-effectiveness planes and cost-effectiveness acceptability curves presented.

Model-based analysis

A decision model will be constructed to extrapolate beyond trial results over five years, with costs and benefits discounted at 3.5%. The model will be the first decision analysis to use GOLD stages ABCD[1] and will consider the ability of the COPDPredict™ supported self-management to impact movements between GOLD ABCD stages, but also the number of exacerbations within those stages.

For peer review only

Costs associated with delivering COPDPredict™, exacerbations, and routine care will be obtained from the trial. Quality of life scores on each intervention will also be obtained from trial data using EQ-5D-5L. Longer-term costs, quality of life scores, and transitions between stages, and number of exacerbations will be obtained from analysis of ABCD cohorts from within the BLISS[2] study, a prospective cohort study with 3-year follow-up period. The model will estimate cost per QALY gained of COPDPredict™ supported self-management versus standard self-management from a healthcare perspective. Probabilistic sensitivity analysis will be undertaken to simultaneously incorporate all parameter uncertainty. Cost-effectiveness planes and cost-effectiveness acceptability curves will be presented to show the probability the intervention is cost-effective at different cost/QALY thresholds. A value of information analysis will assess the value of further trials to reduce parameter uncertainty and identify parameters which would be most valuable in reducing that uncertainty[31].

Qualitative sub-study

In order to frame this study, we draw on Normalisation Process Theory[32], which proposes four constructs essential to effect implementation of new technologies: *coherence* (sense making work to understand the possibilities of a new technology), *cognitive participation* (relational work as new technology begins to be used), *collective action* (operational work enacted to make a technology function in context) and *reflexive monitoring* (the appraisal of work that people do to assess how the new technology affects them and others). Participants will include health care professionals, patients and wider policy stakeholders and will be purposively sampled to maximise diversity. A topic guide developed by drawing on existing literature and theory will focus on attitudes to and practices around self-management of COPD and the use and implementation of digital technologies. Interviews will be audio recorded and transcribed verbatim, before subjecting the data to a two-stage approach to analysis[33]: first, a descriptive framework analysis[34] followed by mapping onto the NPT constructs. We anticipate conducting approximately30 interviews but this will be informed by the data and analysis35].

For peer review only

Ethics and dissemination:

This study protocol is reported in accordance with the guidance set out in the SPIRIT statement. Predict and Prevent AECOPD has obtained ethical approval in England (19/LO/1939). Upon completion of the trial and publication of results a lay findings summary will be disseminated to trial participants.

Trial Oversight

Trial oversight will be provided by the TMG, an independent DMC and independent TSC. Interim analyses of major outcome measures and safety data will be conducted and provided in strict confidence to the DMC.

Potential Impact

Predict & Prevent AECOPD is a pragmatic, national, multicentre, RCT which aims to provide robust evidence around the clinical and cost effectiveness of COPDPredict™ by analysing the number of patients admitted to hospital in a 12month period post randomisation. It is hoped the outcome of this trial could be used to provide robust evidence to the management of patients with COPD and their exacerbations in the future.

Contributorship statement

All authors have made substantial contributions to the conception or design of the work, or the acquisition, analysis or interpretation of data. DK, AT, HJ, RM, SJ, NG, NP and MS drafted the manuscript and revised it critically for important intellectual content. All authors approved final version and agreed to be accountable for all aspects of the work in ensuring that questions related to the accuracy or integrity of any part of the work are appropriately investigated and resolved.

Competing Interest Statement

The CI of the trial (AT) does not have any relevant direct financial disclosures, nor do members of the trial management group (TMG) who are authors of this paper, with the exception of NP who is a

For peer review only

founder, director and share-holder of NEPeSMO, who developed the intervention. In addition, MS is CI of the overall NIHR-funded project and is a founder, director and share-holder of NEPeSMO. NEPeSMO is a start-up company from the University Hospitals of North Midlands NHS Trust, owns all intellectual property rights of COPDPredict™ and is a project collaborator on the grant.

AT has grants from pharmaceutical companies working in the area of COPD (Chiesi, AstraZeneca) and has conducted advisory work for such (Boehringer, CSL Behring) but not in the area of medical devices or admission prevention. Neither has she worked for, or received monies from, any company working on admission prevention in the last 3 years.

Funding Statement & Disclaimer

This report is independent research funded by the National Institute for Health Research (Invention for Innovation, A Personalised Early Warning Decision Support System with novel Saliva Bio-Profiling to Predict and Prevent Acute Exacerbations of Chronic Obstructive Pulmonary Disease – 'Predict&prevent AECOPD, NIHR200002).

For peer review only

References

1. Global Initiative for Obstructive Lung Disease, [Accessed 13 June 2018]. [Available from: www.goldcopd.com].

2. Estimating the economic burden of respiratory illness in the UK. London: British Lung Foundation 2016.

3. The battle for breath - the impact of lung disease in the UK. London: British Lung Foundation, 2016.

4. Roberts CM, Stone RA, Lowe D, Pursey NA, Buckingham RJ. Co-morbidities and 90-day outcomes in hospitalized COPD exacerbations. Copd. 2011;8(5):354-61.

5. Stone RA HB, Lowe D, Searle L, Skipper E, Welham S, et al. . National COPD audit programme. COPD: Who cares matters. London, UK: Royal College of Physicians, 2015. Contract No.: eISBN 978-1-86016-559-7.

6. Hurst JR, Vestbo J, Anzueto A, Locantore N, Mullerova H, Tal-Singer R, et al. Susceptibility to exacerbation in chronic obstructive pulmonary disease. N Engl J Med. 2010;363(12):1128-38.

7. Merinopoulou E, Raluy-Callado M, Ramagopalan S, MacLachlan S, Khalid JM. COPD exacerbations by disease severity in England. Int J Chron Obstruct Pulmon Dis. 2016;11:697-709.

8. Stone RA HB, McMillan V, Saleem Khan M, Searle L, Skipper E, et al. COPD: Who cares when it matters most? London: Royal College of Physicians, 2017.

9. Steer J, Gibson GJ, Bourke SC. Longitudinal change in quality of life following hospitalisation for acute exacerbations of COPD. BMJ Open Respir Res. 2015;2(1):e000069.

10. Jo YS, Kim SK, Park SJ, Um S-J, Park Y-B, Jung KS, et al. Longitudinal change of FEV(1) and inspiratory capacity: clinical implication and relevance to exacerbation risk in patients with COPD. Int J Chron Obstruct Pulmon Dis. 2019;14:361-9.

For peer review only

11. Qureshi H SA, Hanania NA. Chronic obstructive pulmonary disease exacerbations: latest evidence and clinical implications. Therapeutic advances in chronic disease. 2014;5(5):212-27. Epub 2014/09/02.

12. Oba, Y., Keeney, E., Ghatehorde, N. and Dias, S., 2018. Dual combination therapy versus long-acting bronchodilators alone for chronic obstructive pulmonary disease (COPD): a systematic review and network meta-analysis. Cochrane Database of Systematic Reviews, [online] 2018(12). Available at: <https://www.cochranelibrary.com/cdsr/doi/10.1002/14651858.CD012620.pub2/full> [Accessed 5 October 2021].

13. Yang, I., Clarke, M., Sim, E. and Fong, K., 2012. Inhaled corticosteroids for stable chronic obstructive pulmonary disease. Cochrane Database of Systematic Reviews, [online] Available at: <http://Inhaled corticosteroids for stable chronic obstructive pulmonary disease> [Accessed 1 September 2021].

14. Ryrso CK, Godtfredsen NS, Kofod LM, Lavesen M, Mogensen L, Tobberup R, et al. Lower mortality after early supervised pulmonary rehabilitation following COPD-exacerbations: a systematic review and meta-analysis. BMC Pulm Med. 2018;18(1):154.

15. Ni, W., Shao, X., Cai, X., Wei, C., Cui, J., Wang, R. and Liu, Y., 2015. Prophylactic Use of Macrolide Antibiotics for the Prevention of Chronic Obstructive Pulmonary Disease Exacerbation: A Meta-Analysis. PLOS ONE, 10(3), p.e0121257.

16. Lawless, M., Burgess, M. and Bourke, S., 2022. Impact of COVID-19 on Hospital Admissions for COPD Exacerbation: Lessons for Future Care. Medicina, 58(1), p.66.

17. Nice.org.uk. 2018. Overview | Chronic obstructive pulmonary disease in over 16s: diagnosis and management | Guidance | NICE. [online] Available at: <https://www.nice.org.uk/guidance/ng115> [Accessed 23 July 2022].

18. Global Initiative for Chronic Obstructive Lung Disease - GOLD. 2022. 2022 GOLD Reports - Global Initiative for Chronic Obstructive Lung Disease - GOLD. [online] Available at: <https://goldcopd.org/2022-gold-reports-2/> [Accessed 23 July 2022].

19. Jolly K, Majothi S, Sitch AJ, Heneghan NR, Riley RD, Moore DJ, et al. Self-management of health care behaviors for COPD: a systematic review and meta-analysis. Int J Chron Obstruct Pulmon Dis. 2016;11:305-26.

20. Vijayasaratha K, Stockley RA. Relationship between frequency, length, and treatment outcome of exacerbations to baseline lung function and lung density in alpha-1 antitrypsin-deficient COPD. Int J Chron Obstruct Pulmon Dis. 2012;7:789-96.

21. Celli BR, Locantore N, Yates J, Tal-Singer R, Miller BE, Bakke P, et al. Inflammatory biomarkers improve clinical prediction of mortality in chronic obstructive pulmonary disease. Am J Respir Crit Care Med. 2012;185(10):1065-72.

For peer review only

For peer review only - http://bmjopen.bmj.com/site/about/guidelines.xhtml

22. Patel N, Kinmond K, Jones P, Birks P, Spiteri MA. Validation of COPDPredict™: Unique Combination of Remote Monitoring and Exacerbation Prediction to Support Preventative Management of COPD Exacerbations. International Journal of Chronic Obstructive Pulmonary Disease 2021, 16:1887-1899

23. May CR, Mair F, Finch T, MacFarlane A, Dowrick C, Treweek S, Rapley T, Ballini L, Ong BN, Rogers A, Murray E. Development of a theory of implementation and integration: Normalization Process Theory. Implementation Science. 2009 Dec;4(1):1-9.

24. Nepesmo.com. 2017. NEPeSMO – Predict to Prevent. [online] Available at: <https://nepesmo.com/#:~:text=NEPeSMO%20offers%20a%20smart%20CE,to%20patients%20living%20with%20COPD> [Accessed 16 June 2021].

25. Pinnock H, Hanley J, McCloughan L, Todd A, Krishan A, Lewis S, et al. Effectiveness of telemonitoring integrated into existing clinical services on hospital admission for exacerbation of chronic obstructive pulmonary disease: researcher blind, multicentre, randomised controlled trial. Bmj. 2013;347:f6070

26. Sandeep K Gupta. Intention-to-treat concept: A Review. Perspect Clin Res 2011:2(3);109-112

27 . White IR, Royston P, Wood AM. Multiple imputation using chained equations. Stats in Medicine 2011, (30) 377-399.

28. Vrieze A, de Greef MH, Wijkstra PJ, Wempe JB. Low bone mineral density in COPD patients related to worse lung function, low weight and decreased fat-free mass. Osteoporos Int. 2007;18(9):1197-202.

29. Patel AR, Kowlessar BS, Donaldson GC, Mackay AJ, Singh R, George SN, et al. Cardiovascular risk, myocardial injury, and exacerbations of chronic obstructive pulmonary disease. Am J Respir Crit Care Med. 2013;188(9):1091-9.

30. Miller J, Edwards LD, Agusti A, Bakke P, Calverley PM, Celli B, et al. Comorbidity, systemic inflammation and outcomes in the ECLIPSE cohort. Respir Med. 2013;107(9):1376-84.

31. Global Initiative for Obstructive Lung Disease COPD Diagnosis and Management At-A-Glance Desk Reference 2017 [online] https://goldcopd.org/archived-reports/ Accessed 03/09/21

32. May C, Finch T. Implementing, Embedding, and Integrating Practices: An Outline of Normalization Process Theory. Sociology. 2009;43(3):535-54.

33. Ritchie J, Spencer L. Qualitative data analysis for applied policy research. In: Bryman A, Burgess RG, editors. London: Routledge; 1994.

34. Macfarlane A, O'Reilly-de Brún M. Using a theory-driven conceptual framework in qualitative health research. Qual Health Res. 2012;22(5):607-18.

For peer review only

35. O'Cathain A, Hoddinott P, Lewin S, Thomas KJ, Young B, Adamson J, et al. Maximising the impact of qualitative research in feasibility studies for randomised controlled trials: guidance for researchers. Pilot and Feasibility Studies. 2015;1(1):32.

Figure Legends

Figure 1: Flow diagram of the Predict & Prevent trial

Table Legends

Table 1: Schedule of assessments

For peer review only

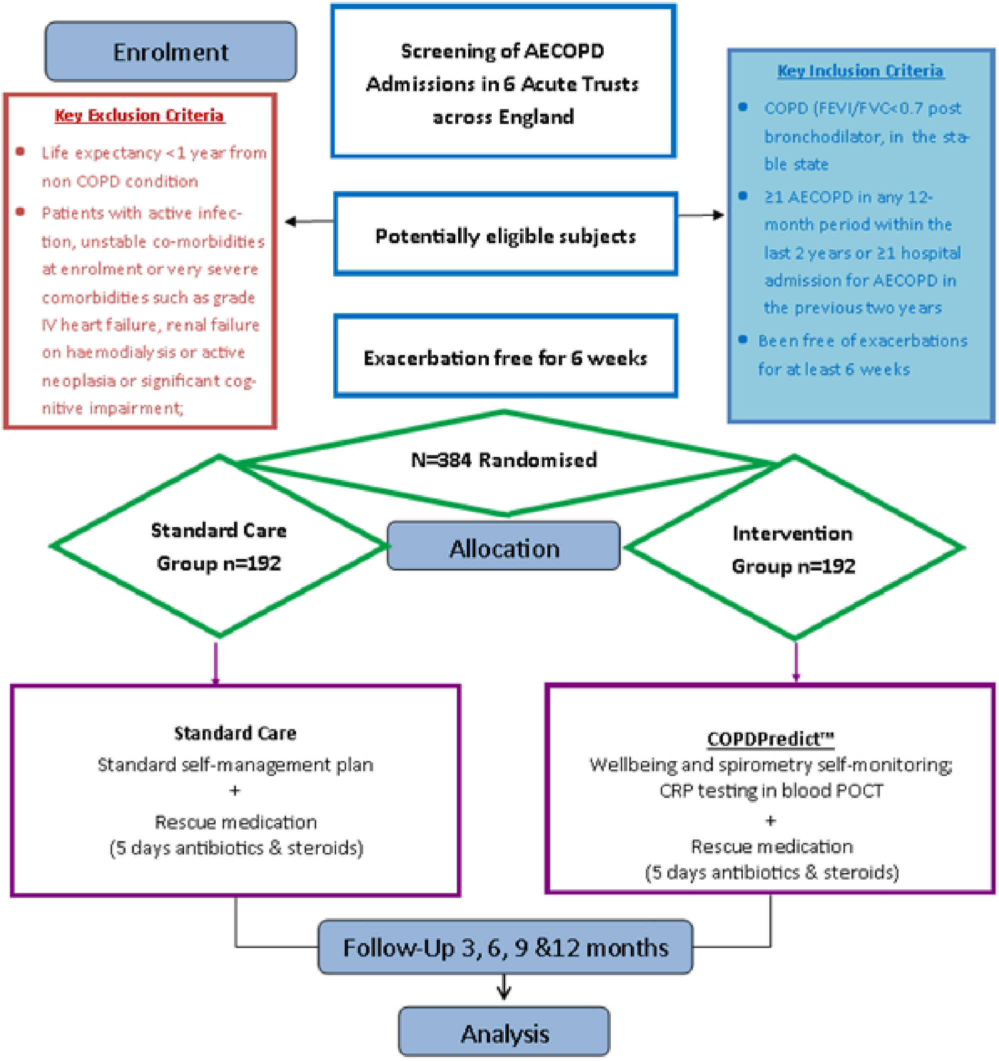

Figure 1: Flow diagram of the Predict & Prevent trial

266x297mm (57 x 57 DPI)

SPIRIT

STANDARD PROTOCOL ITEMS: RECOMMENDATIONS FOR INTERVENTIONAL TRIALS

SPIRIT 2013 Checklist: Recommended items to address in a clinical trial protocol and related documents*

Section/item	ItemNo	Description	Section of manuscript
**Administrative information**			
Title	1	Descriptive title identifying the study design, population, interventions, and, if applicable, trial acronym	Page 5 methods
Trial registration	2a	Trial identifier and registry name. If not yet registered, name of intended registry	Page 2 abstract
	2b	All items from the World Health Organization Trial Registration Data Set	
Protocol version	3	Date and version identifier	V9.0 vd03-Mar-22
Funding	4	Sources and types of financial, material, and other support	Page 2 NIHR Statement
Roles and responsibilities	5a	Names, affiliations, and roles of protocol contributors	Page 1
	5b	Name and contact information for the trial sponsor	Page 1 and 2
	5c	Role of study sponsor and funders, if any, in study design; collection, management, analysis, and interpretation of data; writing of the report; and the decision to submit the report for publication, including whether they will have ultimate authority over any of these activities	

For peer review only

	5d	Composition, roles, and responsibilities of the coordinating centre, steering committee, endpoint adjudication committee, data management team, and other individuals or groups overseeing the trial, if applicable (see Item 21a for data monitoring committee)	
**Introduction**			
Background and rationale	6a	Description of research question and justification for undertaking the trial, including summary of relevant studies (published and unpublished) examining benefits and harms for each intervention	Page 4-5
	6b	Explanation for choice of comparators	
Objectives	7	Specific objectives or hypotheses	Page 5
Trial design	8	Description of trial design including type of trial (eg, parallel group, crossover, factorial, single group), allocation ratio, and framework (eg, superiority, equivalence, noninferiority, exploratory)	Page 5-6
**Methods: Participants, interventions, and outcomes**			
Study setting	9	Description of study settings (eg, community clinic, academic hospital) and list of countries where data will be collected. Reference to where list of study sites can be obtained	Page 5

For peer review only

Eligibility criteria	10	Inclusion and exclusion criteria for participants. If applicable, eligibility criteria for study centres and individuals who will perform the interventions (eg, surgeons, psychotherapists)	Page 6
Interventions	11a	Interventions for each group with sufficient detail to allow replication, including how and when they will be administered	Page 7
	11b	Criteria for discontinuing or modifying allocated interventions for a given trial participant (eg, drug dose change in response to harms, participant request, or improving/worsening disease)	N/A
	11c	Strategies to improve adherence to intervention protocols, and any procedures for monitoring adherence (eg, drug tablet return, laboratory tests)	N/A
	11d	Relevant concomitant care and interventions that are permitted or prohibited during the trial	N/A
Outcomes	12	Primary, secondary, and other outcomes, including the specific measurement variable (eg, systolic blood pressure), analysis metric (eg, change from baseline, final value, time to event), method of aggregation (eg, median, proportion), and time point for each outcome. Explanation of the clinical relevance of chosen efficacy and harm outcomes is strongly recommended	Page 6/7

Participant timeline	13	Time schedule of enrolment, interventions (including any run-ins and washouts), assessments, and visits for participants. A schematic diagram is highly recommended (see Figure)	Page 10
Sample size	14	Estimated number of participants needed to achieve study objectives and how it was determined, including clinical and statistical assumptions supporting any sample size calculations	Page 5
Recruitment	15	Strategies for achieving adequate participant enrolment to reach target sample size	Page 8

Methods: Assignment of interventions (for controlled trials)

Allocation:

Sequence generation	16a	Method of generating the allocation sequence (eg, computer-generated random numbers), and list of any factors for stratification. To reduce predictability of a random sequence, details of any planned restriction (eg, blocking) should be provided in a separate document that is unavailable to those who enrol participants or assign interventions	Page 8 and 9
Allocation concealment mechanism	16b	Mechanism of implementing the allocation sequence (eg, central telephone; sequentially numbered, opaque, sealed envelopes), describing any steps to conceal the sequence until interventions are assigned	Protocol

Implementation	16c	Who will generate the allocation sequence, who will enrol participants, and who will assign participants to interventions	Protocol
Blinding (masking)	17a	Who will be blinded after assignment to interventions (eg, trial participants, care providers, outcome assessors, data analysts), and how	N/A
	17b	If blinded, circumstances under which unblinding is permissible, and procedure for revealing a participant's allocated intervention during the trial	
**Methods: Data collection, management, and analysis**			
Data collection methods	18a	Plans for assessment and collection of outcome, baseline, and other trial data, including any related processes to promote data quality (eg, duplicate measurements, training of assessors) and a description of study instruments (eg, questionnaires, laboratory tests) along with their reliability and validity, if known. Reference to where data collection forms can be found, if not in the protocol	Page 12-16 and Protocol
	18b	Plans to promote participant retention and complete follow-up, including list of any outcome data to be collected for participants who discontinue or deviate from intervention protocols	Page 12-13

Data management	19	Plans for data entry, coding, security, and storage, including any related processes to promote data quality (eg, double data entry; range checks for data values). Reference to where details of data management procedures can be found, if not in the protocol	Protocol
Statistical methods	20a	Statistical methods for analysing primary and secondary outcomes. Reference to where other details of the statistical analysis plan can be found, if not in the protocol	Protocol
	20b	Methods for any additional analyses (eg, subgroup and adjusted analyses)	Page 15-16
	20c	Definition of analysis population relating to protocol non-adherence (eg, as randomised analysis), and any statistical methods to handle missing data (eg, multiple imputation)	Page 14
**Methods: Monitoring**			
Data monitoring	21a	Composition of data monitoring committee (DMC); summary of its role and reporting structure; statement of whether it is independent from the sponsor and competing interests; and reference to where further details about its charter can be found, if not in the protocol. Alternatively, an explanation of why a DMC is not needed	Protocol

	21b	Description of any interim analyses and stopping guidelines, including who will have access to these interim results and make the final decision to terminate the trial	Protocol
Harms	22	Plans for collecting, assessing, reporting, and managing solicited and spontaneously reported adverse events and other unintended effects of trial interventions or trial conduct	Protocol
Auditing	23	Frequency and procedures for auditing trial conduct, if any, and whether the process will be independent from investigators and the sponsor	
**Ethics and dissemination**			
Research ethics approval	24	Plans for seeking research ethics committee/institutional review board (REC/IRB) approval	Page 16
Protocol amendments	25	Plans for communicating important protocol modifications (eg, changes to eligibility criteria, outcomes, analyses) to relevant parties (eg, investigators, REC/IRBs, trial participants, trial registries, journals, regulators)	Protocol
Consent or assent	26a	Who will obtain informed consent or assent from potential trial participants or authorised surrogates, and how (see Item 32)	Protocol
	26b	Additional consent provisions for collection and use of participant data and biological specimens in ancillary studies, if applicable	

For peer review only

Confidentiality	27	How personal information about potential and enrolled participants will be collected, shared, and maintained in order to protect confidentiality before, during, and after the trial	Protocol
Declaration of interests	28	Financial and other competing interests for principal investigators for the overall trial and each study site	Page 17
Access to data	29	Statement of who will have access to the final trial dataset, and disclosure of contractual agreements that limit such access for investigators	Protocol
Ancillary and post-trial care	30	Provisions, if any, for ancillary and post-trial care, and for compensation to those who suffer harm from trial participation	N/A
Dissemination policy	31a	Plans for investigators and sponsor to communicate trial results to participants, healthcare professionals, the public, and other relevant groups (eg, via publication, reporting in results databases, or other data sharing arrangements), including any publication restrictions	Page 16
	31b	Authorship eligibility guidelines and any intended use of professional writers	
	31c	Plans, if any, for granting public access to the full protocol, participant-level dataset, and statistical code	N/A

Appendices

For peer review only - http://bmjopen.bmj.com/site/about/guidelines.xhtml

Informed consent materials	32	Model consent form and other related documentation given to participants and authorised surrogates	ICF
Biological specimens	33	Plans for collection, laboratory evaluation, and storage of biological specimens for genetic or molecular analysis in the current trial and for future use in ancillary studies, if applicable	N/A

*It is strongly recommended that this checklist be read in conjunction with the SPIRIT 2013 Explanation & Elaboration for important clarification on the items. Amendments to the protocol should be tracked and dated. The SPIRIT checklist is copyrighted by the SPIRIT Group under the Creative Commons "Attribution-NonCommercial-NoDerivs 3.0 Unported" license.

For peer review only

BMJ Open

A phase III, 2 arm, multi-centre, open label, parallel-group randomised designed clinical investigation of the use of a personalised early warning decision support system to predict and prevent acute exacerbations of Chronic Obstructive Pulmonary Disease - 'Predict & Prevent AECOPD' -Study Protocol

Journal:	*BMJ Open*
Manuscript ID	bmjopen-2022-061050.R4
Article Type:	Protocol
Date Submitted by the Author:	22-Nov-2022
Complete List of Authors:	Kaur, Dalbir; University of Warwick<br>Mehta, Rajnikant ; University of birmingham<br>Jarrett, Hugh; University of Birmingham<br>Jowett, Sue; University of Birmingham, Health Economics Unit<br>Gale, Nicola K.; University of Birmingham<br>Turner, Alice; University of Birmingham, Institute of Inflammation & Aging; Heart of England NHS Foundation Trust<br>Spiteri, Monica; University Hospitals of North Midlands NHS Trust<br>Patel, Neil; NEPESMO
**Primary Subject Heading**:	Respiratory medicine
Secondary Subject Heading:	Smoking and tobacco
Keywords:	RESPIRATORY MEDICINE (see Thoracic Medicine), Chronic airways disease < THORACIC MEDICINE, Thoracic medicine < INTERNAL MEDICINE

SCHOLARONE™
Manuscripts

A phase III, 2 arm, multi-centre, open label, parallel-group randomised designed clinical investigation of the use of a personalised early warning decision support system to predict and prevent acute exacerbations of Chronic Obstructive Pulmonary Disease - 'Predict & Prevent AECOPD' -Study Protocol

References: 35
Abstract count: 275
Word count: 3905

D Kaur, R Mehta, H Jarrett, S Jowett, N Gale, AM Turner, MA Spiteri, N Patel

Dalbir Kaur[1] Corresponding author
Email: dalbir.kaur@warwick.ac.uk

Rajnikant Mehta[2]
Email: R.L.Mehta@bham.ac.uk

Hugh Jarrett[3]
Email: h.jarrett@bham.ac.uk

Sue Jowett[4]
Email: S.JOWETT@bham.ac.uk

Nicola Gale[5]
Email: N.Gale@bham.ac.uk

Alice M Turner[6]
Email: A.M.Turner@bham.ac.uk

Monica A Spiteri[7]
Email: Monica.Spiteri@uhnm.nhs.uk

Neil Patel[8]
Email: neil.patel@nepesmo.com

[1]Senior Project Manager, Warwick Clinical Trials Unit (BWCTU), Warwick Medical School University of Warwick Coventry, CV4 7ALInstitute

[2]Senior Medical Statistician, Birmingham Clinical Trials Unit (BCTU), Institute of Applied Health Research, College of Medical and Dental Sciences, University of Birmingham, Edgbaston, Birmingham, B15 2TT

[3]Trials Management Team Leader, Birmingham Clinical Trials Unit (BCTU), Institute of Applied Health Research, College of Medical and Dental Sciences, University of Birmingham, Edgbaston, Birmingham, B15 2TT

[4]Professor of Health Economics, Health Economics Unit, Institute of Applied Health Research, College of Medical and Dental Sciences, University of Birmingham, Edgbaston, Birmingham, B15 2TT

For peer review only

[5]Professor of Health Policy and Sociology, Health Services Management Centre, School of Social Policy Director of Postgraduate Research, College of Social Sciences, University of Birmingham, Edgbaston, Birmingham, B15 2TT

[6] Professor of Respiratory Medicine and Honorary Consultant Respiratory Physician, Institute for Applied Health Research, University of Birmingham, Birmingham, UK, B15 2TT

[7]Director of Respiratory Research, Academic Research Unit, Courtyard Annexe - C Block, Royal Stoke University Hospital, University Hospitals of North Midlands NHS Trust, Newcastle Road, Staffordshire, UK, ST4 6QG

[8]Chief Medical Officer, NEPESMO Ltd, 76 King Street, Manchester, M2 4NH, United Kingdom
Word count: 3487 (excluding title page, abstract, table, legend and references)

For peer review only

Abstract

Introduction

With 65 million cases globally, chronic obstructive pulmonary disease (COPD) is the 4th leading cause of death and imposes a heavy burden on patients' lives and healthcare resources worldwide. Around half of all patients with COPD have frequent (≥2 per year) AECOPD[5]. Rapid readmissions are also common. Exacerbations impact significantly on COPD outcomes, causing significant lung function decline. Prompt exacerbation management optimises recovery and delays the time to the next acute episode.

Methods/analysis

The Predict & Prevent AECOPD trial is a phase III, 2 arm, multi-centre, open label, parallel-group individually randomised clinical trial investigating the use of a personalised early warning decision support system (COPDPredict™) to predict and prevent acute exacerbations of COPD. We aim to recruit 384 participants and randomise each individual in a 1:1 ratio to either standard self-management plans (SSMP) with Rescue Medication (RM) (control arm) or COPDPredict™ with RM (intervention arm)

The trial will inform the future standard of care regarding management of exacerbations in COPD patients. The main outcome measure is to provide further validation, as compared to usual care, for the clinical effectiveness of COPDPredict™ to help guide and support COPD patients and their respective clinical teams in identifying exacerbations early, with an aim to reduce the total number of AECOPD-induced hospital admissions in the 12 months following each patient's randomisation.

Ethics and Dissemination

This study protocol is reported in accordance with the guidance set out in the SPIRIT statement. Predict and Prevent AECOPD has obtained ethical approval in England (19/LO/1939). Upon completion of the trial and publication of results a lay findings summary will be disseminated to trial participants.

Trials Registration: Clinical trials.gov NCT04136418

Keywords: Respiratory, COPD, Exacerbations, Symptom Control Markers, COPDPredict™

NIHR Statement

This work is supported by the National Institute for Health Research (NIHR) under its Invention for Innovation (i4i) Programme (Grant Reference Number NIHR200002). The views expressed are those of the author(s) and not necessarily those of the NHS, the NIHR or the Department of Health.

Strengths and Limitations

- This study will be conducted as a phase 3 randomised controlled trial concerning an intervention for the early warning of COPD exacerbations, COPDPredict™, in patients with COPD. It will provide clinicians with a possible alternative for detecting COPD exacerbations early and actioning timely treatment changes to prevent unnecessary hospitalisation.
- An embedded economic and acceptability analysis will give practical information for rollout into clinical practice
- The study is limited to detecting effects on hospitalisations, and it is possible important changes in other aspects of COPD could be missed, as not powered to detect them.
- The requirement for blood CRP as an input to the prediction algorithm, and regular spirometry by patients introduces logistical challenges for staff and patients respectively

For peer review only

Background

With 65 million cases globally, chronic obstructive pulmonary disease (COPD) is the 4th leading cause of death and imposes a heavy burden on patients' lives and healthcare resources worldwide[1]. UK death rates are currently double the European Union (EU) average with over 30,000 people dying yearly and annual COPD-related NHS direct costs exceeding £800m[2]. Patients can have acute exacerbations of their COPD (AECOPD), which reduce quality of life, and lead to 140,000 emergency hospital admissions a year in the UK[3]. Indeed, AECOPD remain the 2nd commonest cause of emergency hospital admissions and 1 in 3 of these patients (sometimes more) will be readmitted within 3 months[4,5]. Hospitalisation itself carries a poor prognosis with an increased mortality risk.

Around half of all patients with COPD have frequent (≥2 per year) AECOPD[6,7]. Rapid readmissions are also common – the national COPD audit has shown that 43% of patients with COPD who were admitted are back in hospital within 90 days[8], and up to 71% by 12 months[9]. Exacerbations impact significantly on COPD outcomes, causing significant lung function decline[10]. Prompt exacerbation management optimises recovery and delays the time to the next acute episode[11], with NICE COPD guidelines highlighting a time window (prodrome) between an initial exacerbation's symptoms/signs and subsequent hospitalisation. Within this prodrome there is an opportunity to intervene. Current practice for COPD patients is that they are encouraged to recognise AECOPD, via standard self-management plans (SSMPs), and treat, using rescue medication (RM), but in many cases because of day-to-day variability in symptoms, the start of an exacerbation goes unrecognised and untreated. This inability/uncertainty to recognise and treat exacerbations in their early phase can lead to hospital admissions, which may drive long term decline.

Pharmacological and non-pharmacological treatments are used to reduce COPD exacerbations[12,13], and improve care, such as pulmonary rehabilitation (PR[14]), azithromycin[15], inhaled bronchodilators and inhaled corticosteroids. Infection control measures during the pandemic reduced spread of

For peer review only

other respiratory viruses, which in turn was associated with a 43% fall in admissions, with benefits maintained post-lockdown[16] though this is not currently part of international COPD guidelines[17, 18]. SSMPs help patients but have not really shown significant impact on A&E visits or hospital admissions as demonstrated by a systematic review of self-management strategies for COPD[19]. Intuitively early recognition and treatment of AECOPD would reduce exacerbation severity and duration, and improve prognosis; evidence for this is limited but supportive[20,21].

This study looks to address the problem by personalising and thus optimising effectiveness of AECOPD management using the CE marked and MHRA registered solution, COPDPredict™[22].

Methods

Patient and public involvement

Throughout the design and delivery of the trial public and participant involvement (PPI) has been accessed. In particular independent members on the trial steering committee provide oversight and contributions to the trial, including design and standardisation of trial-specific SSMP. PPI involvement has also extended to the qualitative research where the topic guide was developed in conjunction with respiratory participant groups.

Trial Design

The Predict & Prevent AECOPD trial is a phase III, 2 arm, multi-centre, open label, parallel-group individually randomised clinical trial investigating the use of a personalised early warning decision support system (COPDPredict™) to predict and prevent acute exacerbations of COPD. We aim to recruit 384 participants from NHS hospitals in the UK and randomise each individual in a 1:1 ratio to either SSMP with RM (control arm) or COPDPredict™ with RM (intervention arm) (*Figure 1*). The start date for the trial was 30th January 2019 and the end date for the trial is 30th March 2023.

The main outcome measure is to provide further validation, as compared to usual care, for the clinical effectiveness of COPDPredict™ to help guide and support COPD patients and their respective clinical teams in identifying exacerbations early, with an aim to reduce the total number of AECOPD-

For peer review only

induced hospital admissions in the 12 months following each patient's randomisation. The trial includes a health economic analysis examining health-related quality of life (HRQoL) using the validated questionnaire EQ-5D-5L and healthcare utilisation (as determined by a questionnaire that inquires as to hospitalisations, GP attendances and medication usage). A qualitative sub-study will assess both the end user experience and the healthcare provider experience, using Normalization Process Theory to understand the implementation and integration of the new technology[23].

Eligibility Criteria

Inclusion Criteria

- Clinically diagnosed COPD, confirmed by post-bronchodilator spirometry and defined as Post bronchodilator Forced expiratory volume/Forced vital capacity (FEV_1/FVC) <0.7 and <lower limit of normal, in the stable state
- ≥1 AECOPD in any 12-month period within the last 2 years or ≥1 hospital admission for AECOPD in the previous two years.
- Exacerbation free for at least 6 weeks
- An age of at least 18 years
- Willing and able to comply with data collection to 12 months from randomisation
- Ability to consent
- Ability to use intervention as judged by the investigator at screening, upon demonstration of the system to the patient

Exclusion Criteria

- Life expectancy <12 months -Assessment the individual investigator
- Co-enrolment into any clinical trials of investigative medicinal product (CTIMPs)
- Patients with selected co-morbidities, where these could impair use of the intervention (eg dementia)

For peer review only

Outcomes

Primary Outcome

Number of hospital admissions at 12 months post randomisation where the primary reason for admission is AECOPD. This will be obtained from participant testimony and from centrally held (HES) records. Data from the 2 sources will be cross-referenced to remove double-counting. The total number of admissions will be the sum of unique admissions from the 2 sources of data

Secondary Outcomes

Clinical Outcomes

Over a 12-month period, following randomisation;

Total in participant days, Number of visits to A&E, Number of participant defined exacerbations, Appropriate action on symptoms of AECOPD, as shown by the app, or participant testimony, Health-related quality of life (CAT) and, FEV_1 at 12 months post randomisation (from spirometry obtained during study visit)

Economic Outcomes

Health-related quality of life at baseline and 3, 6, 9 and 12-months post randomisation (EQ-5D-5L)

Healthcare utilisation at 3, 6, 9 and 12-months post randomisation, as determined by a questionnaire that inquires on hospitalisations, GP attendances and medication usage

Qualitative sub-study

End user experience and provider experience

Control Arm

A standard self-management plan (SSMP) alongside use of rescue medication (RM) containing 5 days of antibiotic and steroid treatment.

For peer review only

Intervention

COPDPredict™, consists of a patient-facing App (iOS/Android) and clinician-facing dashboard[24]. The Early Warning Decision Support System uses longitudinal remote monitoring of relevant subjective and objective data (symptoms, spirometry, biomarkers) in real-time via the App which is connected to CE-marked Bluetooth-enabled sensor peripherals. These data are used to construct COPD-relevant individual profiles such that artificial intelligence artificial intelligence (AI)-driven algorithms can then identify changes in health status to provide timely individualised alerts to patients and clinicians and sign-posting to action plans for patients.

COPDPredict™ also provides information around COPD self-management, pulmonary rehabilitation, inhaler technique and utilises gamification to help with adherence. Patients have full access to their results and can also directly message their clinical team. COPDPredict™ may signpost a participant to a self-management action plan for exacerbation management but clinical team supervision and oversight will remain throughout, as per usual care.

The clinician facing dashboard allows for "real-time" case management with the ability to remotely monitor patients and facilitate interaction. Clinicians can choose to escalate treatments based on the results being transmitted by the patients. A crucial dimension to this advanced system is that blood biomarker measurements which inform an algorithm are incorporated to enhance accuracy. Blood-based point of care (POC) analysers are used with COPDPredict™ and these POC analysers have previously been validated against laboratory CRP measures.

Recruitment and Consent

All patients must provide fully informed consent. A Participant Information Sheet (PIS) which explains the different ways in which the participant may enter the trial is provided to participants to facilitate this process. Investigators or delegate(s) adequately explain the aim, trial intervention, anticipated benefits and potential hazards of taking part in the trial to the participant. They also stress that participation is voluntary and that the participant is free to refuse to take part and may

For peer review only

withdraw from the trial at any time. Any trial specific procedures are completed after the participant has given informed consent. At each visit the participant's willingness to continue in the trial is ascertained and documented in the medical notes. Where new information becomes available which may affect the participants' decision to continue, participants will be given time to consider and if happy to continue will be re-consented. Re-consent will be documented in the medical notes.

Randomisation

After informed consent has been obtained and full participant eligibility confirmed (including exacerbation free for 6 weeks), the participant can be randomised into the trial (Table 1). Randomisation CRFs are provided to investigators and may be used to collate the necessary information prior to randomisation. Participants are randomised by computer/telephone at the level of the individual in a 1:1 ratio to either SSMP with RM (control arm) or COPDPredict™ with RM (intervention arm).

A minimisation algorithm will be used within the online randomisation system to ensure balance in the treatment allocation over centre, age (<60, ≥60 years) and severity of disease as per Global Initiative for Obstructive Lung Disease (GOLD)[1].

The participants are randomised to one of the following:

Control Arm

A standard self-management plan (SSMP) alongside use of rescue medication (RM) containing 5 days of antibiotic and steroid treatment.

Intervention arm

Supported self-management using the COPDPredict™ App, involving personalised alerts to both patients and the clinical care team.

A 'random element' will be included in the minimisation algorithm, so that each participant has a probability, of being randomised to the opposite treatment that they would have otherwise

For peer review only

received. Once randomised patients will also be asked to complete baseline quality of life booklet which contains both the EQ-5D-5L and CAT questionnaire.

For peer review only

Assessment schedule

Visit	Hospital Discharge/ Initial appointment	Personal Baseline Day 1 after 6 weeks exacerbation free	Personal Baseline Day 7 (±2 days)	End of Personal Baseline Period	Approximately 4 times during any exacerbation (home visit or clinic)	Weekly until Week 52	Week 13 Telephone follow Up +/-1 week	Week 26 Hospital/ Telephone follow Up +/-1 week	Week 39 Telephone follow Up +/-1 week	Week 52 Telephone /Hospital follow Up +/- 2 weeks	Patients daily wellbeing completion post two-week bassline period
Eligibility check	X										
Valid informed consent	X										
Randomisation		X									
Height and Weight	X									X	
Concomitant medication	X	X		X				X	X	X	
Baseline medical history taken$^{\alpha}$	X										
Request to GP for rescue medication	X						(X)	(X)	(X)		
Assessment of (S)AEs	X				(X)		X	X	X	X	

For peer review only

Questionnaires (QoL and health utilisation)	X						X	X	X	X	
Spirometry	X							X		X	
Intervention only below this line					Intervention only below this line						
Provision of equipment & participant training		X									
Spirometry	X	X	X		(X)	X					
Symptom control markers	X	X	X	X	(X)	X	X	X	X	X	
Point of care blood test		X		X	(X)						
Clinical histories		X		X			X	X	X	X	
Wellbeing (App)											X

(X) denotes only when required. If rescue medication has not been used, fresh prescription requests will not be necessary

During the baseline period and any subsequent exacerbations patients will be entering well-being data on the App which will be related to lifestyle choices and the end-user experience. This data will not form part of the formal analysis of quality of life.

Table 1: Schedule of assessments

For peer review only

Statistical Analysis

Sample size

The justification of the sample size is based on previous evidence[25] that had shown a mean estimate of 2.5 COPD admissions in the previous year in the control group. To detect a difference of 1 admission in the mean number of admissions between groups using the standard methods of difference between means and assuming standard deviation of 2.6 with 90% power and a type I error rate of 5% (two-sided), 144 participants per group will need to be randomised, 288 in total. Assuming and adjusting for a 25% loss to follow-up/ drop-out rate, 384 participants will need to be recruited.

Analysis of Outcome Measures

The primary comparison groups will be composed of those randomised to the use of COPDPredict™ system and those randomised to the standard self-management plan (SSMP). All analyses will be based on the intention to treat principle[26], with all patients analysed in the treatment groups to which they were allocated irrespective of compliance with the randomised allocated treatment, and all patients will be included in the analyses. For all outcomes, summary statistics (e.g. mean differences, relative risks) will be reported and 95% confidence intervals will be constructed where appropriate. A two-sided p-value of <0.05 will be considered statistically significant.

Primary Outcome Measure

The primary analysis for this study will be to compare rates of hospital admission between the treatment groups (COPDPredict™ versus Usual care) over 12 months, following randomisation, where the primary reason for admission is an AECOPD event. These hospital admissions per person will be analysed using Poisson regression models adjusting for treatment group and minimisation variables. If there is over dispersion a negative binomial regression model adjusting for treatment group and the same minimisation variables will be taken into consideration. Point estimates

For peer review only

(incidence rates) will be provided and accompanied with 95% confidence intervals and respective p-values.

Secondary Outcome Measures

Recorded observations taken over a 12-month period, following randomisation:

Total in-hospital days and FEV_1 – These variables will be summarised using basic descriptive statistics (Mean, SD)). We may also consider a mixed linear regression model, to estimate differences between the intervention group supported with 95% CI adjusting for baseline variables and minimisation variables (centre being a random effects variable).

Health-related quality of life questionnaires - EQ5D-5L and CAT will be converted into scores and analysed using a mixed linear regression model, adjusting for the intervention group, baseline and minimisation variables as before.

Number of A&E visits, participant defined exacerbations and healthcare utilisation - Where the participant experienced an episode, the data during the 12 months from randomisation will be analysed using mixed effects log-binomial regression techniques with presentation of relative risk and 95% confidence intervals. Furthermore, sensitivity analysis based on patients' experience of multiple visits to A&E and participant defined exacerbation will be calculated using Poisson regression techniques and relative risk supported with 95% CI.

Symptom control marker association to clinical decision - The diagnostic accuracy of COPDPredict™ and Usual care will be evaluated by calculating sensitivity, specificity, positive and negative predictive values, and area under curve (AUC)/receiver operating characteristics (ROC) together with 95% CI.

Subgroup analyses

Subgroup analyses will be limited to the same variables used in the minimisation algorithm, apart from centre. Tests for statistical heterogeneity (e.g. by including the treatment group by subgroup

For peer review only - http://bmjopen.bmj.com/site/about/guidelines.xhtml

interaction parameter in the statistical model) will be performed prior to any examination of effect estimate within subgroups. The results of subgroup analyses will be treated with caution and will be used for the purposes of hypothesis generation only.

Missing data and sensitivity analyses

Participants with missing primary outcome data will not be included in the primary analysis in the first instance. This presents a risk of bias, and sensitivity analyses will be undertaken to assess the possible impact. This will consist of simulating the missing response using a multiple imputation approach[27]. Parameters used to simulate the missing response will include the minimisation variables, intervention group and previous response. Full details are included in the statistical analysis plan (SAP).

Adverse events and serious adverse events

Patients with COPD can have high disease burden, such that the trial population will be older, with co-morbidities such as osteoporosis[28], cardiovascular disease or raised cardiovascular risk[29], along with associated complications and symptoms including abnormal lab results. A relatively high number of adverse events are anticipated as a result of the patients' existing past medical history[30]. However, this study is examining the role of increased participant surveillance and therefore few, if any, foreseeable risks of direct harm associated with the study intervention are anticipated. AE reporting will therefore be limited to those events which are required for trial monitoring or outcome assessment.

All events which meet the definition of serious must be recorded in the participant notes, but for trial purposes these following events do not require reporting on the SAE Form. Such events are "safety reporting exempt".

• Planned hospitalisation for a pre-existing condition, or a procedure required by the trial protocol without serious deterioration in health, is not considered a serious adverse event.

For peer review only - http://bmjopen.bmj.com/site/about/guidelines.xhtml

• An overnight stay in hospital that is due to transportation, organisational or accommodation problems, and without medical background SAES requiring expedited reporting

Any other events that fulfil the usual definition of an SAE will be reported immediately.

Any death occurring during the Trial Protocol defined follow up period (12 months) must be reported as an SAE within 24 hours of the local investigator becoming aware of the event.

Health economics

Within -trial analysis

The economic evaluation alongside the trial will take the form of an incremental cost-effectiveness analysis to estimate cost per hospital admission avoided and incremental cost-utility analysis to estimate cost per quality adjusted life year (QALY) gained. Base-case analyses will be from a healthcare perspective over 12 months follow-up using individual participant level data on costs and outcomes from the clinical trial. Additional analyses will be undertaken from a broader societal perspective. Resource use data will be collected by questionnaire, capturing information on hospitalisations for exacerbations, COPD-related primary and secondary care attendances and medication usage. Data will also be collected during the trial on resource use implications of the intervention. Information on participant incurred costs, time off work and impact on activities is collected within the economic questionnaire. QALYs will be calculated from EQ-5D-5L responses. Sensitivity analysis will be undertaken and cost-effectiveness planes and cost-effectiveness acceptability curves presented.

Model-based analysis

A decision model will be constructed to extrapolate beyond trial results over five years, with costs and benefits discounted at 3.5%. The model will be the first decision analysis to use GOLD stages ABCD[1] and will consider the ability of the COPDPredict™ supported self-management to impact movements between GOLD ABCD stages, but also the number of exacerbations within those stages.

For peer review only - http://bmjopen.bmj.com/site/about/guidelines.xhtml

Costs associated with delivering COPDPredict™, exacerbations, and routine care will be obtained from the trial. Quality of life scores on each intervention will also be obtained from trial data using EQ-5D-5L. Longer-term costs, quality of life scores, and transitions between stages, and number of exacerbations will be obtained from analysis of ABCD cohorts from within the BLISS[2] study, a prospective cohort study with 3-year follow-up period. The model will estimate cost per QALY gained of COPDPredict™ supported self-management versus standard self-management from a healthcare perspective. Probabilistic sensitivity analysis will be undertaken to simultaneously incorporate all parameter uncertainty. Cost-effectiveness planes and cost-effectiveness acceptability curves will be presented to show the probability the intervention is cost-effective at different cost/QALY thresholds. A value of information analysis will assess the value of further trials to reduce parameter uncertainty and identify parameters which would be most valuable in reducing that uncertainty[31].

Qualitative sub-study

In order to frame this study, we draw on Normalisation Process Theory[32], which proposes four constructs essential to effect implementation of new technologies: *coherence* (sense making work to understand the possibilities of a new technology), *cognitive participation* (relational work as new technology begins to be used), *collective action* (operational work enacted to make a technology function in context) and *reflexive monitoring* (the appraisal of work that people do to assess how the new technology affects them and others). Participants will include health care professionals, patients and wider policy stakeholders and will be purposively sampled to maximise diversity. A topic guide developed by drawing on existing literature and theory will focus on attitudes to and practices around self-management of COPD and the use and implementation of digital technologies. Interviews will be audio recorded and transcribed verbatim, before subjecting the data to a two-stage approach to analysis[33]: first, a descriptive framework analysis[34] followed by mapping onto the NPT constructs. We anticipate conducting approximately30 interviews but this will be informed by the data and analysis35].

For peer review only

Ethics and dissemination:

This study protocol is reported in accordance with the guidance set out in the SPIRIT statement. Predict and Prevent AECOPD has obtained ethical approval in England (19/LO/1939). Upon completion of the trial and publication of results a lay findings summary will be disseminated to trial participants.

Trial Oversight

Trial oversight will be provided by the TMG, an independent DMC and independent TSC. Interim analyses of major outcome measures and safety data will be conducted and provided in strict confidence to the DMC.

Potential Impact

Predict & Prevent AECOPD is a pragmatic, national, multicentre, RCT which aims to provide robust evidence around the clinical and cost effectiveness of COPDPredict™ by analysing the number of patients admitted to hospital in a 12month period post randomisation. It is hoped the outcome of this trial could be used to provide robust evidence to the management of patients with COPD and their exacerbations in the future.

Contributorship statement

All authors have made substantial contributions to the conception or design of the work, or the acquisition, analysis or interpretation of data. DK, AT, HJ, RM, SJ, NG, NP and MS drafted the manuscript and revised it critically for important intellectual content. All authors approved final version and agreed to be accountable for all aspects of the work in ensuring that questions related to the accuracy or integrity of any part of the work are appropriately investigated and resolved.

Competing Interest Statement

The CI of the trial (AT) does not have any relevant direct financial disclosures, nor do members of the trial management group (TMG) who are authors of this paper, with the exception of NP who is a

For peer review only

founder, director and share-holder of NEPeSMO, who developed the intervention. In addition, MS is CI of the overall NIHR-funded project and is a founder, director and share-holder of NEPeSMO. NEPeSMO is a start-up company from the University Hospitals of North Midlands NHS Trust, owns all intellectual property rights of COPDPredict™ and is a project collaborator on the grant.

AT has grants from pharmaceutical companies working in the area of COPD (Chiesi, AstraZeneca) and has conducted advisory work for such (Boehringer, CSL Behring) but not in the area of medical devices or admission prevention. Neither has she worked for, or received monies from, any company working on admission prevention in the last 3 years.

Funding Statement & Disclaimer

This report is independent research funded by the National Institute for Health Research (Invention for Innovation, A Personalised Early Warning Decision Support System with novel Saliva Bio-Profiling to Predict and Prevent Acute Exacerbations of Chronic Obstructive Pulmonary Disease – 'Predict&prevent AECOPD, NIHR200002).

For peer review only

References

1. Global Initiative for Obstructive Lung Disease, [Accessed 13 June 2018]. [Available from: www.goldcopd.com].

2. Estimating the economic burden of respiratory illness in the UK. London: British Lung Foundation 2016.

3. The battle for breath - the impact of lung disease in the UK. London: British Lung Foundation, 2016.

4. Roberts CM, Stone RA, Lowe D, Pursey NA, Buckingham RJ. Co-morbidities and 90-day outcomes in hospitalized COPD exacerbations. Copd. 2011;8(5):354-61.

5. Stone RA HB, Lowe D, Searle L, Skipper E, Welham S, et al. . National COPD audit programme. COPD: Who cares matters. London, UK: Royal College of Physicians, 2015. Contract No.: eISBN 978-1-86016-559-7.

6. Hurst JR, Vestbo J, Anzueto A, Locantore N, Mullerova H, Tal-Singer R, et al. Susceptibility to exacerbation in chronic obstructive pulmonary disease. N Engl J Med. 2010;363(12):1128-38.

7. Merinopoulou E, Raluy-Callado M, Ramagopalan S, MacLachlan S, Khalid JM. COPD exacerbations by disease severity in England. Int J Chron Obstruct Pulmon Dis. 2016;11:697-709.

8. Stone RA HB, McMillan V, Saleem Khan M, Searle L, Skipper E, et al. COPD: Who cares when it matters most? London: Royal College of Physicians, 2017.

9. Steer J, Gibson GJ, Bourke SC. Longitudinal change in quality of life following hospitalisation for acute exacerbations of COPD. BMJ Open Respir Res. 2015;2(1):e000069.

10. Jo YS, Kim SK, Park SJ, Um S-J, Park Y-B, Jung KS, et al. Longitudinal change of FEV(1) and inspiratory capacity: clinical implication and relevance to exacerbation risk in patients with COPD. Int J Chron Obstruct Pulmon Dis. 2019;14:361-9.

For peer review only

11. Qureshi H SA, Hanania NA. Chronic obstructive pulmonary disease exacerbations: latest evidence and clinical implications. Therapeutic advances in chronic disease. 2014;5(5):212-27. Epub 2014/09/02.

12. Oba, Y., Keeney, E., Ghatehorde, N. and Dias, S., 2018. Dual combination therapy versus long-acting bronchodilators alone for chronic obstructive pulmonary disease (COPD): a systematic review and network meta-analysis. Cochrane Database of Systematic Reviews, [online] 2018(12). Available at: <https://www.cochranelibrary.com/cdsr/doi/10.1002/14651858.CD012620.pub2/full> [Accessed 5 October 2021].

13. Yang, I., Clarke, M., Sim, E. and Fong, K., 2012. Inhaled corticosteroids for stable chronic obstructive pulmonary disease. Cochrane Database of Systematic Reviews, [online] Available at: <http://Inhaled corticosteroids for stable chronic obstructive pulmonary disease> [Accessed 1 September 2021].

14. Ryrso CK, Godtfredsen NS, Kofod LM, Lavesen M, Mogensen L, Tobberup R, et al. Lower mortality after early supervised pulmonary rehabilitation following COPD-exacerbations: a systematic review and meta-analysis. BMC Pulm Med. 2018;18(1):154.

15. Ni, W., Shao, X., Cai, X., Wei, C., Cui, J., Wang, R. and Liu, Y., 2015. Prophylactic Use of Macrolide Antibiotics for the Prevention of Chronic Obstructive Pulmonary Disease Exacerbation: A Meta-Analysis. PLOS ONE, 10(3), p.e0121257.

16. Lawless, M., Burgess, M. and Bourke, S., 2022. Impact of COVID-19 on Hospital Admissions for COPD Exacerbation: Lessons for Future Care. Medicina, 58(1), p.66.

17. Nice.org.uk. 2018. Overview | Chronic obstructive pulmonary disease in over 16s: diagnosis and management | Guidance | NICE. [online] Available at: <https://www.nice.org.uk/guidance/ng115> [Accessed 23 July 2022].

18. Global Initiative for Chronic Obstructive Lung Disease - GOLD. 2022. 2022 GOLD Reports - Global Initiative for Chronic Obstructive Lung Disease - GOLD. [online] Available at: <https://goldcopd.org/2022-gold-reports-2/> [Accessed 23 July 2022].

19. Jolly K, Majothi S, Sitch AJ, Heneghan NR, Riley RD, Moore DJ, et al. Self-management of health care behaviors for COPD: a systematic review and meta-analysis. Int J Chron Obstruct Pulmon Dis. 2016;11:305-26.

20. Vijayasaratha K, Stockley RA. Relationship between frequency, length, and treatment outcome of exacerbations to baseline lung function and lung density in alpha-1 antitrypsin-deficient COPD. Int J Chron Obstruct Pulmon Dis. 2012;7:789-96.

21. Celli BR, Locantore N, Yates J, Tal-Singer R, Miller BE, Bakke P, et al. Inflammatory biomarkers improve clinical prediction of mortality in chronic obstructive pulmonary disease. Am J Respir Crit Care Med. 2012;185(10):1065-72.

For peer review only

22. Patel N, Kinmond K, Jones P, Birks P, Spiteri MA. Validation of COPDPredict™: Unique Combination of Remote Monitoring and Exacerbation Prediction to Support Preventative Management of COPD Exacerbations. International Journal of Chronic Obstructive Pulmonary Disease 2021, 16:1887-1899

23. May CR, Mair F, Finch T, MacFarlane A, Dowrick C, Treweek S, Rapley T, Ballini L, Ong BN, Rogers A, Murray E. Development of a theory of implementation and integration: Normalization Process Theory. Implementation Science. 2009 Dec;4(1):1-9.

24. Nepesmo.com. 2017. NEPeSMO – Predict to Prevent. [online] Available at: <https://nepesmo.com/#:~:text=NEPeSMO%20offers%20a%20smart%20CE,to%20patients%20living%20with%20COPD> [Accessed 16 June 2021].

25. Pinnock H, Hanley J, McCloughan L, Todd A, Krishan A, Lewis S, et al. Effectiveness of telemonitoring integrated into existing clinical services on hospital admission for exacerbation of chronic obstructive pulmonary disease: researcher blind, multicentre, randomised controlled trial. Bmj. 2013;347:f6070

26. Sandeep K Gupta. Intention-to-treat concept: A Review. Perspect Clin Res 2011:2(3);109-112

27 . White IR, Royston P, Wood AM. Multiple imputation using chained equations. Stats in Medicine 2011, (30) 377-399.

28. Vrieze A, de Greef MH, Wijkstra PJ, Wempe JB. Low bone mineral density in COPD patients related to worse lung function, low weight and decreased fat-free mass. Osteoporos Int. 2007;18(9):1197-202.

29. Patel AR, Kowlessar BS, Donaldson GC, Mackay AJ, Singh R, George SN, et al. Cardiovascular risk, myocardial injury, and exacerbations of chronic obstructive pulmonary disease. Am J Respir Crit Care Med. 2013;188(9):1091-9.

30. Miller J, Edwards LD, Agusti A, Bakke P, Calverley PM, Celli B, et al. Comorbidity, systemic inflammation and outcomes in the ECLIPSE cohort. Respir Med. 2013;107(9):1376-84.

31. Global Initiative for Obstructive Lung Disease COPD Diagnosis and Management At-A-Glance Desk Reference 2017 [online] https://goldcopd.org/archived-reports/ Accessed 03/09/21

32. May C, Finch T. Implementing, Embedding, and Integrating Practices: An Outline of Normalization Process Theory. Sociology. 2009;43(3):535-54.

33. Ritchie J, Spencer L. Qualitative data analysis for applied policy research. In: Bryman A, Burgess RG, editors. London: Routledge; 1994.

34. Macfarlane A, O'Reilly-de Brún M. Using a theory-driven conceptual framework in qualitative health research. Qual Health Res. 2012;22(5):607-18.

For peer review only

35. O'Cathain A, Hoddinott P, Lewin S, Thomas KJ, Young B, Adamson J, et al. Maximising the impact of qualitative research in feasibility studies for randomised controlled trials: guidance for researchers. Pilot and Feasibility Studies. 2015;1(1):32.

Figure Legends

Figure 1: Flow diagram of the Predict & Prevent trial

Table Legends

Table 1: Schedule of assessments

For peer review only

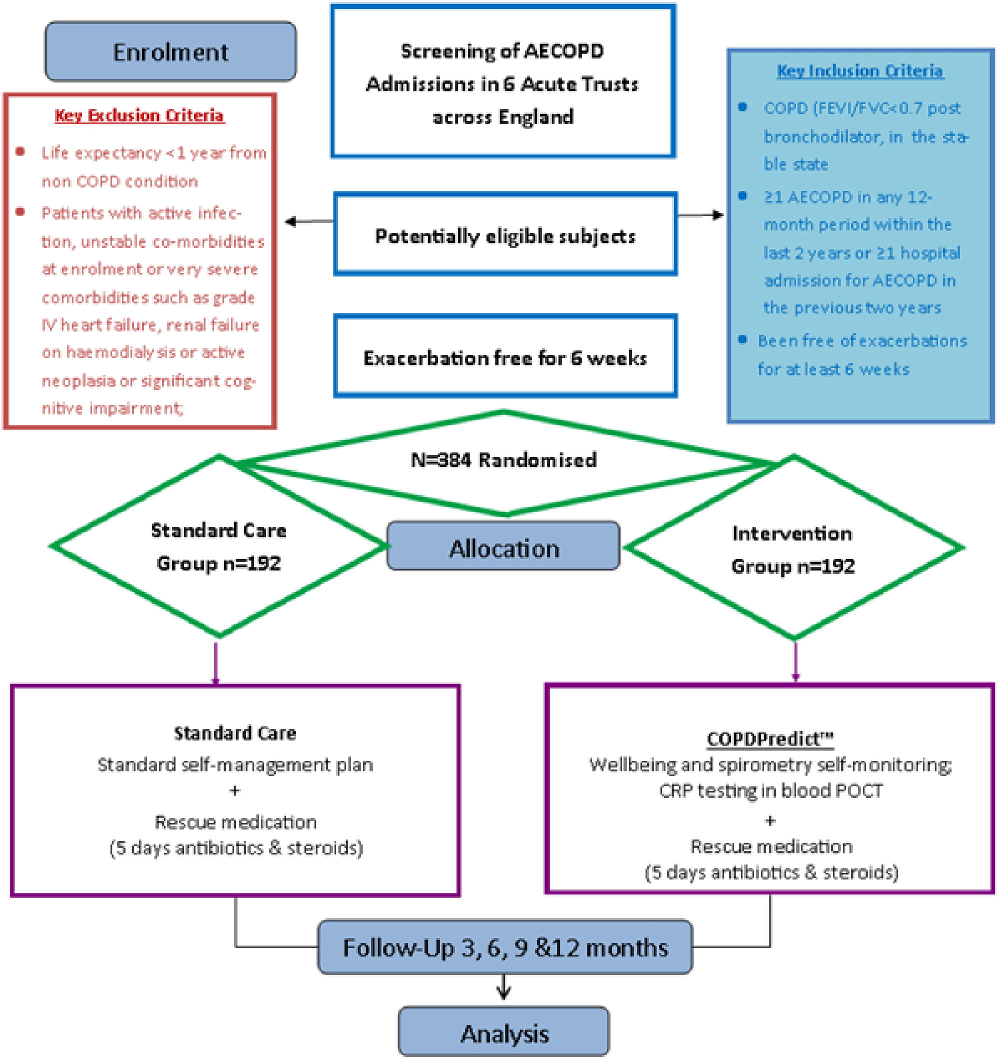

Figure 1: Flow diagram of the Predict & Prevent trial

266x297mm (57 x 57 DPI)

SPIRIT
STANDARD PROTOCOL ITEMS: RECOMMENDATIONS FOR INTERVENTIONAL TRIALS

SPIRIT 2013 Checklist: Recommended items to address in a clinical trial protocol and related documents*

Section/item	ItemNo	Description	Section of manuscript
**Administrative information**			
Title	1	Descriptive title identifying the study design, population, interventions, and, if applicable, trial acronym	Page 5 methods
Trial registration	2a	Trial identifier and registry name. If not yet registered, name of intended registry	Page 2 abstract
	2b	All items from the World Health Organization Trial Registration Data Set	
Protocol version	3	Date and version identifier	V9.0 vd03-Mar-22
Funding	4	Sources and types of financial, material, and other support	Page 2 NIHR Statement
Roles and responsibilities	5a	Names, affiliations, and roles of protocol contributors	Page 1
	5b	Name and contact information for the trial sponsor	Page 1 and 2
	5c	Role of study sponsor and funders, if any, in study design; collection, management, analysis, and interpretation of data; writing of the report; and the decision to submit the report for publication, including whether they will have ultimate authority over any of these activities	

For peer review only

	5d	Composition, roles, and responsibilities of the coordinating centre, steering committee, endpoint adjudication committee, data management team, and other individuals or groups overseeing the trial, if applicable (see Item 21a for data monitoring committee)	
**Introduction**			
Background and rationale	6a	Description of research question and justification for undertaking the trial, including summary of relevant studies (published and unpublished) examining benefits and harms for each intervention	Page 4-5
	6b	Explanation for choice of comparators	
Objectives	7	Specific objectives or hypotheses	Page 5
Trial design	8	Description of trial design including type of trial (eg, parallel group, crossover, factorial, single group), allocation ratio, and framework (eg, superiority, equivalence, noninferiority, exploratory)	Page 5-6
**Methods: Participants, interventions, and outcomes**			
Study setting	9	Description of study settings (eg, community clinic, academic hospital) and list of countries where data will be collected. Reference to where list of study sites can be obtained	Page 5

For peer review only

Eligibility criteria	10	Inclusion and exclusion criteria for participants. If applicable, eligibility criteria for study centres and individuals who will perform the interventions (eg, surgeons, psychotherapists)	Page 6
Interventions	11a	Interventions for each group with sufficient detail to allow replication, including how and when they will be administered	Page 7
	11b	Criteria for discontinuing or modifying allocated interventions for a given trial participant (eg, drug dose change in response to harms, participant request, or improving/worsening disease)	N/A
	11c	Strategies to improve adherence to intervention protocols, and any procedures for monitoring adherence (eg, drug tablet return, laboratory tests)	N/A
	11d	Relevant concomitant care and interventions that are permitted or prohibited during the trial	N/A
Outcomes	12	Primary, secondary, and other outcomes, including the specific measurement variable (eg, systolic blood pressure), analysis metric (eg, change from baseline, final value, time to event), method of aggregation (eg, median, proportion), and time point for each outcome. Explanation of the clinical relevance of chosen efficacy and harm outcomes is strongly recommended	Page 6/7

For peer review only - http://bmjopen.bmj.com/site/about/guidelines.xhtml

Participant timeline	13	Time schedule of enrolment, interventions (including any run-ins and washouts), assessments, and visits for participants. A schematic diagram is highly recommended (see Figure)	Page 10
Sample size	14	Estimated number of participants needed to achieve study objectives and how it was determined, including clinical and statistical assumptions supporting any sample size calculations	Page 5
Recruitment	15	Strategies for achieving adequate participant enrolment to reach target sample size	Page 8
**Methods: Assignment of interventions (for controlled trials)**			
Allocation:			
Sequence generation	16a	Method of generating the allocation sequence (eg, computer-generated random numbers), and list of any factors for stratification. To reduce predictability of a random sequence, details of any planned restriction (eg, blocking) should be provided in a separate document that is unavailable to those who enrol participants or assign interventions	Page 8 and 9
Allocation concealment mechanism	16b	Mechanism of implementing the allocation sequence (eg, central telephone; sequentially numbered, opaque, sealed envelopes), describing any steps to conceal the sequence until interventions are assigned	Protocol

Implementation	16c	Who will generate the allocation sequence, who will enrol participants, and who will assign participants to interventions	Protocol
Blinding (masking)	17a	Who will be blinded after assignment to interventions (eg, trial participants, care providers, outcome assessors, data analysts), and how	N/A
	17b	If blinded, circumstances under which unblinding is permissible, and procedure for revealing a participant's allocated intervention during the trial	

Methods: Data collection, management, and analysis

Data collection methods	18a	Plans for assessment and collection of outcome, baseline, and other trial data, including any related processes to promote data quality (eg, duplicate measurements, training of assessors) and a description of study instruments (eg, questionnaires, laboratory tests) along with their reliability and validity, if known. Reference to where data collection forms can be found, if not in the protocol	Page 12-16 and Protocol
	18b	Plans to promote participant retention and complete follow-up, including list of any outcome data to be collected for participants who discontinue or deviate from intervention protocols	Page 12-13

Data management	19	Plans for data entry, coding, security, and storage, including any related processes to promote data quality (eg, double data entry; range checks for data values). Reference to where details of data management procedures can be found, if not in the protocol	Protocol
Statistical methods	20a	Statistical methods for analysing primary and secondary outcomes. Reference to where other details of the statistical analysis plan can be found, if not in the protocol	Protocol
	20b	Methods for any additional analyses (eg, subgroup and adjusted analyses)	Page 15-16
	20c	Definition of analysis population relating to protocol non-adherence (eg, as randomised analysis), and any statistical methods to handle missing data (eg, multiple imputation)	Page 14
**Methods: Monitoring**			
Data monitoring	21a	Composition of data monitoring committee (DMC); summary of its role and reporting structure; statement of whether it is independent from the sponsor and competing interests; and reference to where further details about its charter can be found, if not in the protocol. Alternatively, an explanation of why a DMC is not needed	Protocol

	21b	Description of any interim analyses and stopping guidelines, including who will have access to these interim results and make the final decision to terminate the trial	Protocol
Harms	22	Plans for collecting, assessing, reporting, and managing solicited and spontaneously reported adverse events and other unintended effects of trial interventions or trial conduct	Protocol
Auditing	23	Frequency and procedures for auditing trial conduct, if any, and whether the process will be independent from investigators and the sponsor	
**Ethics and dissemination**			
Research ethics approval	24	Plans for seeking research ethics committee/institutional review board (REC/IRB) approval	Page 16
Protocol amendments	25	Plans for communicating important protocol modifications (eg, changes to eligibility criteria, outcomes, analyses) to relevant parties (eg, investigators, REC/IRBs, trial participants, trial registries, journals, regulators)	Protocol
Consent or assent	26a	Who will obtain informed consent or assent from potential trial participants or authorised surrogates, and how (see Item 32)	Protocol
	26b	Additional consent provisions for collection and use of participant data and biological specimens in ancillary studies, if applicable	

For peer review only

Confidentiality	27	How personal information about potential and enrolled participants will be collected, shared, and maintained in order to protect confidentiality before, during, and after the trial	Protocol
Declaration of interests	28	Financial and other competing interests for principal investigators for the overall trial and each study site	Page 17
Access to data	29	Statement of who will have access to the final trial dataset, and disclosure of contractual agreements that limit such access for investigators	Protocol
Ancillary and post-trial care	30	Provisions, if any, for ancillary and post-trial care, and for compensation to those who suffer harm from trial participation	N/A
Dissemination policy	31a	Plans for investigators and sponsor to communicate trial results to participants, healthcare professionals, the public, and other relevant groups (eg, via publication, reporting in results databases, or other data sharing arrangements), including any publication restrictions	Page 16
	31b	Authorship eligibility guidelines and any intended use of professional writers	
	31c	Plans, if any, for granting public access to the full protocol, participant-level dataset, and statistical code	N/A

Appendices

For peer review only - http://bmjopen.bmj.com/site/about/guidelines.xhtml

Informed consent materials	32	Model consent form and other related documentation given to participants and authorised surrogates	ICF
Biological specimens	33	Plans for collection, laboratory evaluation, and storage of biological specimens for genetic or molecular analysis in the current trial and for future use in ancillary studies, if applicable	N/A

*It is strongly recommended that this checklist be read in conjunction with the SPIRIT 2013 Explanation & Elaboration for important clarification on the items. Amendments to the protocol should be tracked and dated. The SPIRIT checklist is copyrighted by the SPIRIT Group under the Creative Commons "Attribution-NonCommercial-NoDerivs 3.0 Unported" license.

For peer review only

BMJ Open

A phase III, 2 arm, multi-centre, open label, parallel-group randomised designed clinical investigation of the use of a personalised early warning decision support system to predict and prevent acute exacerbations of Chronic Obstructive Pulmonary Disease - 'Predict & Prevent AECOPD' -Study Protocol

Journal:	*BMJ Open*
Manuscript ID	bmjopen-2022-061050.R5
Article Type:	Protocol
Date Submitted by the Author:	16-Jan-2023

Complete List of Authors:	Kaur, Dalbir; University of Warwick<br>Mehta, Rajnikant ; University of birmingham<br>Jarrett, Hugh; University of Birmingham<br>Jowett, Sue; University of Birmingham, Health Economics Unit<br>Gale, Nicola K.; University of Birmingham<br>Turner, Alice; University of Birmingham, Institute of Inflammation & Aging; Heart of England NHS Foundation Trust<br>Spiteri, Monica; University Hospitals of North Midlands NHS Trust<br>Patel, Neil; NEPESMO

**Primary Subject Heading**:	Respiratory medicine
Secondary Subject Heading:	Smoking and tobacco
Keywords:	RESPIRATORY MEDICINE (see Thoracic Medicine), Chronic airways disease < THORACIC MEDICINE, Thoracic medicine < INTERNAL MEDICINE

SCHOLARONE™
Manuscripts

A phase III, 2 arm, multi-centre, open label, parallel-group randomised designed clinical investigation of the use of a personalised early warning decision support system to predict and prevent acute exacerbations of Chronic Obstructive Pulmonary Disease - 'Predict & Prevent AECOPD' -Study Protocol

References: 35
Abstract count: 275
Word count: 3889

D Kaur, R Mehta, H Jarrett, S Jowett, N Gale, AM Turner, MA Spiteri, N Patel

Dalbir Kaur[1] Corresponding author
Email: dalbir.kaur@warwick.ac.uk

Rajnikant Mehta[2]
Email: R.L.Mehta@bham.ac.uk

Hugh Jarrett[3]
Email: h.jarrett@bham.ac.uk

Sue Jowett[4]
Email: S.JOWETT@bham.ac.uk

Nicola Gale[5]
Email: N.Gale@bham.ac.uk

Alice M Turner[6]
Email: A.M.Turner@bham.ac.uk

Monica A Spiteri[7]
Email: Monica.Spiteri@uhnm.nhs.uk

Neil Patel[8]
Email: neil.patel@nepesmo.com

[1]Senior Project Manager, Warwick Clinical Trials Unit (BWCTU), Warwick Medical School University of Warwick Coventry, CV4 7ALInstitute

[2]Senior Medical Statistician, Birmingham Clinical Trials Unit (BCTU), Institute of Applied Health Research, College of Medical and Dental Sciences, University of Birmingham, Edgbaston, Birmingham, B15 2TT

[3]Trials Management Team Leader, Birmingham Clinical Trials Unit (BCTU), Institute of Applied Health Research, College of Medical and Dental Sciences, University of Birmingham, Edgbaston, Birmingham, B15 2TT

[4]Professor of Health Economics, Health Economics Unit, Institute of Applied Health Research, College of Medical and Dental Sciences, University of Birmingham, Edgbaston, Birmingham, B15 2TT

For peer review only

[5]Professor of Health Policy and Sociology, Health Services Management Centre, School of Social Policy Director of Postgraduate Research, College of Social Sciences, University of Birmingham, Edgbaston, Birmingham, B15 2TT

[6] Professor of Respiratory Medicine and Honorary Consultant Respiratory Physician, Institute for Applied Health Research, University of Birmingham, Birmingham, UK, B15 2TT

[7]Director of Respiratory Research, Academic Research Unit, Courtyard Annexe - C Block, Royal Stoke University Hospital, University Hospitals of North Midlands NHS Trust, Newcastle Road, Staffordshire, UK, ST4 6QG

[8]Chief Medical Officer, NEPESMO Ltd, 76 King Street, Manchester, M2 4NH, United Kingdom
Word count: 3487 (excluding title page, abstract, table, legend and references)

Abstract

Introduction

With 65 million cases globally, chronic obstructive pulmonary disease (COPD) is the 4th leading cause of death and imposes a heavy burden on patients' lives and healthcare resources worldwide. Around half of all patients with COPD have frequent (≥2 per year) AECOPD[5]. Rapid readmissions are also common. Exacerbations impact significantly on COPD outcomes, causing significant lung function decline. Prompt exacerbation management optimises recovery and delays the time to the next acute episode.

Methods/analysis

The Predict & Prevent AECOPD trial is a phase III, 2 arm, multi-centre, open label, parallel-group individually randomised clinical trial investigating the use of a personalised early warning decision support system (COPDPredict™) to predict and prevent acute exacerbations of COPD. We aim to recruit 384 participants and randomise each individual in a 1:1 ratio to either standard self-management plans (SSMP) with Rescue Medication (RM) (control arm) or COPDPredict™ with RM (intervention arm)

The trial will inform the future standard of care regarding management of exacerbations in COPD patients. The main outcome measure is to provide further validation, as compared to usual care, for the clinical effectiveness of COPDPredict™ to help guide and support COPD patients and their respective clinical teams in identifying exacerbations early, with an aim to reduce the total number of AECOPD-induced hospital admissions in the 12 months following each patient's randomisation.

Ethics and Dissemination

This study protocol is reported in accordance with the guidance set out in the SPIRIT statement. Predict and Prevent AECOPD has obtained ethical approval in England (19/LO/1939). Upon completion of the trial and publication of results a lay findings summary will be disseminated to trial participants.

Trials Registration: Clinical trials.gov NCT04136418

Keywords: Respiratory, COPD, Exacerbations, Symptom Control Markers, COPDPredict™

For peer review only

NIHR Statement

This work is supported by the National Institute for Health Research (NIHR) under its Invention for Innovation (i4i) Programme (Grant Reference Number NIHR200002). The views expressed are those of the author(s) and not necessarily those of the NHS, the NIHR or the Department of Health.

Strengths and Limitations

- This is the first major trial of real-time observation, combined with personalised self-management in COPD
- The minimisation algorithm addresses major associations of AECOPD admission, such that these should not confound results
- There is holistic exploration of deployment of a complex tool, because we have embedded qualitative work with staff and patients
- We have addressed digital exclusion by ensuring that funding is provided for mobile data, where patients do not have this, or WiFi, already
- Logistical issues to do with provision of mobile CRP measurement means we could only include a limited number of sites, and these do not reflect the whole of the UK

For peer review only

Background

With 65 million cases globally, chronic obstructive pulmonary disease (COPD) is the 4th leading cause of death and imposes a heavy burden on patients' lives and healthcare resources worldwide[1]. UK death rates are currently double the European Union (EU) average with over 30,000 people dying yearly and annual COPD-related NHS direct costs exceeding £800m[2]. Patients can have acute exacerbations of their COPD (AECOPD), which reduce quality of life, and lead to 140,000 emergency hospital admissions a year in the UK[3]. Indeed, AECOPD remain the 2nd commonest cause of emergency hospital admissions and 1 in 3 of these patients (sometimes more) will be readmitted within 3 months[4,5]. Hospitalisation itself carries a poor prognosis with an increased mortality risk.

Around half of all patients with COPD have frequent (≥2 per year) AECOPD[6,7]. Rapid readmissions are also common – the national COPD audit has shown that 43% of patients with COPD who were admitted are back in hospital within 90 days[8], and up to 71% by 12 months[9]. Exacerbations impact significantly on COPD outcomes, causing significant lung function decline[10]. Prompt exacerbation management optimises recovery and delays the time to the next acute episode[11], with NICE COPD guidelines highlighting a time window (prodrome) between an initial exacerbation's symptoms/signs and subsequent hospitalisation. Within this prodrome there is an opportunity to intervene. Current practice for COPD patients is that they are encouraged to recognise AECOPD, via standard self-management plans (SSMPs), and treat, using rescue medication (RM), but in many cases because of day-to-day variability in symptoms, the start of an exacerbation goes unrecognised and untreated. This inability/uncertainty to recognise and treat exacerbations in their early phase can lead to hospital admissions, which may drive long term decline.

Pharmacological and non-pharmacological treatments are used to reduce COPD exacerbations[12,13], and improve care, such as pulmonary rehabilitation (PR[14]), azithromycin[15], inhaled bronchodilators and inhaled corticosteroids. Infection control measures during the pandemic reduced spread of

For peer review only

other respiratory viruses, which in turn was associated with a 43% fall in admissions, with benefits maintained post-lockdown[16] though this is not currently part of international COPD guidelines[17, 18]. SSMPs help patients but have not really shown significant impact on A&E visits or hospital admissions as demonstrated by a systematic review of self-management strategies for COPD[19]. Intuitively early recognition and treatment of AECOPD would reduce exacerbation severity and duration, and improve prognosis; evidence for this is limited but supportive[20,21].

This study looks to address the problem by personalising and thus optimising effectiveness of AECOPD management using the CE marked and MHRA registered solution, COPDPredict™[22].

Methods

Patient and public involvement

Throughout the design and delivery of the trial public and participant involvement (PPI) has been accessed. In particular independent members on the trial steering committee provide oversight and contributions to the trial, including design and standardisation of trial-specific SSMP. PPI involvement has also extended to the qualitative research where the topic guide was developed in conjunction with respiratory participant groups.

Trial Design

The Predict & Prevent AECOPD trial is a phase III, 2 arm, multi-centre, open label, parallel-group individually randomised clinical trial investigating the use of a personalised early warning decision support system (COPDPredict™) to predict and prevent acute exacerbations of COPD. We aim to recruit 384 participants from NHS hospitals in the UK and randomise each individual in a 1:1 ratio to either SSMP with RM (control arm) or COPDPredict™ with RM (intervention arm) (*Figure 1*). The start date for the trial was 30th January 2019 and the end date for the trial is 30th March 2023.

The main outcome measure is to provide further validation, as compared to usual care, for the clinical effectiveness of COPDPredict™ to help guide and support COPD patients and their respective clinical teams in identifying exacerbations early, with an aim to reduce the total number of AECOPD-

For peer review only

induced hospital admissions in the 12 months following each patient's randomisation. The trial includes a health economic analysis examining health-related quality of life (HRQoL) using the validated questionnaire EQ-5D-5L and healthcare utilisation (as determined by a questionnaire that inquires as to hospitalisations, GP attendances and medication usage). A qualitative sub-study will assess both the end user experience and the healthcare provider experience, using Normalization Process Theory to understand the implementation and integration of the new technology[23].

Eligibility Criteria

Inclusion Criteria

- Clinically diagnosed COPD, confirmed by post-bronchodilator spirometry and defined as Post bronchodilator Forced expiratory volume/Forced vital capacity (FEV_1/FVC) <0.7 and <lower limit of normal, in the stable state
- ≥1 AECOPD in any 12-month period within the last 2 years or ≥1 hospital admission for AECOPD in the previous two years.
- Exacerbation free for at least 6 weeks
- An age of at least 18 years
- Willing and able to comply with data collection to 12 months from randomisation
- Ability to consent
- Ability to use intervention as judged by the investigator at screening, upon demonstration of the system to the patient

Exclusion Criteria

- Life expectancy <12 months -Assessment the individual investigator
- Co-enrolment into any clinical trials of investigative medicinal product (CTIMPs)
- Patients with selected co-morbidities, where these could impair use of the intervention (eg dementia)

For peer review only

Outcomes

Primary Outcome

Number of hospital admissions at 12 months post randomisation where the primary reason for admission is AECOPD. This will be obtained from participant testimony and from centrally held (HES) records. Data from the 2 sources will be cross-referenced to remove double-counting. The total number of admissions will be the sum of unique admissions from the 2 sources of data

Secondary Outcomes

Clinical Outcomes

Over a 12-month period, following randomisation;

Total in participant days, Number of visits to A&E, Number of participant defined exacerbations, Appropriate action on symptoms of AECOPD, as shown by the app, or participant testimony, Health-related quality of life (CAT) and, FEV_1 at 12 months post randomisation (from spirometry obtained during study visit)

Economic Outcomes

Health-related quality of life at baseline and 3, 6, 9 and 12-months post randomisation (EQ-5D-5L)

Healthcare utilisation at 3, 6, 9 and 12-months post randomisation, as determined by a questionnaire that inquires on hospitalisations, GP attendances and medication usage

Qualitative sub-study

End user experience and provider experience

Control Arm

A standard self-management plan (SSMP) alongside use of rescue medication (RM) containing 5 days of antibiotic and steroid treatment.

For peer review only

Intervention

COPDPredict™, consists of a patient-facing App (iOS/Android) and clinician-facing dashboard[24]. The Early Warning Decision Support System uses longitudinal remote monitoring of relevant subjective and objective data (symptoms, spirometry, biomarkers) in real-time via the App which is connected to CE-marked Bluetooth-enabled sensor peripherals. These data are used to construct COPD-relevant individual profiles such that artificial intelligence artificial intelligence (AI)-driven algorithms can then identify changes in health status to provide timely individualised alerts to patients and clinicians and sign-posting to action plans for patients.

COPDPredict™ also provides information around COPD self-management, pulmonary rehabilitation, inhaler technique and utilises gamification to help with adherence. Patients have full access to their results and can also directly message their clinical team. COPDPredict™ may signpost a participant to a self-management action plan for exacerbation management but clinical team supervision and oversight will remain throughout, as per usual care.

The clinician facing dashboard allows for "real-time" case management with the ability to remotely monitor patients and facilitate interaction. Clinicians can choose to escalate treatments based on the results being transmitted by the patients. A crucial dimension to this advanced system is that blood biomarker measurements which inform an algorithm are incorporated to enhance accuracy. Blood-based point of care (POC) analysers are used with COPDPredict™ and these POC analysers have previously been validated against laboratory CRP measures.

Recruitment and Consent

All patients must provide fully informed consent. A Participant Information Sheet (PIS) which explains the different ways in which the participant may enter the trial is provided to participants to facilitate this process. Investigators or delegate(s) adequately explain the aim, trial intervention, anticipated benefits and potential hazards of taking part in the trial to the participant. They also stress that participation is voluntary and that the participant is free to refuse to take part and may

For peer review only

withdraw from the trial at any time. Any trial specific procedures are completed after the participant has given informed consent. At each visit the participant's willingness to continue in the trial is ascertained and documented in the medical notes. Where new information becomes available which may affect the participants' decision to continue, participants will be given time to consider and if happy to continue will be re-consented. Re-consent will be documented in the medical notes.

Randomisation

After informed consent has been obtained and full participant eligibility confirmed (including exacerbation free for 6 weeks), the participant can be randomised into the trial (Table 1). Randomisation CRFs are provided to investigators and may be used to collate the necessary information prior to randomisation. Participants are randomised by computer/telephone at the level of the individual in a 1:1 ratio to either SSMP with RM (control arm) or COPDPredict™ with RM (intervention arm).

A minimisation algorithm will be used within the online randomisation system to ensure balance in the treatment allocation over centre, age (<60, ≥60 years) and severity of disease as per Global Initiative for Obstructive Lung Disease (GOLD)[1].

The participants are randomised to one of the following:

<u>Control Arm</u>

A standard self-management plan (SSMP) alongside use of rescue medication (RM) containing 5 days of antibiotic and steroid treatment.

<u>Intervention arm</u>

Supported self-management using the COPDPredict™ App, involving personalised alerts to both patients and the clinical care team.

A 'random element' will be included in the minimisation algorithm, so that each participant has a probability, of being randomised to the opposite treatment that they would have otherwise

For peer review only

received. Once randomised patients will also be asked to complete baseline quality of life booklet which contains both the EQ-5D-5L and CAT questionnaire.

For peer review only

Assessment schedule

Visit	Hospital Discharge/ Initial appointment	Personal Baseline Day 1 after 6 weeks exacerbation free	Personal Baseline Day 7 (±2 days)	End of Personal Baseline Period	Approximately 4 times during any exacerbation (home visit or clinic)	Weekly until Week 52	Week 13 Telephone follow Up +/-1 week	Week 26 Hospital/ Telephone follow Up +/-1 week	Week 39 Telephone follow Up +/-1 week	Week 52 Telephone /Hospital follow Up +/- 2 weeks	Patients daily wellbeing completion post two-week bassline period
Eligibility check	X										
Valid informed consent	X										
Randomisation		X									
Height and Weight	X									X	
Concomitant medication	X	X		X				X	X	X	
Baseline medical history taken$^{\alpha}$	X										
Request to GP for rescue medication	X						(X)	(X)	(X)		
Assessment of (S)AEs	X				(X)		X	X	X	X	

For peer review only

Questionnaires (QoL and health utilisation)	X						X	X	X	X	
Spirometry	X							X		X	
Intervention only below this line						Intervention only below this line					
Provision of equipment & participant training		X									
Spirometry	X	X	X		(X)	X					
Symptom control markers	X	X	X	X	(X)	X	X	X	X	X	
Point of care blood test		X		X	(X)						
Clinical histories		X		X			X	X	X	X	
Wellbeing (App)											X

(X) denotes only when required. If rescue medication has not been used, fresh prescription requests will not be necessary

During the baseline period and any subsequent exacerbations patients will be entering well-being data on the App which will be related to lifestyle choices and the end-user experience. This data will not form part of the formal analysis of quality of life.

Table 1: Schedule of assessments

For peer review only

Statistical Analysis

Sample size

The justification of the sample size is based on previous evidence[25] that had shown a mean estimate of 2.5 COPD admissions in the previous year in the control group. To detect a difference of 1 admission in the mean number of admissions between groups using the standard methods of difference between means and assuming standard deviation of 2.6 with 90% power and a type I error rate of 5% (two-sided), 144 participants per group will need to be randomised, 288 in total. Assuming and adjusting for a 25% loss to follow-up/ drop-out rate, 384 participants will need to be recruited.

For peer review only

Analysis of Outcome Measures

The primary comparison groups will be composed of those randomised to the use of COPDPredict™ system and those randomised to the standard self-management plan (SSMP). All analyses will be based on the intention to treat principle[26], with all patients analysed in the treatment groups to which they were allocated irrespective of compliance with the randomised allocated treatment, and all patients will be included in the analyses. For all outcomes, summary statistics (e.g. mean differences, relative risks) will be reported and 95% confidence intervals will be constructed where appropriate. A two-sided p-value of <0.05 will be considered statistically significant.

Primary Outcome Measure

The primary analysis for this study will be to compare rates of hospital admission between the treatment groups (COPDPredict™ versus Usual care) over 12 months, following randomisation, where the primary reason for admission is an AECOPD event. These hospital admissions per person will be analysed using Poisson regression models adjusting for treatment group and minimisation variables. If there is over dispersion a negative binomial regression model adjusting for treatment group and the same minimisation variables will be taken into consideration. Point estimates

(incidence rates) will be provided and accompanied with 95% confidence intervals and respective p-values.

Secondary Outcome Measures

Recorded observations taken over a 12-month period, following randomisation:

Total in-hospital days and FEV_1 – These variables will be summarised using basic descriptive statistics (Mean, SD)). We may also consider a mixed linear regression model, to estimate differences between the intervention group supported with 95% CI adjusting for baseline variables and minimisation variables (centre being a random effects variable).

Health-related quality of life questionnaires - EQ5D-5L and CAT will be converted into scores and analysed using a mixed linear regression model, adjusting for the intervention group, baseline and minimisation variables as before.

Number of A&E visits, participant defined exacerbations and healthcare utilisation - Where the participant experienced an episode, the data during the 12 months from randomisation will be analysed using mixed effects log-binomial regression techniques with presentation of relative risk and 95% confidence intervals. Furthermore, sensitivity analysis based on patients' experience of multiple visits to A&E and participant defined exacerbation will be calculated using Poisson regression techniques and relative risk supported with 95% CI.

Symptom control marker association to clinical decision - The diagnostic accuracy of COPDPredict™ and Usual care will be evaluated by calculating sensitivity, specificity, positive and negative predictive values, and area under curve (AUC)/receiver operating characteristics (ROC) together with 95% CI.

Subgroup analyses

Subgroup analyses will be limited to the same variables used in the minimisation algorithm, apart from centre. Tests for statistical heterogeneity (e.g. by including the treatment group by subgroup

For peer review only - http://bmjopen.bmj.com/site/about/guidelines.xhtml

interaction parameter in the statistical model) will be performed prior to any examination of effect estimate within subgroups. The results of subgroup analyses will be treated with caution and will be used for the purposes of hypothesis generation only.

Missing data and sensitivity analyses

Participants with missing primary outcome data will not be included in the primary analysis in the first instance. This presents a risk of bias, and sensitivity analyses will be undertaken to assess the possible impact. This will consist of simulating the missing response using a multiple imputation approach[27]. Parameters used to simulate the missing response will include the minimisation variables, intervention group and previous response. Full details are included in the statistical analysis plan (SAP).

Adverse events and serious adverse events

Patients with COPD can have high disease burden, such that the trial population will be older, with co-morbidities such as osteoporosis[28], cardiovascular disease or raised cardiovascular risk[29], along with associated complications and symptoms including abnormal lab results. A relatively high number of adverse events are anticipated as a result of the patients' existing past medical history[30]. However, this study is examining the role of increased participant surveillance and therefore few, if any, foreseeable risks of direct harm associated with the study intervention are anticipated. AE reporting will therefore be limited to those events which are required for trial monitoring or outcome assessment.

All events which meet the definition of serious must be recorded in the participant notes, but for trial purposes these following events do not require reporting on the SAE Form. Such events are "safety reporting exempt".

• Planned hospitalisation for a pre-existing condition, or a procedure required by the trial protocol without serious deterioration in health, is not considered a serious adverse event.

For peer review only - http://bmjopen.bmj.com/site/about/guidelines.xhtml

• An overnight stay in hospital that is due to transportation, organisational or accommodation problems, and without medical background SAES requiring expedited reporting

Any other events that fulfil the usual definition of an SAE will be reported immediately.

Any death occurring during the Trial Protocol defined follow up period (12 months) must be reported as an SAE within 24 hours of the local investigator becoming aware of the event.

Health economics

Within -trial analysis

The economic evaluation alongside the trial will take the form of an incremental cost-effectiveness analysis to estimate cost per hospital admission avoided and incremental cost-utility analysis to estimate cost per quality adjusted life year (QALY) gained. Base-case analyses will be from a healthcare perspective over 12 months follow-up using individual participant level data on costs and outcomes from the clinical trial. Additional analyses will be undertaken from a broader societal perspective. Resource use data will be collected by questionnaire, capturing information on hospitalisations for exacerbations, COPD-related primary and secondary care attendances and medication usage. Data will also be collected during the trial on resource use implications of the intervention. Information on participant incurred costs, time off work and impact on activities is collected within the economic questionnaire. QALYs will be calculated from EQ-5D-5L responses. Sensitivity analysis will be undertaken and cost-effectiveness planes and cost-effectiveness acceptability curves presented.

Model-based analysis

A decision model will be constructed to extrapolate beyond trial results over five years, with costs and benefits discounted at 3.5%. The model will be the first decision analysis to use GOLD stages ABCD[1] and will consider the ability of the COPDPredict™ supported self-management to impact movements between GOLD ABCD stages, but also the number of exacerbations within those stages.

For peer review only - http://bmjopen.bmj.com/site/about/guidelines.xhtml

Costs associated with delivering COPDPredict™, exacerbations, and routine care will be obtained from the trial. Quality of life scores on each intervention will also be obtained from trial data using EQ-5D-5L. Longer-term costs, quality of life scores, and transitions between stages, and number of exacerbations will be obtained from analysis of ABCD cohorts from within the BLISS[2] study, a prospective cohort study with 3-year follow-up period. The model will estimate cost per QALY gained of COPDPredict™ supported self-management versus standard self-management from a healthcare perspective. Probabilistic sensitivity analysis will be undertaken to simultaneously incorporate all parameter uncertainty. Cost-effectiveness planes and cost-effectiveness acceptability curves will be presented to show the probability the intervention is cost-effective at different cost/QALY thresholds. A value of information analysis will assess the value of further trials to reduce parameter uncertainty and identify parameters which would be most valuable in reducing that uncertainty[31].

Qualitative sub-study

In order to frame this study, we draw on Normalisation Process Theory[32], which proposes four constructs essential to effect implementation of new technologies: *coherence* (sense making work to understand the possibilities of a new technology), *cognitive participation* (relational work as new technology begins to be used), *collective action* (operational work enacted to make a technology function in context) and *reflexive monitoring* (the appraisal of work that people do to assess how the new technology affects them and others). Participants will include health care professionals, patients and wider policy stakeholders and will be purposively sampled to maximise diversity. A topic guide developed by drawing on existing literature and theory will focus on attitudes to and practices around self-management of COPD and the use and implementation of digital technologies. Interviews will be audio recorded and transcribed verbatim, before subjecting the data to a two-stage approach to analysis[33]: first, a descriptive framework analysis[34] followed by mapping onto the NPT constructs. We anticipate conducting approximately30 interviews but this will be informed by the data and analysis[35].

For peer review only

Ethics and dissemination:

This study protocol is reported in accordance with the guidance set out in the SPIRIT statement. Predict and Prevent AECOPD has obtained ethical approval in England (19/LO/1939). Upon completion of the trial and publication of results a lay findings summary will be disseminated to trial participants.

Trial Oversight

Trial oversight will be provided by the TMG, an independent DMC and independent TSC. Interim analyses of major outcome measures and safety data will be conducted and provided in strict confidence to the DMC.

Potential Impact

Predict & Prevent AECOPD is a pragmatic, national, multicentre, RCT which aims to provide robust evidence around the clinical and cost effectiveness of COPDPredict™ by analysing the number of patients admitted to hospital in a 12month period post randomisation. It is hoped the outcome of this trial could be used to provide robust evidence to the management of patients with COPD and their exacerbations in the future.

Contributorship statement

All authors have made substantial contributions to the conception or design of the work, or the acquisition, analysis or interpretation of data. DK, AT, HJ, RM, SJ, NG, NP and MS drafted the manuscript and revised it critically for important intellectual content. All authors approved final version and agreed to be accountable for all aspects of the work in ensuring that questions related to the accuracy or integrity of any part of the work are appropriately investigated and resolved.

Competing Interest Statement

The CI of the trial (AT) does not have any relevant direct financial disclosures, nor do members of the trial management group (TMG) who are authors of this paper, with the exception of NP who is a

For peer review only

founder, director and share-holder of NEPeSMO, who developed the intervention. In addition, MS is CI of the overall NIHR-funded project and is a founder, director and share-holder of NEPeSMO. NEPeSMO is a start-up company from the University Hospitals of North Midlands NHS Trust, owns all intellectual property rights of COPDPredict™ and is a project collaborator on the grant.

AT has grants from pharmaceutical companies working in the area of COPD (Chiesi, AstraZeneca) and has conducted advisory work for such (Boehringer, CSL Behring) but not in the area of medical devices or admission prevention. Neither has she worked for, or received monies from, any company working on admission prevention in the last 3 years.

Funding Statement & Disclaimer

This report is independent research funded by the National Institute for Health Research (Invention for Innovation, A Personalised Early Warning Decision Support System with novel Saliva Bio-Profiling to Predict and Prevent Acute Exacerbations of Chronic Obstructive Pulmonary Disease – 'Predict&prevent AECOPD, NIHR200002).

For peer review only

References

1. Global Initiative for Obstructive Lung Disease, [Accessed 13 June 2018]. [Available from: www.goldcopd.com].

2. Estimating the economic burden of respiratory illness in the UK. London: British Lung Foundation 2016.

3. The battle for breath - the impact of lung disease in the UK. London: British Lung Foundation, 2016.

4. Roberts CM, Stone RA, Lowe D, Pursey NA, Buckingham RJ. Co-morbidities and 90-day outcomes in hospitalized COPD exacerbations. Copd. 2011;8(5):354-61.

5. Stone RA HB, Lowe D, Searle L, Skipper E, Welham S, et al. . National COPD audit programme. COPD: Who cares matters. London, UK: Royal College of Physicians, 2015. Contract No.: eISBN 978-1-86016-559-7.

6. Hurst JR, Vestbo J, Anzueto A, Locantore N, Mullerova H, Tal-Singer R, et al. Susceptibility to exacerbation in chronic obstructive pulmonary disease. N Engl J Med. 2010;363(12):1128-38.

7. Merinopoulou E, Raluy-Callado M, Ramagopalan S, MacLachlan S, Khalid JM. COPD exacerbations by disease severity in England. Int J Chron Obstruct Pulmon Dis. 2016;11:697-709.

8. Stone RA HB, McMillan V, Saleem Khan M, Searle L, Skipper E, et al. COPD: Who cares when it matters most? London: Royal College of Physicians, 2017.

9. Steer J, Gibson GJ, Bourke SC. Longitudinal change in quality of life following hospitalisation for acute exacerbations of COPD. BMJ Open Respir Res. 2015;2(1):e000069.

10. Jo YS, Kim SK, Park SJ, Um S-J, Park Y-B, Jung KS, et al. Longitudinal change of FEV(1) and inspiratory capacity: clinical implication and relevance to exacerbation risk in patients with COPD. Int J Chron Obstruct Pulmon Dis. 2019;14:361-9.

For peer review only

11. Qureshi H SA, Hanania NA. Chronic obstructive pulmonary disease exacerbations: latest evidence and clinical implications. Therapeutic advances in chronic disease. 2014;5(5):212-27. Epub 2014/09/02.

12. Oba, Y., Keeney, E., Ghatehorde, N. and Dias, S., 2018. Dual combination therapy versus long-acting bronchodilators alone for chronic obstructive pulmonary disease (COPD): a systematic review and network meta-analysis. Cochrane Database of Systematic Reviews, [online] 2018(12). Available at: <https://www.cochranelibrary.com/cdsr/doi/10.1002/14651858.CD012620.pub2/full> [Accessed 5 October 2021].

13. Yang, I., Clarke, M., Sim, E. and Fong, K., 2012. Inhaled corticosteroids for stable chronic obstructive pulmonary disease. Cochrane Database of Systematic Reviews, [online] Available at: <http://Inhaled corticosteroids for stable chronic obstructive pulmonary disease> [Accessed 1 September 2021].

14. Ryrso CK, Godtfredsen NS, Kofod LM, Lavesen M, Mogensen L, Tobberup R, et al. Lower mortality after early supervised pulmonary rehabilitation following COPD-exacerbations: a systematic review and meta-analysis. BMC Pulm Med. 2018;18(1):154.

15. Ni, W., Shao, X., Cai, X., Wei, C., Cui, J., Wang, R. and Liu, Y., 2015. Prophylactic Use of Macrolide Antibiotics for the Prevention of Chronic Obstructive Pulmonary Disease Exacerbation: A Meta-Analysis. PLOS ONE, 10(3), p.e0121257.

16. Lawless, M., Burgess, M. and Bourke, S., 2022. Impact of COVID-19 on Hospital Admissions for COPD Exacerbation: Lessons for Future Care. Medicina, 58(1), p.66.

17. Nice.org.uk. 2018. Overview | Chronic obstructive pulmonary disease in over 16s: diagnosis and management | Guidance | NICE. [online] Available at: <https://www.nice.org.uk/guidance/ng115> [Accessed 23 July 2022].

18. Global Initiative for Chronic Obstructive Lung Disease - GOLD. 2022. 2022 GOLD Reports - Global Initiative for Chronic Obstructive Lung Disease - GOLD. [online] Available at: <https://goldcopd.org/2022-gold-reports-2/> [Accessed 23 July 2022].

19. Jolly K, Majothi S, Sitch AJ, Heneghan NR, Riley RD, Moore DJ, et al. Self-management of health care behaviors for COPD: a systematic review and meta-analysis. Int J Chron Obstruct Pulmon Dis. 2016;11:305-26.

20. Vijayasaratha K, Stockley RA. Relationship between frequency, length, and treatment outcome of exacerbations to baseline lung function and lung density in alpha-1 antitrypsin-deficient COPD. Int J Chron Obstruct Pulmon Dis. 2012;7:789-96.

21. Celli BR, Locantore N, Yates J, Tal-Singer R, Miller BE, Bakke P, et al. Inflammatory biomarkers improve clinical prediction of mortality in chronic obstructive pulmonary disease. Am J Respir Crit Care Med. 2012;185(10):1065-72.

For peer review only - http://bmjopen.bmj.com/site/about/guidelines.xhtml

22. Patel N, Kinmond K, Jones P, Birks P, Spiteri MA. Validation of COPDPredict™: Unique Combination of Remote Monitoring and Exacerbation Prediction to Support Preventative Management of COPD Exacerbations. International Journal of Chronic Obstructive Pulmonary Disease 2021, 16:1887-1899

23. May CR, Mair F, Finch T, MacFarlane A, Dowrick C, Treweek S, Rapley T, Ballini L, Ong BN, Rogers A, Murray E. Development of a theory of implementation and integration: Normalization Process Theory. Implementation Science. 2009 Dec;4(1):1-9.

24. Nepesmo.com. 2017. NEPeSMO – Predict to Prevent. [online] Available at: <https://nepesmo.com/#:~:text=NEPeSMO%20offers%20a%20smart%20CE,to%20patients%20living%20with%20COPD> [Accessed 16 June 2021].

25. Pinnock H, Hanley J, McCloughan L, Todd A, Krishan A, Lewis S, et al. Effectiveness of telemonitoring integrated into existing clinical services on hospital admission for exacerbation of chronic obstructive pulmonary disease: researcher blind, multicentre, randomised controlled trial. Bmj. 2013;347:f6070

26. Sandeep K Gupta. Intention-to-treat concept: A Review. Perspect Clin Res 2011:2(3);109-112

27 . White IR, Royston P, Wood AM. Multiple imputation using chained equations. Stats in Medicine 2011, (30) 377-399.

28. Vrieze A, de Greef MH, Wijkstra PJ, Wempe JB. Low bone mineral density in COPD patients related to worse lung function, low weight and decreased fat-free mass. Osteoporos Int. 2007;18(9):1197-202.

29. Patel AR, Kowlessar BS, Donaldson GC, Mackay AJ, Singh R, George SN, et al. Cardiovascular risk, myocardial injury, and exacerbations of chronic obstructive pulmonary disease. Am J Respir Crit Care Med. 2013;188(9):1091-9.

30. Miller J, Edwards LD, Agusti A, Bakke P, Calverley PM, Celli B, et al. Comorbidity, systemic inflammation and outcomes in the ECLIPSE cohort. Respir Med. 2013;107(9):1376-84.

31. Global Initiative for Obstructive Lung Disease COPD Diagnosis and Management At-A-Glance Desk Reference 2017 [online] https://goldcopd.org/archived-reports/ Accessed 03/09/21

32. May C, Finch T. Implementing, Embedding, and Integrating Practices: An Outline of Normalization Process Theory. Sociology. 2009;43(3):535-54.

33. Ritchie J, Spencer L. Qualitative data analysis for applied policy research. In: Bryman A, Burgess RG, editors. London: Routledge; 1994.

34. Macfarlane A, O'Reilly-de Brún M. Using a theory-driven conceptual framework in qualitative health research. Qual Health Res. 2012;22(5):607-18.

For peer review only

35. O'Cathain A, Hoddinott P, Lewin S, Thomas KJ, Young B, Adamson J, et al. Maximising the impact of qualitative research in feasibility studies for randomised controlled trials: guidance for researchers. Pilot and Feasibility Studies. 2015;1(1):32.

Figure Legends

Figure 1: Flow diagram of the Predict & Prevent trial

Table Legends

Table 1: Schedule of assessments

For peer review only

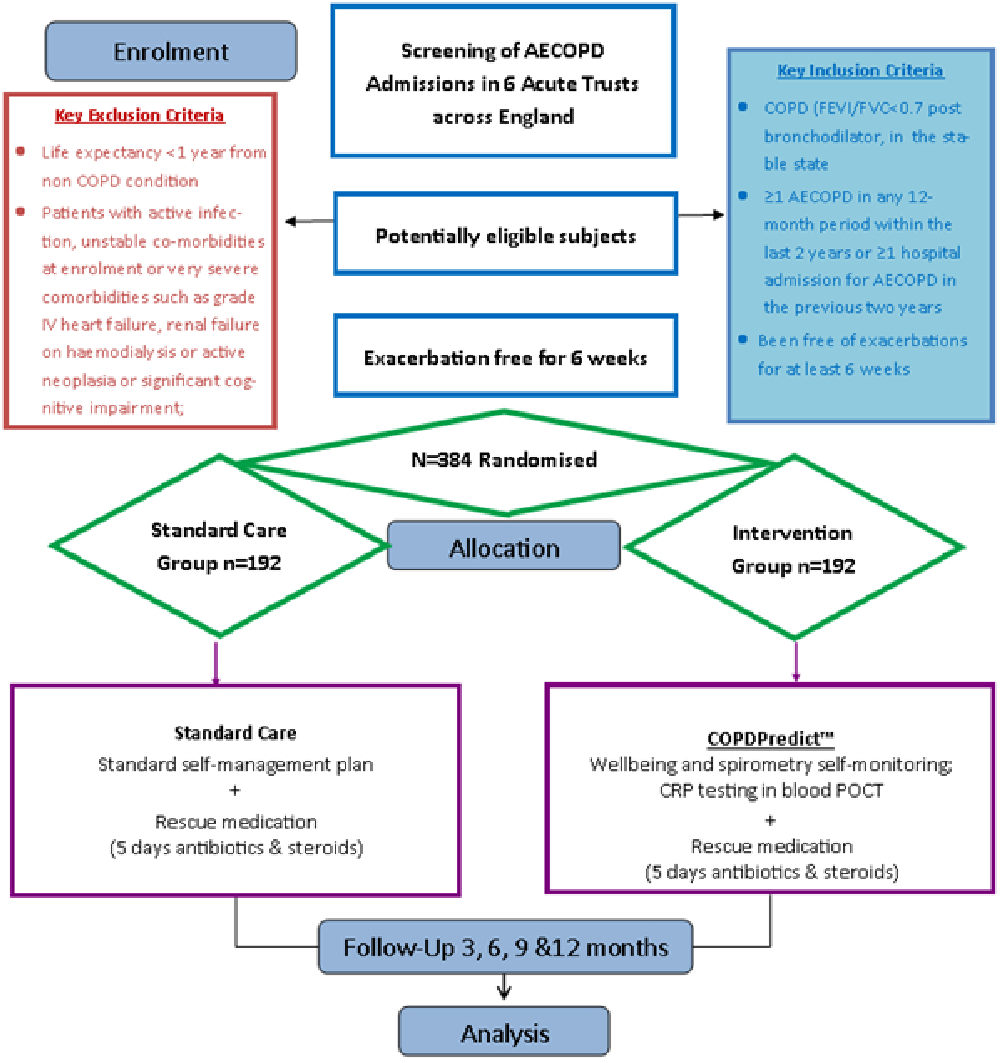

Figure 1: Flow diagram of the Predict & Prevent trial

266x297mm (57 x 57 DPI)

SPIRIT

STANDARD PROTOCOL ITEMS: RECOMMENDATIONS FOR INTERVENTIONAL TRIALS

SPIRIT 2013 Checklist: Recommended items to address in a clinical trial protocol and related documents*

Section/item	ItemNo	Description	Section of manuscript
**Administrative information**			
Title	1	Descriptive title identifying the study design, population, interventions, and, if applicable, trial acronym	Page 5 methods
Trial registration	2a	Trial identifier and registry name. If not yet registered, name of intended registry	Page 2 abstract
	2b	All items from the World Health Organization Trial Registration Data Set	
Protocol version	3	Date and version identifier	V9.0 vd03-Mar-22
Funding	4	Sources and types of financial, material, and other support	Page 2 NIHR Statement
Roles and responsibilities	5a	Names, affiliations, and roles of protocol contributors	Page 1
	5b	Name and contact information for the trial sponsor	Page 1 and 2
	5c	Role of study sponsor and funders, if any, in study design; collection, management, analysis, and interpretation of data; writing of the report; and the decision to submit the report for publication, including whether they will have ultimate authority over any of these activities	

For peer review only

	5d	Composition, roles, and responsibilities of the coordinating centre, steering committee, endpoint adjudication committee, data management team, and other individuals or groups overseeing the trial, if applicable (see Item 21a for data monitoring committee)	
**Introduction**			
Background and rationale	6a	Description of research question and justification for undertaking the trial, including summary of relevant studies (published and unpublished) examining benefits and harms for each intervention	Page 4-5
	6b	Explanation for choice of comparators	
Objectives	7	Specific objectives or hypotheses	Page 5
Trial design	8	Description of trial design including type of trial (eg, parallel group, crossover, factorial, single group), allocation ratio, and framework (eg, superiority, equivalence, noninferiority, exploratory)	Page 5-6
**Methods: Participants, interventions, and outcomes**			
Study setting	9	Description of study settings (eg, community clinic, academic hospital) and list of countries where data will be collected. Reference to where list of study sites can be obtained	Page 5

For peer review only

Eligibility criteria	10	Inclusion and exclusion criteria for participants. If applicable, eligibility criteria for study centres and individuals who will perform the interventions (eg, surgeons, psychotherapists)	Page 6
Interventions	11a	Interventions for each group with sufficient detail to allow replication, including how and when they will be administered	Page 7
	11b	Criteria for discontinuing or modifying allocated interventions for a given trial participant (eg, drug dose change in response to harms, participant request, or improving/worsening disease)	N/A
	11c	Strategies to improve adherence to intervention protocols, and any procedures for monitoring adherence (eg, drug tablet return, laboratory tests)	N/A
	11d	Relevant concomitant care and interventions that are permitted or prohibited during the trial	N/A
Outcomes	12	Primary, secondary, and other outcomes, including the specific measurement variable (eg, systolic blood pressure), analysis metric (eg, change from baseline, final value, time to event), method of aggregation (eg, median, proportion), and time point for each outcome. Explanation of the clinical relevance of chosen efficacy and harm outcomes is strongly recommended	Page 6/7

For peer review only - http://bmjopen.bmj.com/site/about/guidelines.xhtml

Participant timeline	13	Time schedule of enrolment, interventions (including any run-ins and washouts), assessments, and visits for participants. A schematic diagram is highly recommended (see Figure)	Page 10
Sample size	14	Estimated number of participants needed to achieve study objectives and how it was determined, including clinical and statistical assumptions supporting any sample size calculations	Page 5
Recruitment	15	Strategies for achieving adequate participant enrolment to reach target sample size	Page 8

Methods: Assignment of interventions (for controlled trials)

Allocation:

Sequence generation	16a	Method of generating the allocation sequence (eg, computer-generated random numbers), and list of any factors for stratification. To reduce predictability of a random sequence, details of any planned restriction (eg, blocking) should be provided in a separate document that is unavailable to those who enrol participants or assign interventions	Page 8 and 9
Allocation concealment mechanism	16b	Mechanism of implementing the allocation sequence (eg, central telephone; sequentially numbered, opaque, sealed envelopes), describing any steps to conceal the sequence until interventions are assigned	Protocol

Implementation	16c	Who will generate the allocation sequence, who will enrol participants, and who will assign participants to interventions	Protocol
Blinding (masking)	17a	Who will be blinded after assignment to interventions (eg, trial participants, care providers, outcome assessors, data analysts), and how	N/A
	17b	If blinded, circumstances under which unblinding is permissible, and procedure for revealing a participant's allocated intervention during the trial	
**Methods: Data collection, management, and analysis**			
Data collection methods	18a	Plans for assessment and collection of outcome, baseline, and other trial data, including any related processes to promote data quality (eg, duplicate measurements, training of assessors) and a description of study instruments (eg, questionnaires, laboratory tests) along with their reliability and validity, if known. Reference to where data collection forms can be found, if not in the protocol	Page 12-16 and Protocol
	18b	Plans to promote participant retention and complete follow-up, including list of any outcome data to be collected for participants who discontinue or deviate from intervention protocols	Page 12-13

Data management	19	Plans for data entry, coding, security, and storage, including any related processes to promote data quality (eg, double data entry; range checks for data values). Reference to where details of data management procedures can be found, if not in the protocol	Protocol
Statistical methods	20a	Statistical methods for analysing primary and secondary outcomes. Reference to where other details of the statistical analysis plan can be found, if not in the protocol	Protocol
	20b	Methods for any additional analyses (eg, subgroup and adjusted analyses)	Page 15-16
	20c	Definition of analysis population relating to protocol non-adherence (eg, as randomised analysis), and any statistical methods to handle missing data (eg, multiple imputation)	Page 14
**Methods: Monitoring**			
Data monitoring	21a	Composition of data monitoring committee (DMC); summary of its role and reporting structure; statement of whether it is independent from the sponsor and competing interests; and reference to where further details about its charter can be found, if not in the protocol. Alternatively, an explanation of why a DMC is not needed	Protocol

	21b	Description of any interim analyses and stopping guidelines, including who will have access to these interim results and make the final decision to terminate the trial	Protocol
Harms	22	Plans for collecting, assessing, reporting, and managing solicited and spontaneously reported adverse events and other unintended effects of trial interventions or trial conduct	Protocol
Auditing	23	Frequency and procedures for auditing trial conduct, if any, and whether the process will be independent from investigators and the sponsor	
**Ethics and dissemination**			
Research ethics approval	24	Plans for seeking research ethics committee/institutional review board (REC/IRB) approval	Page 16
Protocol amendments	25	Plans for communicating important protocol modifications (eg, changes to eligibility criteria, outcomes, analyses) to relevant parties (eg, investigators, REC/IRBs, trial participants, trial registries, journals, regulators)	Protocol
Consent or assent	26a	Who will obtain informed consent or assent from potential trial participants or authorised surrogates, and how (see Item 32)	Protocol
	26b	Additional consent provisions for collection and use of participant data and biological specimens in ancillary studies, if applicable	

For peer review only

Confidentiality	27	How personal information about potential and enrolled participants will be collected, shared, and maintained in order to protect confidentiality before, during, and after the trial	Protocol
Declaration of interests	28	Financial and other competing interests for principal investigators for the overall trial and each study site	Page 17
Access to data	29	Statement of who will have access to the final trial dataset, and disclosure of contractual agreements that limit such access for investigators	Protocol
Ancillary and post-trial care	30	Provisions, if any, for ancillary and post-trial care, and for compensation to those who suffer harm from trial participation	N/A
Dissemination policy	31a	Plans for investigators and sponsor to communicate trial results to participants, healthcare professionals, the public, and other relevant groups (eg, via publication, reporting in results databases, or other data sharing arrangements), including any publication restrictions	Page 16
	31b	Authorship eligibility guidelines and any intended use of professional writers	
	31c	Plans, if any, for granting public access to the full protocol, participant-level dataset, and statistical code	N/A

Appendices

For peer review only - http://bmjopen.bmj.com/site/about/guidelines.xhtml

Informed consent materials	32	Model consent form and other related documentation given to participants and authorised surrogates	ICF
Biological specimens	33	Plans for collection, laboratory evaluation, and storage of biological specimens for genetic or molecular analysis in the current trial and for future use in ancillary studies, if applicable	N/A

*It is strongly recommended that this checklist be read in conjunction with the SPIRIT 2013 Explanation & Elaboration for important clarification on the items. Amendments to the protocol should be tracked and dated. The SPIRIT checklist is copyrighted by the SPIRIT Group under the Creative Commons "Attribution-NonCommercial-NoDerivs 3.0 Unported" license.

For peer review only
